# Tribute to Kenneth Sauer (1931–2022): a mentor, a role-model, and an inspiration to all in the field of photosynthesis

**DOI:** 10.1007/s11120-024-01119-0

**Published:** 2024-11-13

**Authors:** Junko Yano, Jan Kern, Robert E. Blankenship, Johannes Messinger, Vittal K. Yachandra

**Affiliations:** 1 Molecular Biophysics and Integrated Bioimaging Division, Lawrence Berkeley National Laboratory, Berkeley, CA 94720 USA; 2 Departments of Biology and Chemistry, Washington University in St. Louis, St. Louis, MO 63130 USA; 3 Molecular Biomimetics, Department of Chemistry-Ångström Laboratory, Uppsala University, 75120 Uppsala, SE Sweden; 4 Umeå Plant Science Centre, Department of Plant Physiology, Umeå University, 90187 Umeå, SE Sweden

**Keywords:** Photosynthesis, Biography, Ken Sauer, Tribute

## Abstract

Kenneth (Ken) Sauer was a mainstay of research in photosynthesis at the University of California, Berkeley and the Lawrence Berkeley National Laboratory (LBNL) for more than 50 years. Ken will be remembered by his colleagues, and other workers in the field of photosynthesis as well, for his pioneering work that introduced the physical techniques whose application have enriched our understanding of the basic reactions of oxygenic photosynthesis. His laboratory was a training ground for many students and postdocs who went on to success in the field of photosynthesis and many others. Trained as a physical chemist, he always brought that quantitative approach to research questions and used several spectroscopic methods in his research. His broad scientific interests concerned the role of manganese in oxygen evolution, electronic properties of chlorophylls, energy transport in antenna complexes, and electron transport reactions. He was also an enthusiastic teacher, an enormously successful mentor who leaves behind a legion of scientists as his abiding legacy, a lover of music and the outdoors with many interests beyond science, and a dedicated family man with a great sense of humility. In this tribute, we summarize some aspects of Ken Sauer’s life and career, illustrated with selected research achievements, and describe his approach to research and life as we perceived it, which is complemented by reminiscences of several current researchers in photosynthesis and other fields. The supporting material includes Ken Sauers’s CV and publication list, as well as a list of the graduate students and postdocs he trained and of researchers that spent a sabbatical in his lab.

**Supplementary Information:**

The online version contains supplementary material available at 10.1007/s11120-024-01119-0.

## Early life

**Fig. 1:**
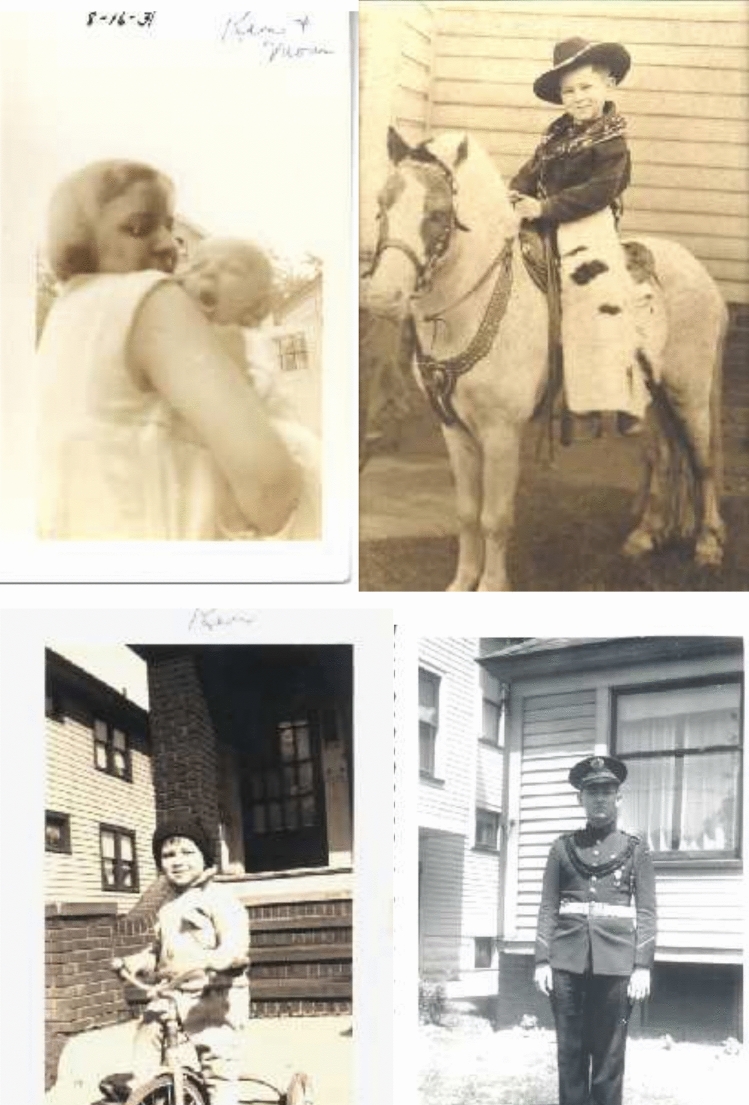
*Top Left*: Ken ~ 2 months old with Mom (1931); *Top right*: Ken as a little boy on a pony; *Bottom left*: Ken on a tricycle. Another of his passions, bicycling, that lasted his whole life. He always commuted from his house in the Berkeley hills to the lab on his bicycle and almost never used his car for the commute; *Bottom right*: In the school band—music was always important for Ken, even in the later part of his life. Photos courtesy of Margie Sauer

**Fig. 2:**
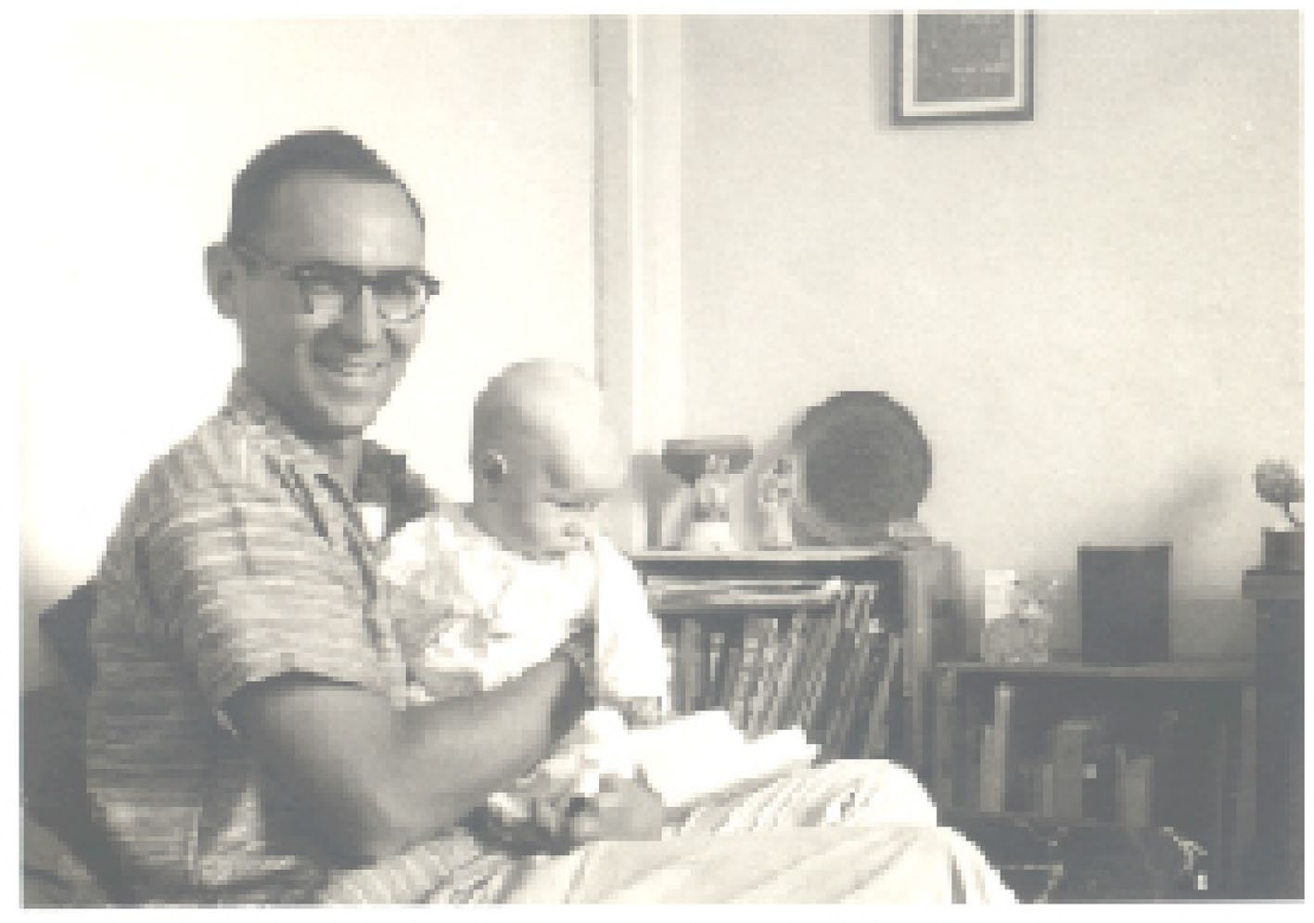
Ken in Beirut with his first son Bob (1959). Ken had taken a teaching position in the American University in Beirut before his Ph.D. from Harvard. Photo courtesy of Margie Sauer

Ken Sauer was born in Cleveland, Ohio in 1931, where his father worked for the railroad, and he had a normal middle-class upbringing with typical childhood pursuits (Fig. 1). He went to Oberlin College in Ohio, where he graduated in 1953 with an A.B. in Chemistry. His graduate work was done at Harvard, where he worked under the direction of George Kistiakowsky, a noted physical chemist (Kistiakowsky and Sauer 1956, 1958). Kistiakowsky had been part of the Manhattan Project during World War II and later served as the Presidential Science Advisor for President Dwight Eisenhower. Ken’s thesis work at Harvard was on gas-phase photochemistry of methylene. In 1957, a year before he graduated, Ken took a teaching position at the American University of Beirut in Lebanon, where he stayed for three years (Fig. 2). It is in Beirut where he met his wife Margie, who was teaching at the time in the American Community School. Appropriately enough, they met while singing, which became a life-long passion that continued on all through their life at Berkeley. Ken fondly used to remember how they got married and travelled all over the region, especially Turkey, often by motor scooter, and it was here that their first son Bob was born, and subsequently they had three other sons, Rodney, Terry, and Peter.

## Career at Berkeley

**Fig. 3:**
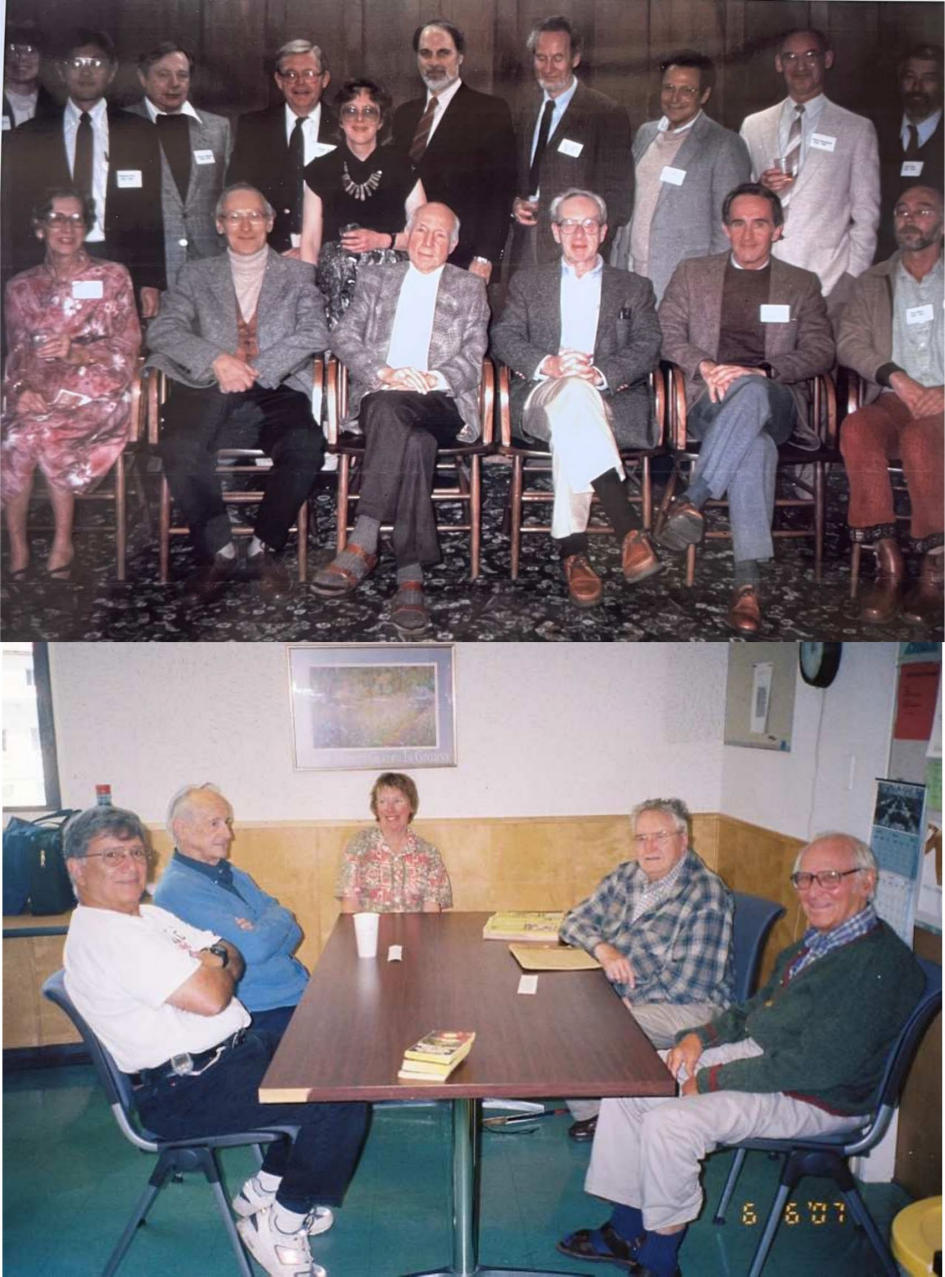
*Top*: Ken much later in life (~ 1990s) with Calvin and Mel Klein seated in the middle. Photo from Ken’s collection. *Bottom*: Ken on the right in 2007 with Al Bassham (second from left) in the Calvin Lab lunch room. Al Bassham worked with Calvin in solving the pathway of carbon in the reduction of CO_2_ to starch which Calvin received the Nobel prize for in 1961. The ‘old timers’ as they called themselves met periodically in the Calvin Lab even after they had retired from active research many years earlier. Others are Ray and Marie Alberti, and Ed Bennett (to the right of Ken) all worked in the Calvin Lab. Photo courtesy of Vittal Yachandra

In 1960 Ken Sauer returned to the US and accepted a postdoctoral position at UC Berkeley in the lab of Melvin Calvin, where he was introduced to photosynthesis research and met some of the leading figures in this field of research. Ken reminisced about this step in a Catalyst Magazine article published in 2016 by the College of Chemistry, UC Berkeley, “Although Lebanon was hard to leave, after three years I thought that it was time to return to the United States. Colleagues described Berkeley as the Beirut of the west coast of the U.S., so I applied and was offered a postdoctoral opportunity working with Melvin Calvin. By the time I arrived, most of Calvin’s Nobel Prize-winning research had already been conducted. He had assembled a large group of grad students and postdocs during the 1950s to attempt to use carbon-14, which was first synthesized at LBNL in the 1940s, to understand the chemical pathways used by plants to conduct photosynthesis and assimilate carbon dioxide from the atmosphere.” Fig. 3 top shows Ken with Calvin and other members of the Calvin Lab, and with Al Bassham (Fig. 3 bottom), a key figure in the discoveries that lead to Calvin’s Nobel prize, with whom he continued to meet in the Calvin Lab for lunch regularly long past their retirements. Ken was surely influenced by Calvin’s ‘interdisciplinary’ approach to research as exemplified by the Calvin Lab (see below), (which was then part of the Chemical Biodynamics Division at LBNL, a name that Calvin had come up with for interdisciplinary research), even before such a word probably was used to describe such research.

Ken’s work with Calvin was primarily spectroscopic in nature and utilized his basic knowledge of photochemistry and physical chemistry to address the problem of the early events of photosynthesis. Sauer co-authored his first article with Calvin in 1962, which was carried out along with Roderic Park in Berkeley’s botany department, on the spectroscopic properties of photosynthetic membranes from spinach chloroplasts (Sauer and Calvin 1962b, a). Ken also met John Biggins during this time with Calvin, and Ken remembered later how Roderic Park (Sauer and Park 1965; Park et al. 1966) and John Biggins (Biggins and Sauer 1964; Sauer and Biggins 1965) thrived together in Berkeley, and about their friendship that lasted their lifetimes, specifically when John returned to the Bay area after his retirement from Brown University and started making wine in Sonoma (Bruce and Sauer 2005).

**Fig. 4:**
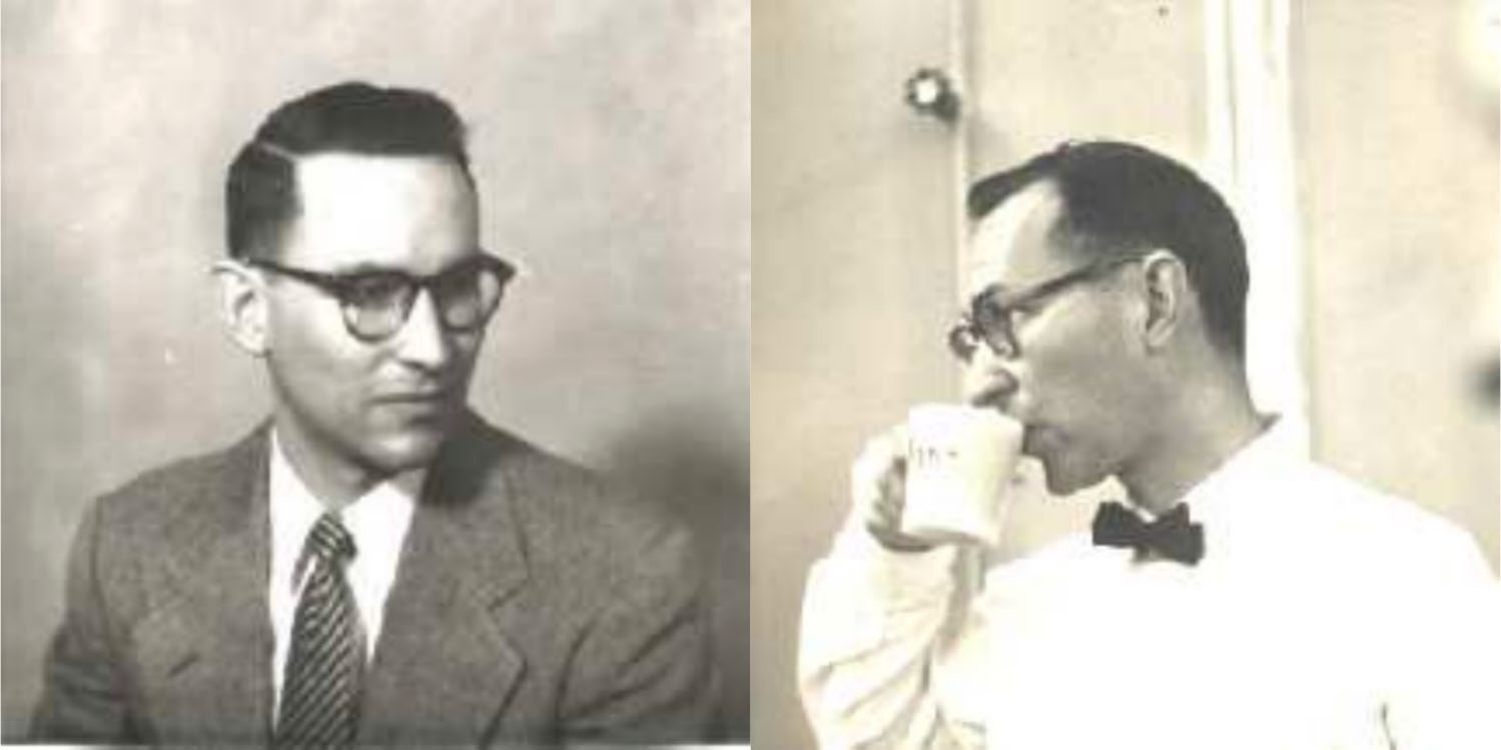
*Left*: Ken as an Assistant Professor at UC Berkeley (~ 1963); *Right*: Ken at the sacrosanct coffee breaks at the Calvin Lab—a Melvin Calvin instituted ritual at 10 am every day—more important ideas probably came from these breaks than one can imagine. Photos courtesy of Margie Sauer

Ken joined the faculty of the Chemistry Department of UC Berkeley in 1963 (Fig. 4) and remained there until his retirement in 2001, 41 years since he came to Berkeley, and more than 240 papers later. During that period, he mentored well over a hundred graduate students, postdoctoral scholars and visiting scientists (listed in the Supplemental Material). He also taught thousands of Berkeley undergraduate students in Honors General Chemistry and Introductory Physical Chemistry for Life Sciences.

After his retirement from being a professor in 2001, he continued to be active in the College of Chemistry, UC Berkeley, and with the photosynthesis group at LBNL, which had moved from the Calvin Lab to a hill site in 2007. Ken remained active at the Lab as a senior affiliate in the YYK (Yano, Yachandra, Kern) Group in the Molecular Biophysics and Integrated Bioimaging (MBIB) Division in LBNL. It was quite natural for him to continue working and he remained a great resource for the group, and as always he was dedicated to science and teaching the younger generation about the physical principles involved in photosynthesis. His way to keep fresh and active was to talk to the young people at the lab and be a role model, and to follow up on new scientific pursuits. He became, for example, very interested in the origin of the manganese cluster in photosystem II (Sauer and Yachandra 2002) and was particularly excited about the opportunities presented by genomic sequencing by the Joint Genome Institute in Berkeley. He continued to publish in retirement with this group, and he was the bridge between the current generation of photosynthesis researchers at UC Berkeley (Graham Fleming, Kris Niyogi) and LBNL (Vittal Yachandra, Junko Yano, and Jan Kern) and the days of the Calvin research group. Although his active participation decreased starting from 2008, he enjoyed coming to the group meetings and lunches, and group activities. He continued coming to the lab sometimes until 2019 before the pandemic (see pictures below). He was always a great resource for guidance and advice for his colleagues and was a role model on how to be a good scientist and, more importantly, also be a good person. A CV capturing the basics of his career is included in the Supplemental Information.

## The Calvin Lab–a place for interdisciplinary research

**Fig. 5:**
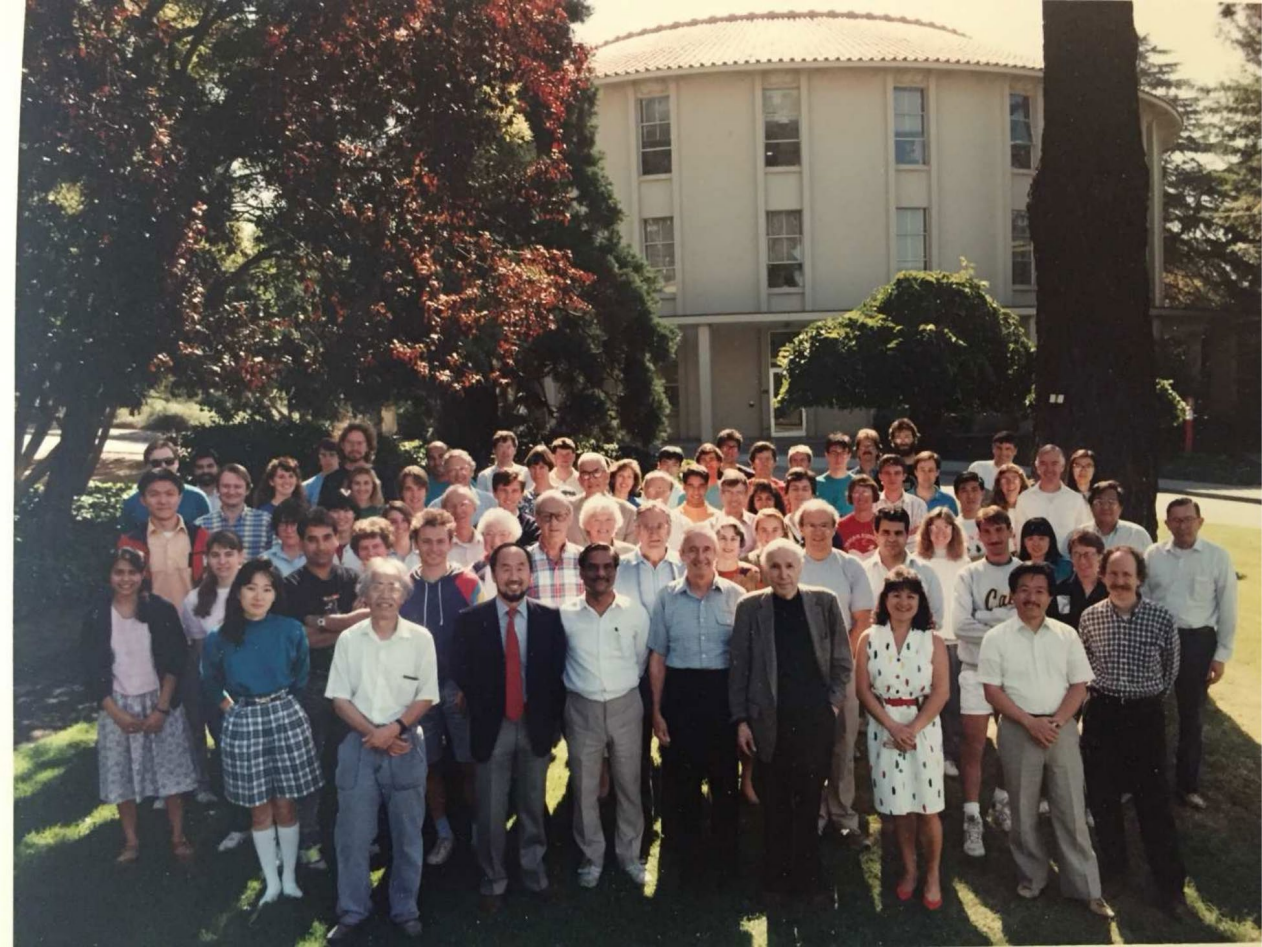
Melvin Calvin Laboratory (~ 1990 on the occasion of the dedication of the building as the Melvin Calvin Building) – an unique circular building on the UC Berkeley campus was also a part of Lawrence Berkeley National Laboratory until 2007 when the LBNL Division, now called the Molecular Biophysics and Integrated Bioimaging Division moved to a hill location. Interestingly, the Director of the Division is now Junko Yano, who started as a postdoc in 2001

Most of Ken’s research delving into the many aspects of photosynthesis was performed in the Calvin Lab, a circular building erected in 1964 that is located between the law school and the business school on UC Berkeley’s campus (Fig. 5). The building, which was part of Berkeley Lab, was designed by Calvin to encourage creative collaboration between groups arrayed around the center, which held shared equipment. Later, starting in 2007, the group moved to building 66 on the LBNL hill campus.

Some select people, who were Ken’s students and postdocs and colleagues, are identified: *First row*: from left 2nd Ning Pon, 3rd Sung-Hou Kim, 4th and 5th Al Bassham and Melvin Calvin, 7th and 8th Masami Kusunoki and David Wemmer. *Second row*: from left 1st Ishita Mukerji, 7th John Otvos, *Third row*: from left, 4th and 5th Jana Steiger and Ken Sauer, 8th Mel Klein, 10th Heinz Frei, right extreme Ignacio Tinoco. *Fourth row*: from left, 1st and 2nd Warren Beck and Vittal Yachandra, 5th and 7th Holger Dau, Martin Debreczeny, extreme right Melissa Grush, Gary Smith and Wen Liang, *Last row*: extreme right Enrique Dalmasso and Matthew Latimer. Photo courtesy of Vittal Yachandra.

**Fig. 6:**
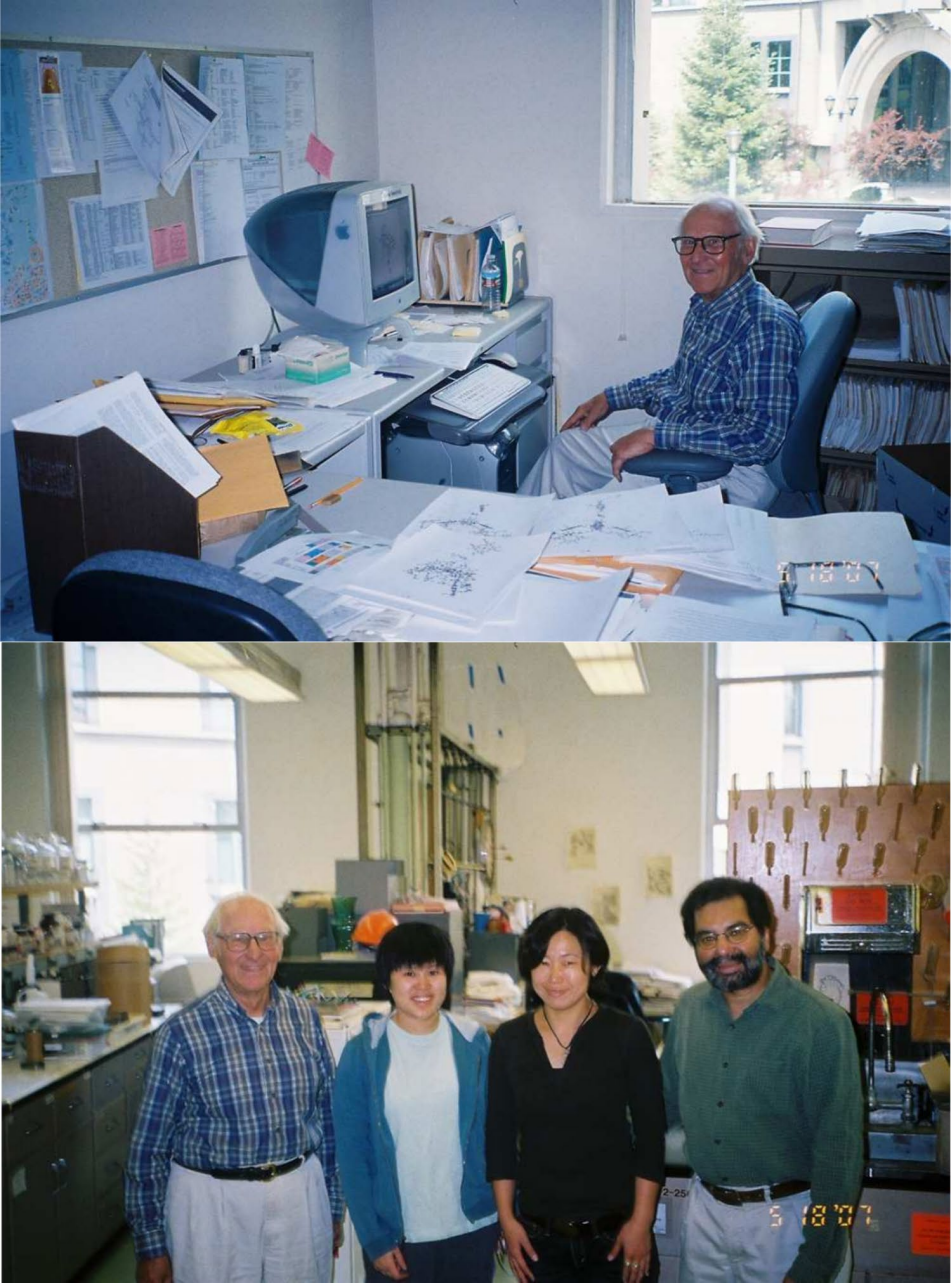
*Top*: Ken in his office in the Calvin Lab 2007. *Bottom*: In the Calvin Lab in front of one of the ‘spokes’ in the circular building. Picture taken just before moving from the Calvin Lab to building 66 on the hill site of LBNL in 2007. From Left: Ken Sauer, Salina Long, Junko Yano, Vittal Yachandra. Photos courtesy of Vittal Yachandra

The round Calvin Lab building as designed by Calvin, had no separate divisions for students and postdocs from various principal investigators. Each one had one ‘spoke’ in this circular building which was their lab bench (Fig. 6). So, everyone was together, and worked together. If one wanted to learn about other topics, they were just right next or around you. In the Calvin Lab, one could easily interact with scientists with diverse skills, from people applying molecular biology to photosynthesis research, which was a new set of techniques in the early 1980s. Ken really was one of the major people (along with Mel Klein) who encouraged this kind of interdisciplinary work and we all thrived in this unique atmosphere, and learned a whole lot from our close interactions with Ken and others.

## Scientific accomplishments

Research in Ken’s group followed several major themes, with the common thread being the use of physical and chemical techniques, including numerous types of spectroscopy, to understand the molecular aspects of biological systems, primarily photosynthetic in nature. Techniques utilized included: circular dichroism and magnetic circular dichroism, electron paramagnetic resonance, ultrafast fluorescence and absorption, and most importantly, X-ray spectroscopy of the Mn center in Photosystem II.

**Fig. 7:**
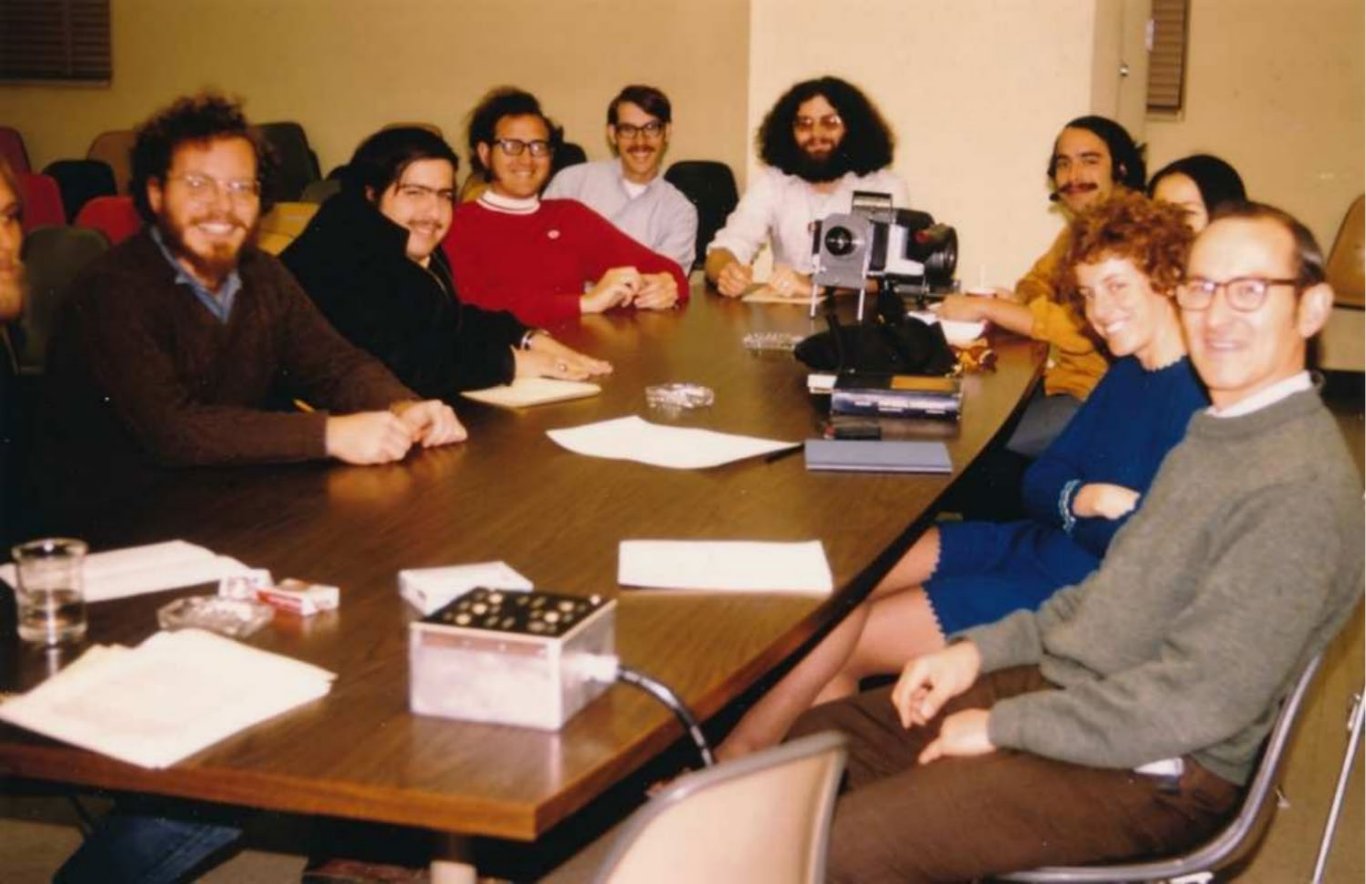
Sauer group meeting in 1971 in the Calvin Lab seminar room, during a seminar by Paul Mathis. From left, Jerry Babcock (largely cut off), Jim Ellenson, Bruce Henkin, Doug Vaughan, Art Ley, Bob Blankenship, Ken Philipson, Vicki Sato, Ora Canaanai, Ken Sauer. Photo courtesy of Paul Mathis

Figure 7 shows a typical Sauer group meeting in the 1970s in the laid back relaxed style of Berkeley in those days.

**Fig. 8:**
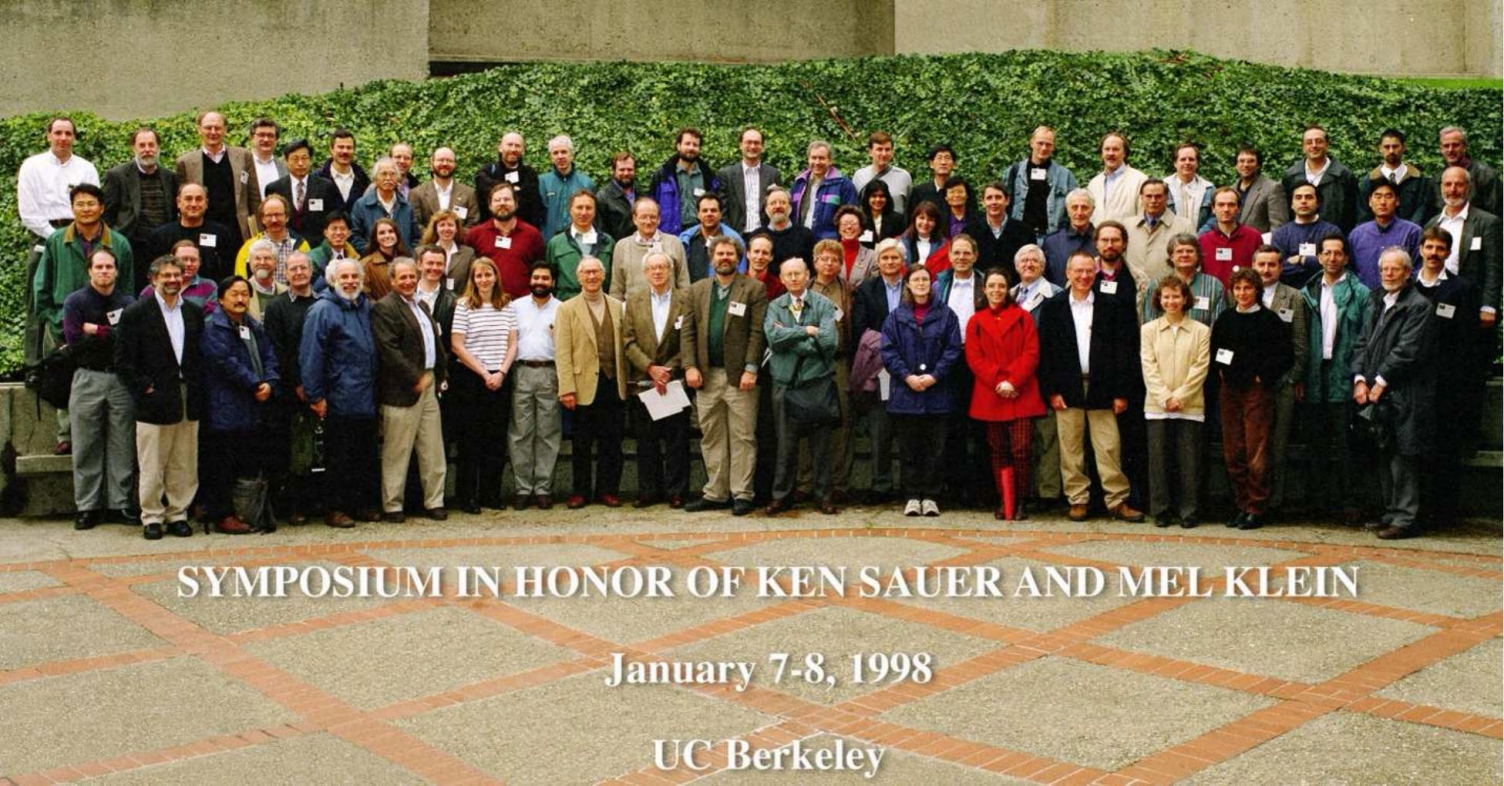
Symposium in Honor of Ken Sauer and Mel Klein in UC Berkeley, in January 1998. Alumni, students, postdocs, collaborators, visitors, and colleagues. *Top Row*: Henk Visser, Graham Fleming, Jerry Babcock, John Golbeck, Tsunenori Nozawa, Gary Brudvig, Ning Pon, Ken’s sons—Terry Sauer, Bob Sauer, Bill Rutherford, Harry Frank, Mike Boska, Tom Pratum, John McCracken, Alan Koretsky, John Robblee, Joon Woo Park, Uwe Bergmann, Steve Cramer, Stan Sorscher, Greg Karczmar, Reza Yewari, Shavel Dave, Ken’s brother-in-law – Art Limbird. *Middle Row*: Yeon-Kyun Shin, Alex Pines, David Wemmer, Roehl Cinco, Jana Steiger, Annette Rompel, Olaf Burghaus, Tasios Melis, Micha Tomkeiwicz, David Goodin, John Markley, Kerry Karukstis, Ishita Mukerji, Vickie DeRose, Ms. Joon Woo Park, Jean-Jacques Girerd, Erwin Hahn, John Waugh, Johannes Messinger, Emmanuele Bellachio, Inmong Choi, Wolfgang Haehnel, *Front Row*: Lane Gilchrist, Rich Friesner, David Britt, Sun Un, Ron Guiles, Hugo Scheer, Hyman Hartman, John Hearst, Steve Worland, Ann McDermott, Vittal Yachandra, Ken Sauer, Mel Klein, Bob Blankenship, Chuck Dismukes, Paul Mathis, Don Bryant, Wolfgang Lubitz, Mary Talbot, Dietmar Stehlik, Elodie Anxolabéhere-Mallart, Ed Dratz, Tom Wydrzynski, Holger Dau, Shelly Pizarro, Gennady Ananyev, Carmen Fernandez, Alfred Holzwarth, Jim Cole, Klaus Mobius, Rob Burnap. Photo courtesy of LBNL Photo Archives

As pointed out by Jerry Babcock in an Introduction he wrote for a Festschrift in J. Phys. Chem. B 1998 (celebrating the career of Ken Sauer and Mel Klein), one of the hallmarks of Ken’s long career in science was his ability to identify and apply useful new physical methods to challenging biological problems (Babcock 1998). The alumni, sabbatical visitors, and other visitors to the lab, collaborators, and colleagues, and then current graduate students, postdocs all came together in 1998 to the Calvin Lab and UC Berkeley to celebrate the combined careers of Ken Sauer and Mel Klein (Fig. 8). It was indeed a joyous occasion with many alumni giving talks, presenting posters, followed by a memorable dinner in the UC Berkeley Faculty Club with a great many fond stories of their times in Berkeley.

We have tried to capture a profile of his research, which is by no means easy. In his early career, Ken used optical rotatory dispersion (Sauer 1965) followed by circular dichroism (Sauer et al. 1968) to study the organization of chlorophyll in photosynthesis, which eventually lead to the realization of the dimeric chlorophyll structure in the reaction centers of higher-plants (Philipson and Sauer 1972); a pioneering application of CD indeed. Another early major accomplishment of Ken and his group, especially Bob Blankenship and Jerry Babcock, was in identifying the immediate electron donor to the Mn complex, later unequivocally identified as tyrosine fittingly by Babcock’s group (Babcock et al. 1976; Blankenship et al. 1975b; Warden et al. 1976). The presence of an intermediate between the redox catalyst and the reaction center where the photochemistry occurs has had a profound influence on the understanding and also design of artificial photo-induced water-splitting catalyst. Ken used magnetic circular dichroism to study metalloproteins (Vickery et al. 1975; Vickery et al. 1976b, a), and showed how to use resonance Raman to exploit charge-transfer transitions in metallo-porphyrins (Asher and Sauer 1976; Asher et al. 1977), discovered the spin-polarized EPR signal in Photosystem I (Blankenship et al. 1975a; Friesner et al. 1979), and developed the use of picosecond time-resolved single-photon counting fluorescence methods (Hartig et al. 1976, 1977; Leskovar et al. 1976; Reisberg et al. 1982) to understand energy transfer in antenna complexes and phycobilisomes (Beck and Sauer 1992; Debreczeny et al. 1995b, a; Freer et al. 1996; Gindt et al. 1992; Glazer et al. 1994; Karukstis and Sauer 1983b; Maxson et al. 1989; Mukerji and Sauer 1993; Pizarro and Sauer 2001; Sauer et al. 1996), and electric field effects to study charge separation in reaction centers (Dau and Sauer 1991, 1992; Steiger and Sauer 1995). During a sabbatical with Paul Mathis, in Saclay, Ken made the initial time-resolved observations on forward and reverse electron transfer in the Photosystem I reaction center (Mathis et al. 1978; Sauer et al. 1978, 1979) that have sparked considerable activity in a number of labs. Shortly thereafter, he also initiated theoretical considerations of partially oriented systems that continue to be of fundamental importance (Friesner et al. 1980; Nairn et al. 1981).

**Fig. 9:**
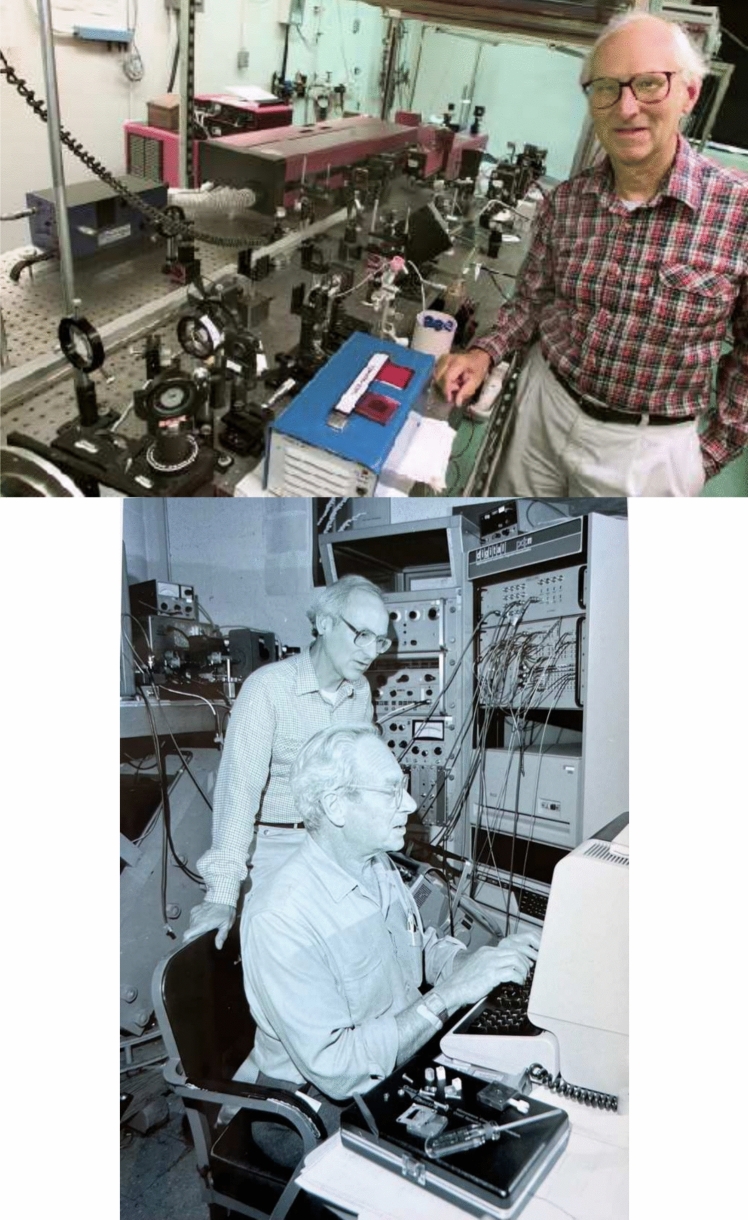
*Top*: Ken ~ 1998 in the laser lab where much of the time-resolved fluorescence lifetime measurements of antenna complexes were collected. Photo courtesy of LBNL photo archives. *Bottom*: Ken with Mel Klein ~ 1990s in the EPR lab. Photo courtesy of Vittal Yachandra

In the early 1980’s, Ken joined forces with his long-time colleagues Mel Klein (Fig. 9) and Vittal Yachandra (from 1982) and, subsequently, with Junko Yano (from 2001) and Jan Kern (from 2008). Together, they employed X-ray absorption spectroscopy for investigating the manganese complex at the heart of the photosynthetic water-splitting complex. This revealed important results about the geometric and electronic structure of the catalyst and the changes that it went through during the Kok cycle that were published in a series of papers over a period of more than a two decades. A few representative papers covering the wide range of results and the people from the group who participated in these studies are referred to here (Cinco et al. 1998, 1999, 2002; Cole et al. 1987; Dau et al. 1995; Derose et al. 1994, 1995; Goodin et al. 1984; Guiles et al. 1990a, 1990b; Latimer et al. 1995, 1998; Liang et al. 1994, 2000; McDermott et al. 1988, 1989; Messinger et al. 2001; Mukerji et al. 1994; Pizarro et al. 2004; Pushkar et al. 2008; Robblee et al. 2002; Roelofs et al. 1996; Rompel et al. 1997; Visser et al. 2001; Yachandra et al. 1986, 1987, 1993; Yano et al. 2005a, 2005b, 2006). The results are summarized in many reviews (Sauer and Yachandra 2004; Sauer et al. 2005, 2008), and in this highly cited review (Yachandra et al. 1996). This work was indeed a tour de force of interdisciplinary research that formed the basis for revealing the structure of the Mn complex and its changes as it advances through the S-state cycle. This research line reached a watershed recently with the use of XFELs for collecting room temperature X-ray diffraction and spectroscopy by his colleagues Vittal Yachandra, Junko Yano, Johannes Messinger and Jan Kern (all postdocs of Ken Sauer), with the publication of high-resolutions structural models of Photosystem II and its Mn_4_CaO_5_ complex as it traverses the S-state cycle of water oxidation. Ken followed this XFEL work avidly and provided encouragement as always (Kern et al. 2013, 2018; Bhowmick et al. 2023).

In his career, in addition to earlier work on magnetic resonance on triplet states (Frank et al. 1979, 1980), and the spin-polarized time-resolved experiments (Friesner et al. 1979; McCracken et al. 1982; McCracken and Sauer 1983), Ken used EPR extensively along with Mel Klein to study the manganese cluster; extending the pioneering work of Blankenship and Babcock on the signal II kinetics (Boska et al. 1983; Boska and Sauer 1984), co-discovering the g = 4.1 EPR signal in the S_2_ state (Casey and Sauer 1984), the parallel polarized EPR signal from the S_1_ state (Dexheimer et al. 1989), performed extensive studies of the S_2_ state multiline EPR signal (MLS) (discovered by Chuck Dismukes at Princeton, who was earlier a postdoc at Berkeley with Ken) with Gary Brudvig (Brudvig et al. 1983), histidine ligation and NH_3_ modified signals using EPR and ESEEM methods with David Britt and Vickie DeRose (Britt et al. 1989; Derose et al. 1991; Kim et al. 1992) (and XAS of NH_3_ modified PS II with Holger Dau (Dau et al. 1995)), the S_0_ MLS, similar to the S_2_ MLS signal, with Johannes Messinger (Messinger et al. 1997); these postdoctoral fellows have gone on to become leaders in the field of photosynthesis in their own careers. As with the fluorescence and X-ray absorption work, the work on magnetic resonance in Photosystem II continued on in Berkeley after his retirement with important, new studies such as single crystal EPR (Yano et al. 2004). Throughout his research career, Ken continued to develop and apply new techniques that contributed to providing new insights.

## Teaching

Ken loved to teach; he was really good at it, and he took teaching very seriously. If you went camping or went to Point Reyes or conferences with Ken, you often ran into someone who knew Ken and who had taken his courses at UC Berkeley, showing what a huge legacy Ken had by educating thousands of undergraduate students, in addition to his graduate students and postdoctoral researchers (see SI). Public speaking and writing well were both equally important to Ken and he was also very serious about teaching students on how to speak in public and how to present their data. He believed that just collecting data was not enough; you have to transmit it. He also taught how to write clearly. One frequently heard from his students and postdocs about how nervous they were to give a talk before him, and at the same time, how much they learned from him on how to present their data well both verbally and in written form.

His review articles, written clearly in simple understandable language, setting forth the state of the knowledge, and pointing out the future course have served many a starting researcher well. Ken’s reviews provided a rich resource for beginning graduate students and established researchers in the field of photosynthesis (Sauer and Yachandra 2004; Sauer 1978, 1979, 1980, 1995; Sauer et al. 1992, 2008; Karukstis and Sauer 1983a; Klein et al. 1993; Yachandra et al. 1996). Ken’s remarkably prescient review in Accounts of Chemical Research in 1980 (Sauer 1980) on the topic of the role manganese in the water oxidation and oxygen evolution and also its importance for renewable energy, when renewable energy was still not yet in fashion, set the stage for much of the later research in his group on the use of X-rays to understand this reaction mechanism which continues to this day at LBNL.

Ken’s knowledge and foresight continues to be imparted through a textbook he co-wrote, first published in 1978, entitled *Physical Chemistry: Principles and Applications in Biological Sciences*. After five editions, it is a mainstay in many Biophysics and Physical Chemistry courses today and a trusted resource for researchers.

**Fig. 10:**
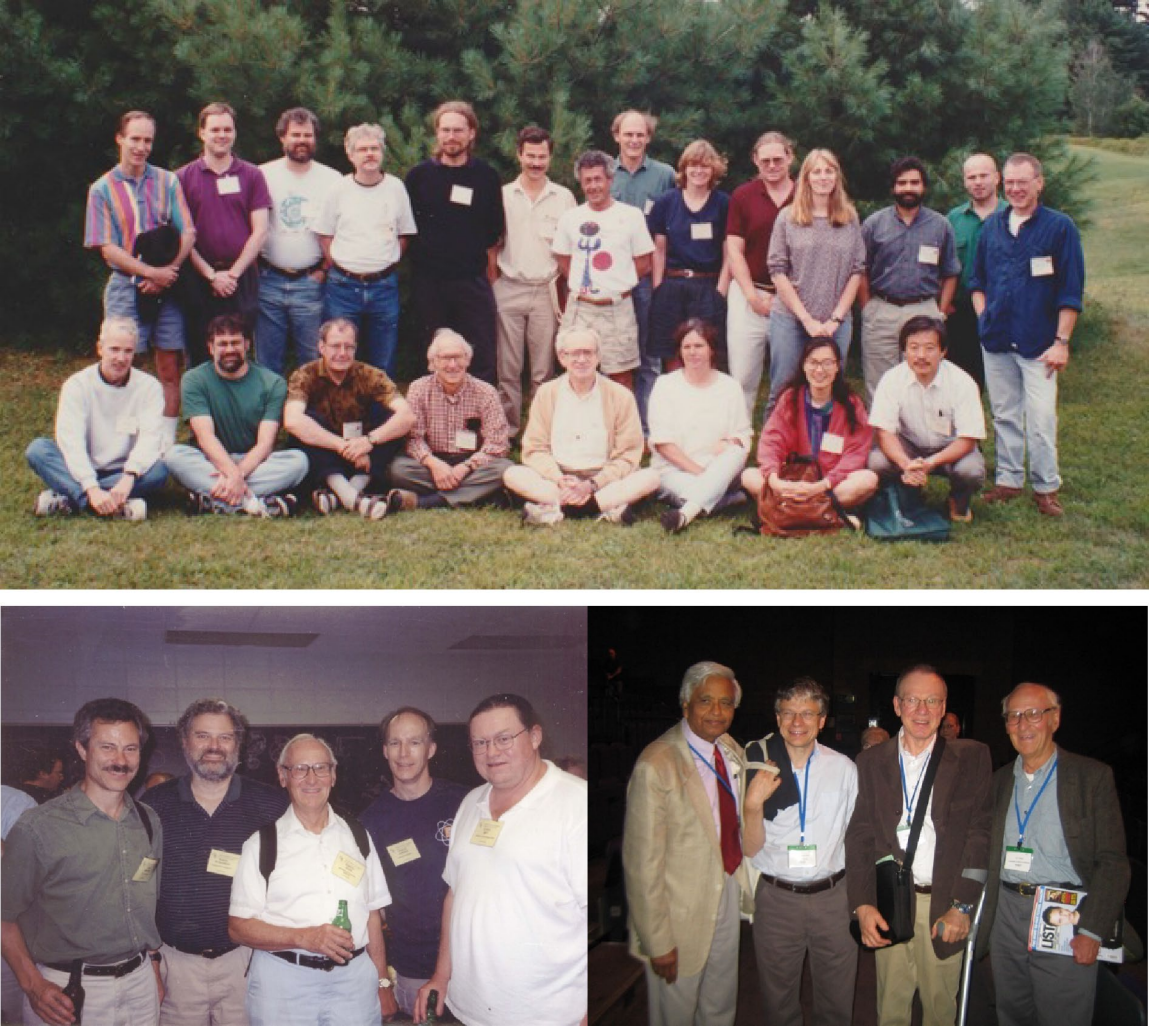
*Top*: Photo of students, postdocs and visitors of the Sauer group taken at the 1994 Gordon Research Conference on Photosynthesis. *Sitting row (from left)*: Harry Frank, Rich Friesner, Hugo Scheer, Ken Sauer, Mel Klein, unidentified, Wen Liang, Masami Kusunoki. *Standing row*: Chuck Dismukes, Warren Beck, Bob Blankenship, Joe Warden, Holger Dau, Gary Brudvig, Jacques Breton, Jerry Babcock, Yvonne Gindt, Dave Britt, Ann McDermott, Vittal Yachandra, Theo Roelofs, Tom Wydryzinski. Photo courtesy of Bob Blankenship. *Bottom Left*: At the 2003 GRC in Photosynthesis with favorite beverages. From left: Gary Brudvig, Bob Blankenship, Ken Sauer, Chuck Dismukes, David Britt. Photo courtesy of Vittal Yachandra. *Bottom Right*: At the International Photosynthesis Congress in Glasgow, 2007, From left: Govindjee Govindjee, Julian Eaton-Rye, Tom Wydrzynski, Ken Sauer. Photos courtesy of Govindjee Govindjee

Ken went on numerous trips for Gordon conferences and other international conferences and invited talks and awards (Fig. 10).

## Extra-curricular activities

**Fig. 11:**
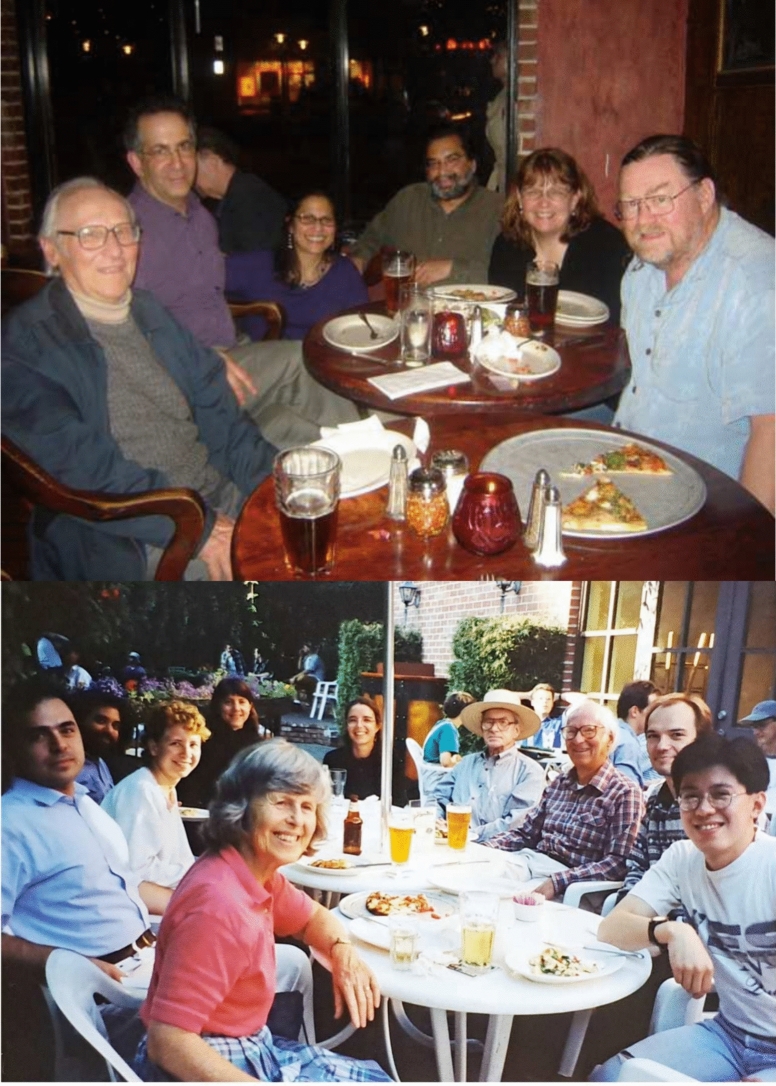
*Top*: Alumni from the mid 1980s to early 90 s in Jupiter. Ken, Jim Cole, Ishita Mukerji, Vittal, Vickie DeRose, Dave Britt. Photo courtesy of Vittal Yachandra. *Bottom*: Sauer group excursion to Jupiter brewery, about 1997. Clockwise from left, Margie Sauer, Emmanuele Bellacchio, Shelly Pizarro, Vittal Yachandra, Karen McFarlane-Holman, Elodie Anxolabéhere-Mallart, Melvin Klein; Kenneth Sauer; Johannes Messinger, Roehl Cinco. Photo courtesy of Carmen Fernandez

In addition to scientific research, the group was a congenial and friendly place that had a sense of camaraderie, whether in the lab, or during data collection at X-ray sources, or at conferences. The group often went out for dinners and got together for relaxation over drinks and pizza. Figure 11 shows two examples of such group excursions to the group’s favorite beer gardens in Berkeley.

**Fig. 12:**
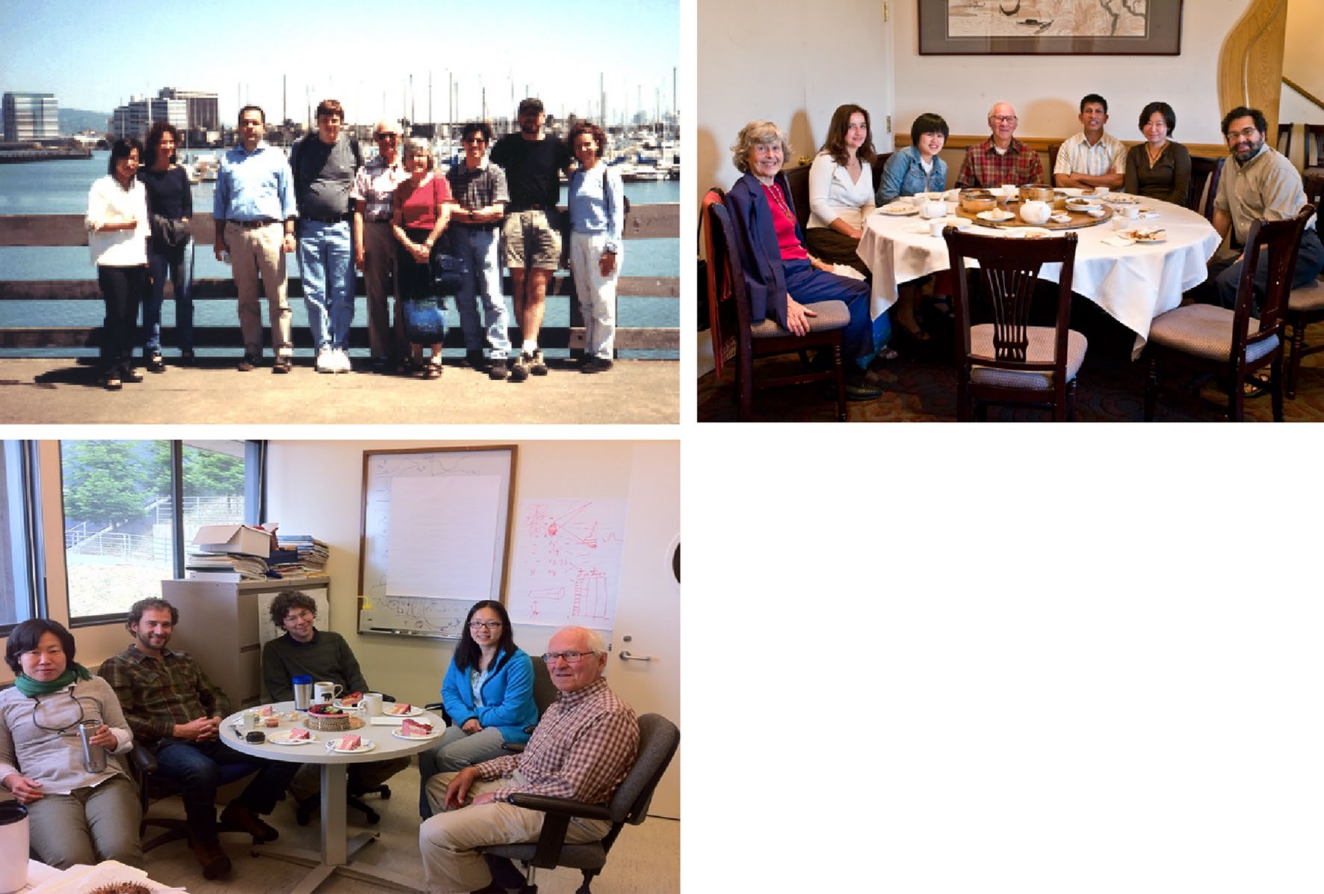
*Top Left*: Ken’s 70th birthday on the Emeryville Marina, 2001. From left: Junko Yano, Azul Lewis, Emmanuele Bellachio, John Robblee, Ken, Margie, Roehl Cinco, Henk Visser, Shelly Pizarro. *Top Right*: Ken’s 75th birthday celebration (2006) with dim sum. From left: Margie Sauer, Yulia Pushkar, Salina Long, Ken Sauer, Sudip Mukophadhyay, Junko Yano, Vittal Yachandra. *Bottom Left*: Ken’s 80th birthday cake (2011) in building 66 on the hill campus of LBNL, From left: Junko Yano, Ben Lassalle, Jan Kern, Rosalie Tran, and Ken. Photos courtesy Vittal Yachandra

It was always a tradition to celebrate birthdays in the lab and here are a few pictures of celebrating some landmark birthdays of Ken when he turned 70, 75 and 80 (Fig. 12).

**Fig. 13:**
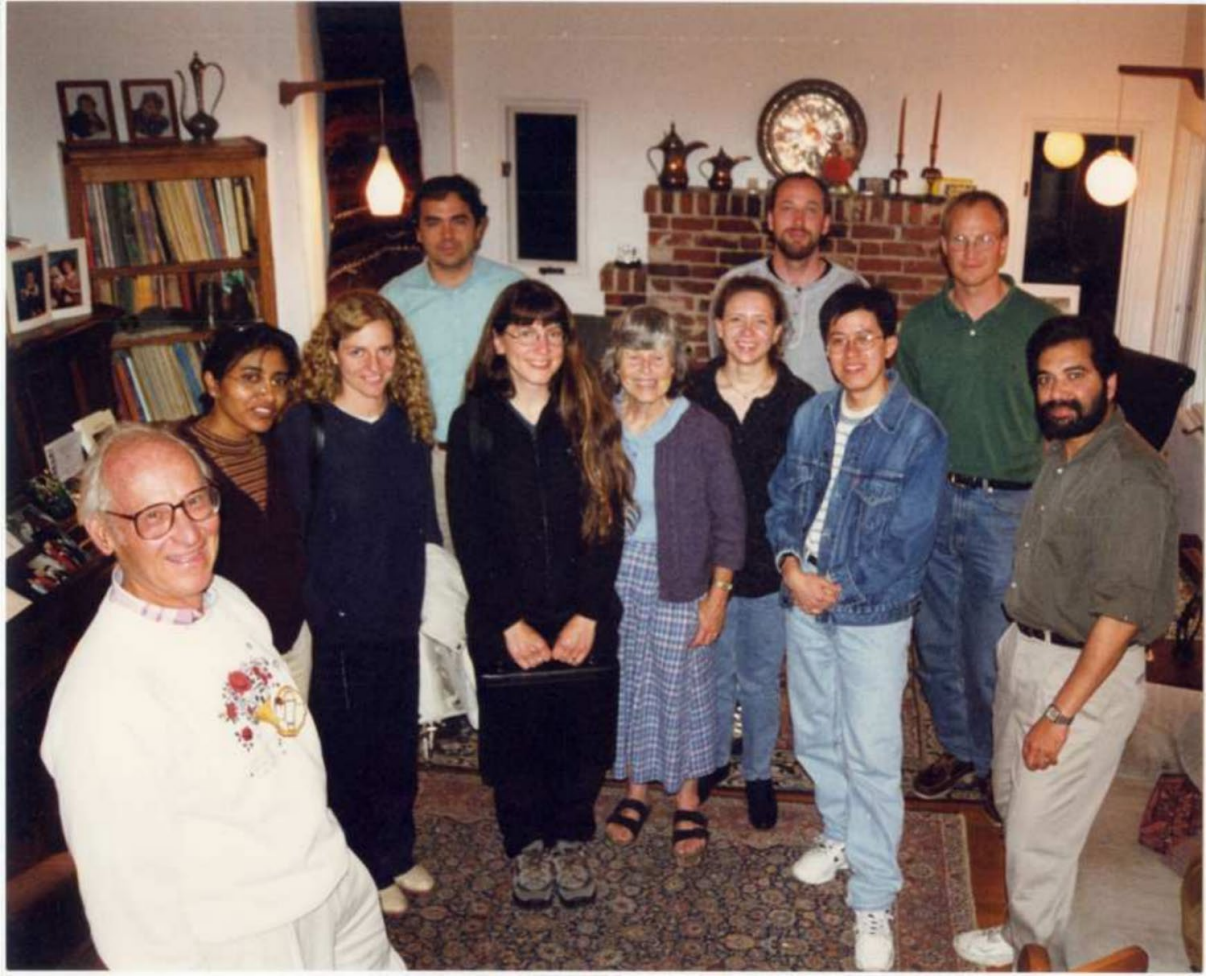
One of many group get togethers in Ken and Margie’s charming house in the Berkeley hills in 2000. From left Ken, Vinita Singh, Carmen Fernandez, Emanuele Bellachio, Karen McFarlane, Margie Sauer, Shelly Pizarro, Henk Visser, Roehl Cinco, Uwe Bergmann, Vittal Yachandra. Photo courtesy of Vittal Yachandra

**Fig. 14:**
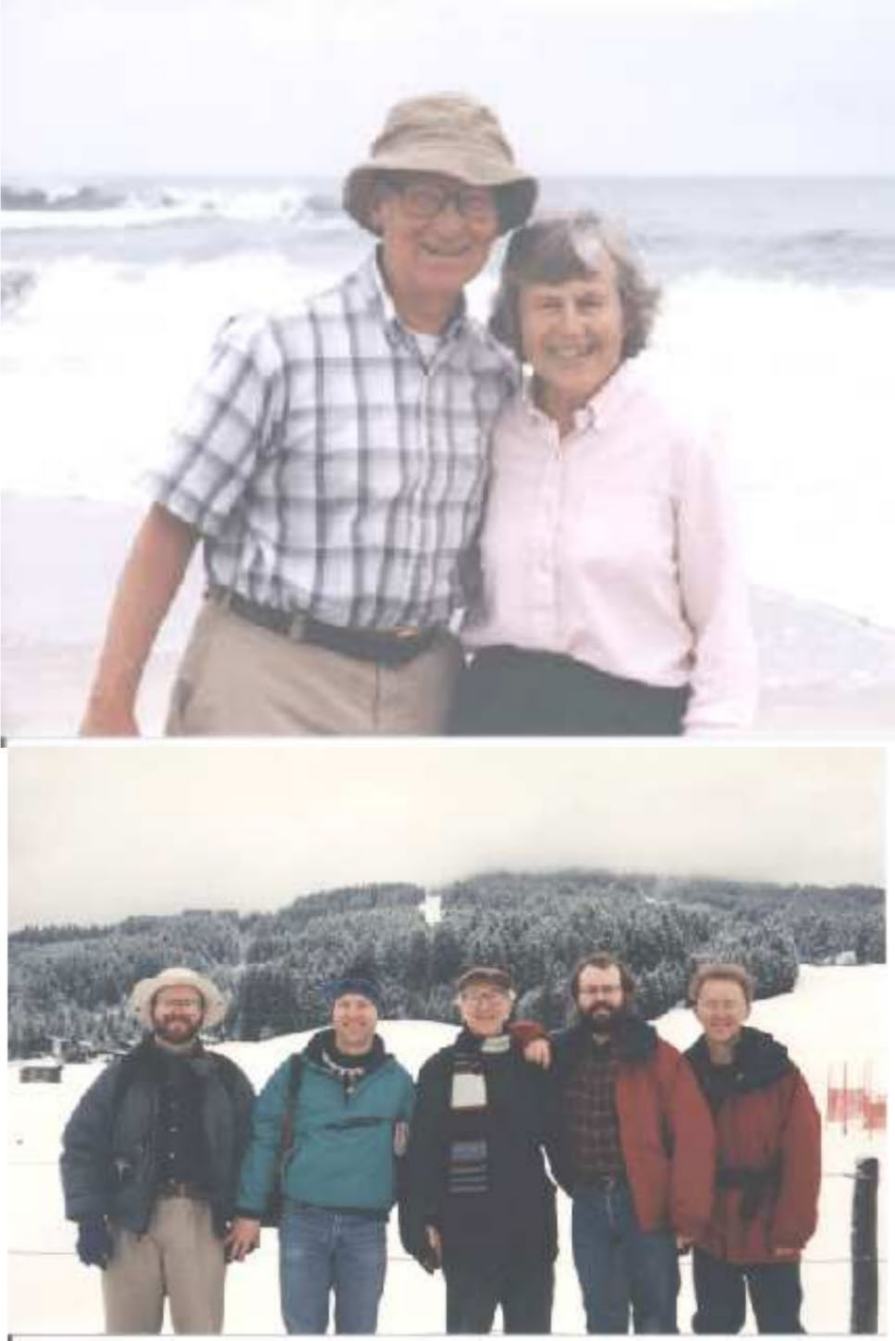
Ken and Margie were avid outdoors people and were constantly going on trips with family and also inviting the group. *Top*: Ken and Margie probably on the pacific coast at Asilomar, where the Western Photosynthesis Conference was held for many years and that Ken and Margie always attended. *Bottom*: Ken and Margie on a trip to the Austrian Alps with sons Bob, Terry. Ken Rodney and Peter. Photos Courtesy of Margie Sauer

There were also regular group dinners at the Sauer’s comfortable home (Fig. 13), and numerous celebrations of many group events such as graduations, weddings, baby showers, and other group accomplishments, where Margie often served delicious middle eastern food. These events often also included raspberry picking in their charming backyard garden followed by home-made ice cream (see reminiscences from YYK below). Ken and Margie were avid outdoor enthusiasts (Fig. 14), and they went on numerous picnics, bike rides, as well as camping, skiing and canoe trips. The research group was frequently invited along on these excursions.

## Reminiscences of colleagues

The reminiscences appear below mostly in alphabetical order, except the contributions from the authors of this tribute, Johannes Messinger, Bob Blankenship, Junko Yano, Vittal Yachandra and Jan Kern, which are collected at the end.

## Uwe Bergmann

### University of Wisconsin

Ken was an outstanding choir singer with a beautiful deep voluminous baritone voice. My wife Elisabetta and I went to numerous concerts of the San Francisco Bach Choir where Ken and Margie sang. I vividly remember the wonderful performance of the complete Bach Christmas Oratorio in Palo Alto and a stunningly beautiful baroque candlelight concert performance in San Francisco.

Ken was also an avid and passionate swimmer. Ken typically walked from the Calvin Lab around the Memorial Stadium up the hill on Strawberry Canyon Road. My swim group and I came down by car from LBNL, so we frequently met either at the parking lot of the Strawberry Canyon Pool, or sometimes in the water. During these encounters we usually avoided talking about research but rather appreciated how lucky we are to have this wonderful passion and the freedom to pursue it.

## R. David Britt

### University of California, Davis

I have had a long career in the UC Davis Chemistry Department, but students are often surprised to learn that I have no chemistry degrees or formal chemistry training. Working in Mel Klein’s group, closely associated with Ken Sauer, in the interdisciplinary Melvin Calvin Laboratory for Chemical Biodynamics, was crucial for this transition from an education in “straight physics” to my becoming an academic chemist (of sorts). Mel never would have considered himself a chemist: he was a unique blend of engineer (his educational background) and experimental biophysicist. Ken of course was a classically trained physical organic chemist and a faculty member in the great Berkeley Chemistry department. Ken and Mel’s group meetings were joint during my time in the lab, so it was a fantastic opportunity to learn how Ken thought about the issues of the day, bringing a real chemist’s perspective to photosynthesis, which was a rapidly evolving field in the 80 s. Moreover, Ken ran a lab with many talented Berkeley Chemistry students and postdocs that I could learn from….folks who have greatly populated the photosynthesis community. There were also often great sabbatical visitors to learn from. Ken was always happy to discuss topics with me, as one of Mel’s physics students who needed to improve their knowledge of chemistry, and to send me off for some rudimentary readings on topics like chemical thermodynamics and biochemistry. So I would say a very short answer to the “how did you become a chemistry professor?” is *Ken Sauer*.

## Douglas Bruce

### Brock University

I first met Ken when I was a young undergraduate student in Vancouver at Simon Fraser University in the late 1970’s. Our biophysics course went on a field trip to the rival university, the University of British Columbia, to listen to a talk that had been organized by Beverly Green. She had invited Ken to give a seminar on his work on photosynthesis and our professor, Konrad Colbow, a physicist working in photosynthesis research brought us along to hear him. This was the first research seminar I had attended and it was absolutely fascinating to listen to Ken talk about processes in photosynthesis as studied by ultrafast time scale spectroscopy. Details of the talk fade with time, but Ken’s talent for speaking and enthusiasm for the material are still crystal clear in my memory. One of the strongest memories of that day was Ken’s voice. His surprisingly resonant bass voice was as mesmerizing as the experiments he was talking about. A few years later, as a graduate student with Bill Vidaver and Konrad Colbow at SFU I became more familiar with Ken’s stature in the field of photosynthesis and his efforts to push our understanding of faster and faster processes of primary energy conversion in photosynthesis. I wasn’t to meet Ken again in person until my postdoctoral years spent with John Biggins at Brown University. John took me to my first Gordon conferences where I met many of my photosynthesis research heroes for the first time, including Ken Sauer. John and Ken were great friends and had worked together in the Calvin lab in the early 1960’s. What quickly became apparent to me during these conferences was the concept of photosynthesis research “families” made of younger researchers who had been graduate students or postdocs of more established researchers. I found it absolutely amazing how many of the most influential researchers making strong progress in biophysical approaches to photosynthesis had come from the Sauer lab. Ken led by his example of enthusiasm for research, mentoring and fostering creativity and collaboration. The contributions that Ken made to the training and success of so many researchers is truly one of his greatest legacy’s.

## Gary Brudvig

### Yale University

I had the opportunity to work with Ken Sauer as a postdoc for two years from 1980 to 1982. I arrived at Berkely with no background or experience at all in the field of photosynthesis, having completed my Ph.D. research studying the metal centers in cytochrome *c* oxidase. Nonetheless, Ken welcomed me into his research group and gave me freedom to explore. I was interested to study the oxygen evolving complex (OEC) of photosystem II and began using EPR spectroscopy to probe the S-state intermediates. After many unsuccessful attempts to generate flash-induced EPR signals from the OEC in spinach chloroplast membranes using a monstrous home-built bank of flash lamps that was constructed before my time in the lab, we were finally successful to generate S_2_-state EPR signals using continuous illumination at 200 K (Brudvig et al. (1983) *Biochim. Biophys. Acta* 723, 366–371), a result that proved very helpful when I started my independent position at Yale University.

I was just one of many who worked with Ken Sauer. Indeed, one of Ken’s greatest contributions to the field was his phenomenal record of training students and postdocs. This was perhaps most evident after Ken finished his Thursday night talk at a Photosynthesis Gordon Research Conference when the session chair asked everyone who had worked with Ken to stand up (see Fig. 10). A good fraction of the audience rose to their feet. Although Ken Sauer’s passing is a sad conclusion to a tremendous career, his legacy lives on with the many researchers in the field of photosynthesis who trained in the Sauer group and continue to push forward the field of photosynthesis.

## Donald Bryant

### Pennsylvania State University

In the late 1980’s and early 1990’s, my research group enjoyed a productive collaboration with Ken concerning energy transfer dynamics in phycocyanin and the functional roles of allophycocyanin variants in phycobilisomes in cyanobacteria. Ken was a wonderful collaborator and was very supportive of me as a young scientist and professor. I regarded him as an important advisor and mentor, and I enjoyed and valued our face-to-face interactions most of all when I visited Berkeley. I was very happy to contribute a paper to the tribute issue on far-red light absorbing allophycocyanins that I feel Ken would have enjoyed very much. Ken may have been small in stature, but he had an enormous impact on photosynthesis research over the past 50 years. I am proud to have benefited from that impact.

## Rob Burnap

### Oklahoma State University

I might mention in this context that Ken was perhaps the first true photosynthesis person I really encountered who took some time with me. It was at the International Congress in Brussels that I had somehow won a travel scholarship to present my Ph.D. work and Ken was in a different poster section where he had a small group of students (me joining in) and he was joyfully explaining his thoughts, if memory serves, how excitons were thermally excited to move energetically uphill and so on. Anyway, I thought it definitely sounded cool and I think that was really when I decided that this would be the community that I wanted to work in.

## Ruchira Chatterjee

### Amgen

I had the profound honor of being around Prof. Kenneth Sauer (Ken to us) when I joined Lawrence Berkeley National Lab as a Postdoctoral fellow in 2013. Ken was a true luminary, his fascinating anecdotes were not just tales of scientific achievement but reflections of a lifelong dedication to discovery and a profound love for the field of Photosynthesis. His groundbreaking research, innovative methods, and influential publications have shaped the field and inspired generations of biophysicists/physical chemists to follow his footsteps. His enthusiasm during our discussions over lunch was infectious, and his care for his past students was palpable. The legacy Ken leaves behind is immeasurable. His wisdom will continue to influence and guide us as we carry forward the torch of biophysics, driven by the same curiosity and dedication he so perfectly embodied. Ken’s contributions and his impact on our lives are enduring, and for that, we are forever grateful.

## Richard Cogdell

### University of Glasgow

I first met Ken in the 1970’s when he invited me to come and give a seminar at Berkeley. This was important for me as it was the start of a long friendship with Ken and Margie as well as the first time I really met Harry Frank and began a career long collaboration with him on carotenoids.

Subsequently, I was delighted in the 1990’s when Ken came to Glasgow for one summer to work with Neil Issacs and myself. We had just finished determining the structure of a purple bacterial LH2 complex and Ken wanted to try to use the information in the structure to calculate its absorption spectrum. He was successful in this endeavour, and we produced a nice paper describing this that has subsequently been well cited (Sauer et al. (1996) ‘Structure-based calculation of the optical spectra of the LH2 bacteriochlorophyll-protein complex from *Rhodopsuedomonas acidophila*’ Photochem. Photobiol. 64, 564–576). This was a very important piece of work for me as Ken was able to take me systematically, and indeed quantitatively, through the various factors that influenced where the Q_y_ bands of the Bacteriochlorophylls were in LH2 compared with where the Q_y_ band was located for monomeric bacteriochlorophyll in organic solvents. Not for the last time, Ken taught me an important lesson. It was fun that summer to really get to know Ken properly. All in all, that was very interesting time and, in many ways, also rather unique. Glasgow has a bad reputation for poor weather. But that summer the weather was magnificent and, on several days, Ken had to vacate his office early in the afternoon as the sun made it too hot! We often hoped that Ken would come back again and bring that California weather with him!

During this time, I was also lucky enough to work in Munich with Hugo Scheer. Hugo and Ken were good friends too and, on several occasions, we spent very happy periods together in Munich. Hugo organised an influential series of conferences on reaction centres and light harvesting in Freising to which both Ken and I were regular attendees. I have lovely memories of both Ken and Margie in Munich, as illustrated by the picture below. I found Ken to be very supportive and an excellent role model. He was great to discuss your latest experiments with. He never ever said you should do this, or you should do that, but if you had eyes to see, then he provided a clear picture of how to value, encourage and support younger colleagues that I have always tried to emulate. He was not only a first-class scientist but also a highly valued friend.

**Figure:**
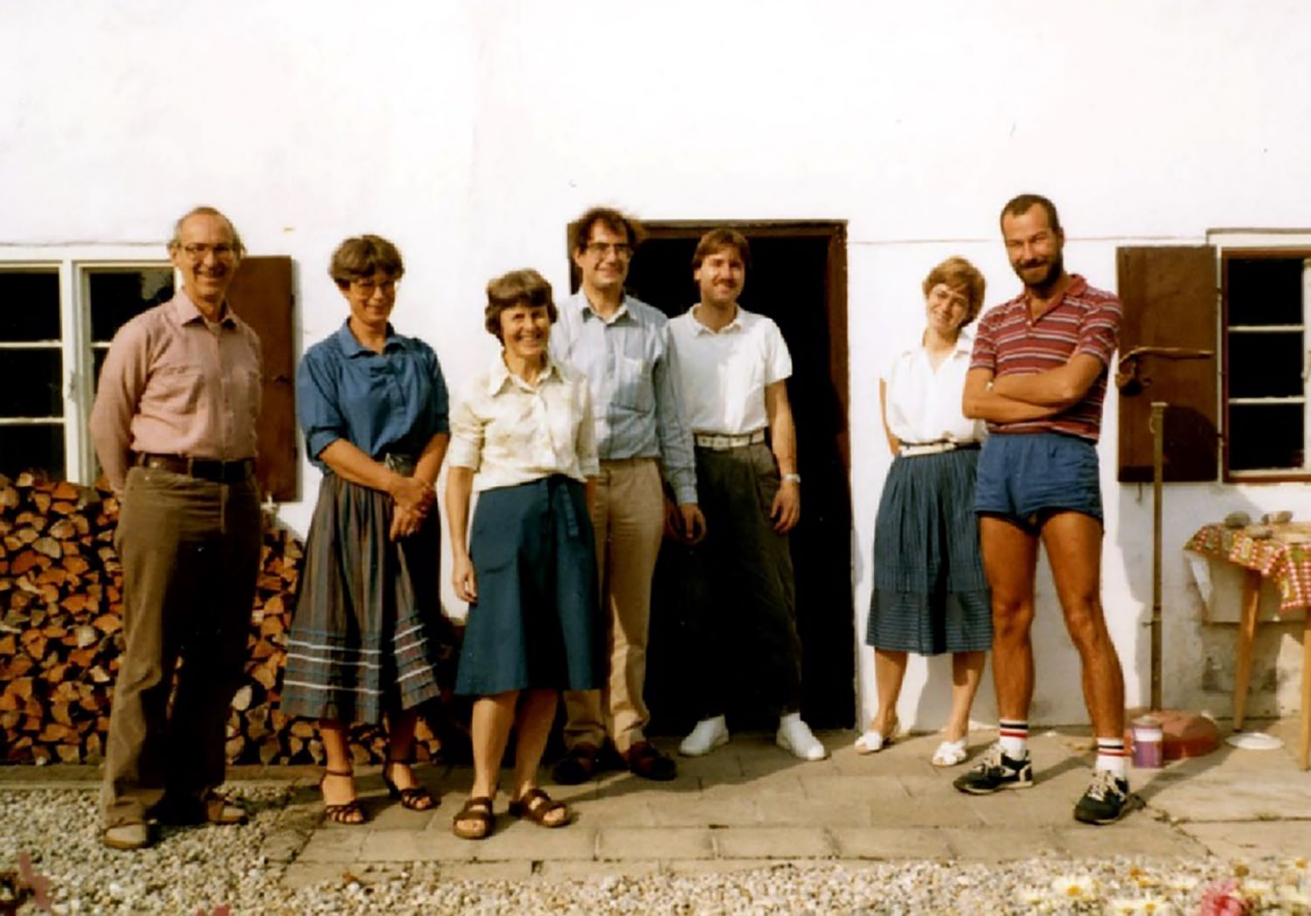
A picture taken at Hugo Scheer’s country farmhouse outside of Munich. From left to right. Ken Sauer, Renate Scheer, Margie Sauer, Hugo Scheer and then members of Hugo’s group. Photo courtesy of Richard Cogdell

## Holger Dau

### Free University Berlin

The day in the summer of 1990 when we came to Berkeley as a family with two small children is one of my treasured memories. Ken and his wife Margie had not only organized the rental of a nice and really inexpensive furnished apartment where we lived happily for 2 ½ years, but had also stocked the apartment with dishes and a huge basket of California fruit. In the Calvin lab, Ken introduced me to the group, discussed with me how I could get started, and then I had all the freedom in the world to do my research on the mechanisms of variable photosystem II fluorescence. From time to time, we would meet on the floors of the round lab, and Ken would usually suggest interesting hiking opportunities in California or other recreational activities in the Bay Area. These were entertaining conversations with the humorous and always smiling Ken. At some point, however, I began to wonder if Ken really cared about my scientific work. But he did. Whenever I actively sought a discussion about my progress, Ken was on hand, gently giving me expert advice and offering to help me make useful connections with other photosynthesis researchers. I really enjoyed Ken's style of laissez-faire guidance. And later I tried to develop a similar relaxed leadership style in my own group, but I could never do it as well as Ken.

## Vickie DeRose

### University of Oregon

Reflecting on Ken, these words come to mind: Solid, steadfast, stentorian. High standards. Detail-oriented. A sense of humor. As I edit my own students’ papers, I remember how Ken would fix every single spelling or grammatical error in mine. I repeat what he told me once at a retreat, after a bit of wine, (paraphrased) ‘you can’t just stand around at a poster session. You simply have to DRAG people over to your poster and CONVINCE them it’s exciting.’ I think about the surprisingly important impact of a late night in the Calvin lab when Ken and Mel came through after a formal event. They were in formal suits, tipsy, jolly, and laughing as they came over to the spectrometer because they just wanted to see what was happening. It was a warm glimpse into relaxed, inquisitive side that contrasted with our somewhat formal group meetings, and along with other vignettes reminds me how helpful it can be for students to see a human side in their PI.

Ken sometimes put new PhD students on unique projects. It is an interesting model, and interesting to reflect on the skills built in the course of trying new directions in a laboratory as a junior graduate student. I loved my initial project in 1984, and it was a total failure. The project was to crystallize the bacterial photosynthetic reaction center from *R. capsulatus*, in collaboration with the Hearst and Kim labs. It was an exciting time, with structures of the *R. viridis* reaction center and subsequent 1988 Nobel prize by Michel, Deisenhofer and Huber for the first membrane protein structure. The Hearst lab had sequenced the *capsulatus* reaction center proteins and hoped to make it a model system under genetic control. Steve Worland taught me a *R. capsulata* reaction center prep. After many mistakes, I became proficient and enjoyed isolating highly pure (by gel) brilliant blue complexes. I set up many hanging drop crystal trials in Sung-Hou Kim’s refrigerated space. Variables included type and amount of detergent, salt, pH, and added amphiphilic small molecules, for which I recall Steve Holbrook devising a trial matrix with, at the time, a novel random number generator. After ~ 2 years my only successful crystal was a beautiful cubic NaCl. Interestingly, although at least five other purple bacteria reaction centers including *R. sphaeroides* have been structurally characterized since then, a structure from *R. capsulatus* only just appeared this year as an RC-LHC1 complex in a cryo-EM study (Bracun et al., Structure 2023). My own final step in this project was an attempt at a ‘control,’ reproducing reaction center membrane crystals from *R. viridis.* I grew *viridis* anaerobically, and when the carboy was opened, the stench cleared the floor if not the building.

Ken was amazingly patient during the initial phase of this work. In retrospect, I greatly appreciate being given the freedom and resources, both then and in future OEC projects after I was rescued by the EXAFS and EPR folks. The lab was generally protected from behind the scenes work of funding and other administrative issues. I'm grateful for Ken’s hard work, steady leadership, and focus on quality and communication that supported a unique, creative group of researchers who have made impacts across numerous fields.

## Chuck Dismukes

### Rutgers University

I worked with Ken as a postdoc from 1976 to 1978, after completing my PhD in radiation chemistry and physics in 1975. I am indebted to Ken for taking a big risk in appointing me. Up to that point, I had not studied any biology, let alone photosynthesis, other than in high school. At that time, I knew that I needed to change my career track after encountering *circa* 3,000 protestors blocking access to the conference venue at the International Radiation Research Congress in Seattle, WA, Jul 1974–future employment was essential! Lucky for me, Ken was trained as a physical chemist and had a history of productive work with physicists like Melvin Klein, who later became my co-advisor in the CB Lab. This seemed an ideal path for me to take—working with two like-minded mentors, leaving the controversial nuclear energy field behind for the more promising field of green energy (later disproven). Ken’s expertise in optical spectroscopy and Mel Klein’s expertise in magnetic resonance spectroscopy matched my fledgling training in these topics during my PhD studies. Plus, there were several other researchers in the CB building from the “Calvin Lab”, including Melvin Calvin himself, from whom I could learn about photosynthesis and photochemistry.

Ken assigned me the task to build a red wavelength-emitting picosecond laser to match the chlorophyll absorption band maximum. Ken and Chuck Harris had proposed ruby as the lasing material and mode-locking to achieve picosecond pulses and a Pockel cell for pulse selection. I set up much of that system from scratch in the basement of Hildebrand Hall adjacent to a Nd-YAG laser system being developed by Chuck Harris and his postdoc, Ahmed Zewail (Nobel prize, 1999). At that time, longitudinal mode-locking was an established technique for glassy solid-state lasers, but not yet demonstrated for crystalline systems (ruby is chromium oxide). I still have unpublished examples of picosecond pulses from this work that I presented at conferences. But the unavoidable heating of the single crystal ruby from the flashlamp excitation took a long time to dissipate before it could be fired again, limiting the flash rate to once every 5 min. That made it useless as a practical tool and quite expensive to replace the ruby crystals that were damaged when my impatience and thermal lensing won the battle. The knowledge gained proved invaluable to me in later years as we built fluorescence and absorbance spectrometers using commercial GaAs lasers.

Fortunately for me, Ken was highly collaborative, and I was encouraged to work on other projects in parallel, notably with Mel Klein, Anne McGuire, Rich Friesner, Harry Frank, and Steve Cooper. Important discussions with Sandy Asher, Clark Lagarias, John Otvos, Vasili Petrouleas, and Ken Raymond’s group further contributed to my education. Those collaborations and friendships led to multiple co-authored papers on chemically induced dynamic spin polarization in spinach photosystem I, the orientation of photogenerated radical intermediates and the excited (bacterio)chlorophyll *triplet state of the primary donor in intact cells and chloroplasts, the determination of the ligand-field strength and spin exchange coupling of the Fe-semiquinone carrier in bacteria, and the EPR and optical spectroscopies and magnetic susceptibility of mixed-valence dimanganese compounds. These friendships have proven to be long-lasting and influential to my subsequent independent career. Ken taught us that helping others is a joy not to be missed.

A few months after I arrived in Berkeley, Ken departed in 1976 for a year-long sabbatical leave in France to work with Paul Mathis (who later became my sabbatical host in 1984). Ken asked me to run the Sauer group meetings in his absence (Yikes, this is before the internet). This felt quite intimidating insofar as I had little experience in photosynthesis at the time. In Ken’s absence, Melvin Calvin often asked me (ordered me?) to review papers sent to him as editor. Surely, this was the fast-track to failure or success, so I thought. To quote Dickens, “it was the best of times, it was the worst of times” for a newly minted PhD from another field. But Ken never let me down, his knowledge of the literature was comprehensive, his door was always open for questions, his enthusiasm and laughter carried us through periods of doubt, his home was often used for group social events, his unshakable belief in the scientific method and scientific integrity were qualities that he gave to us and which continue to define my character. I am especially grateful to him for this last gift. The Sauer group continued to grow after my departure and those who came through it share their own fond memories and inherited the same gifts. Our shared connection with Ken has opened conversations, collaborations and debates among us that might not have taken place had his curiosity and cordiality not been so strongly planted in our hearts and minds. His legacy as inspiring mentor is my fondest memory and arguably his greatest contribution.

## Carmen Fernandez

### University of Sao Paulo, Brazil

Back in 1996, I had the pleasure of receiving acceptance from Prof. Sauer to be a postdoc in his group and work with the water photooxidation reaction in Photosystem II, more specifically with the S_3_ state of the Oxygen Evolving Complex by EXAFS. It was a huge learning experience that I had in your group with the dear Mel Klein, Vittal Yachandra and the group of collaborators and students at the time, Johannes Messinger, Uwe Bergmann, Elodie Anxolabehere-Mallart, Emmanuele Bellacchio, Hendrik Visser, Jana Steiger, John Robblee, Karen McFarlane-Holman, Mary Talbot, Roehl Cinco, Shelly Pizarro, among others. The learning went far beyond the research itself, it was learning about life, about working in groups, about the university, about the country. And, for someone who came from Brazil, this meant a lot and made a huge difference in my training. Prof. Sauer accepted me at the time without even knowing me and I am eternally grateful for the experience of working in a cutting-edge group with an extremely relevant research question. This experience always reappears in my classes with my students. Not to mention the dinners in your cozy home with your lovely wife Margie. I have a lot of affection, and missing those days I spent in Berkeley in Prof. Sauer´s group.

**Figure:**
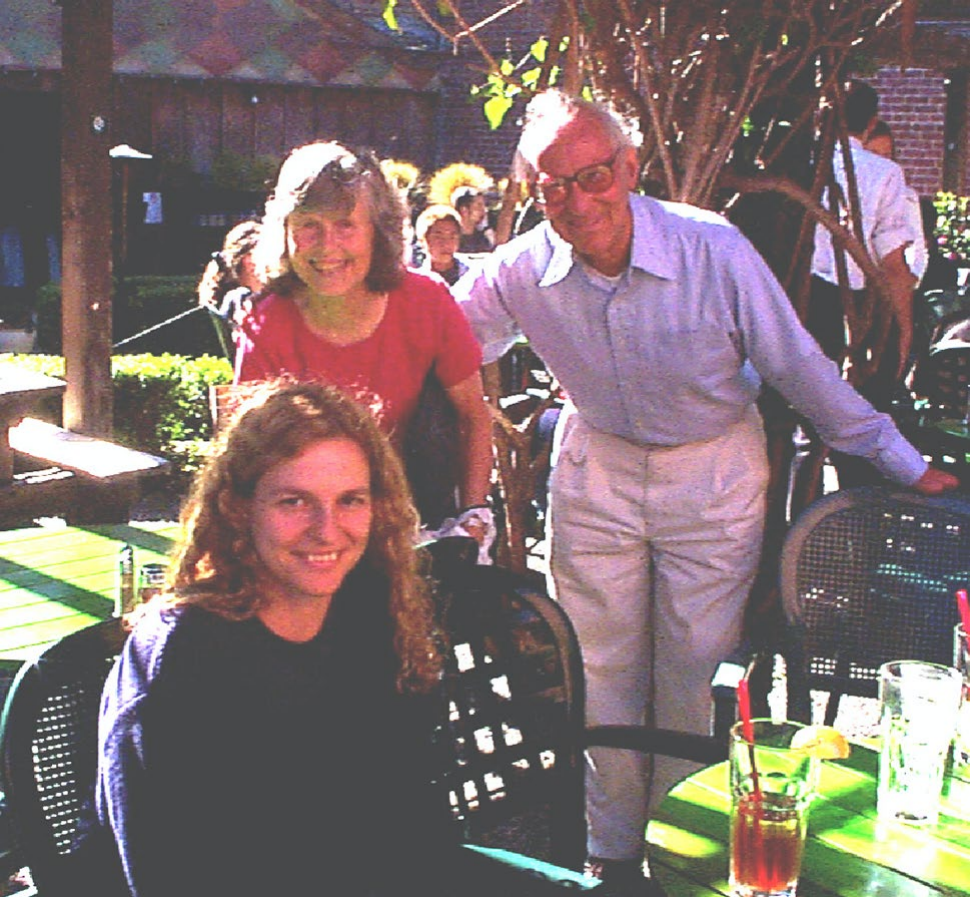
From left, Carmen Fernandez, Margie Sauer, Ken Sauer in the favorite meeting place for the group, Jupiter Beer Garden, ~ 1997. Photo courtesy of Carmen Fernandez

## Graham Fleming

### University *of California*, Berkeley, and Lawrence Berkeley National Laboratory

Ken had a huge legacy of thousands of undergraduates in addition to graduate students and postdocs who are now active researchers in photosynthesis, including some of the leaders in this field.

## Harry Frank

### University of Connecticut

My PhD thesis at Boston University dealt with the mechanism of radiationless transitions in the triplet states of aromatic hydrocarbons and chlorophylls. As my graduate career was concluding, I became interested in learning more about photosynthesis. So, I wrote to Ken asking about a potential postdoctoral opportunity in his lab in Berkeley. As luck would have it, Ken and his group had recently published a paper suggesting that the chlorophyll triplet state was the precursor to charge separation in the photosynthetic reaction center. Ironically, today we know that is not true. In any event, Ken wrote back and offered me a postdoctoral position stating that “It would be good to have someone with your background in triplet states join our group.” I was thrilled to get that letter, and I eagerly looked forward to joining his research group in Berkeley.

The joke I like to tell, which is only half true, is that upon my arrival in Berkeley, Rich Friesner met me at the door of the Chemical Biodynamics Lab and told me that he had “figured out the problem” and that his new interpretation of the data indicated that triplet states were not involved in the primary process of charge separation, so “they don’t need me after all.” Despite that inauspicious welcome, and with the encouragement of Ken and Mel Klein, I set up the equipment to detect photo-excited triplet state EPR spectra. I took spectra from a variety of photosynthetic samples and in the course of these investigations discovered the EPR spectra of carotenoids. Ken’s group had many super-talented students and postdocs, and he set the tone for all of us to have close camaraderie. The group at that time included Mary Blackwell, John Bolt, Chuck Dismukes, Richard Friesner, John McCracken, Ann McGuire, John Nairn, Tom Wydrzynski, and Jacques Breton as a visiting scientist. We all helped each other by exchanging ideas and lending support in our various research projects, although I am sure I gained more from them than they did from me. Also, Ken enjoyed socializing with the group outside the lab, and frequently he would invite us to his house for dinner prepared lovingly by Margie followed by musical entertainment by his sons. Back in the lab, Ken’s scholarly demeanor and contemplative self-critical approach to research provided a superb model for his graduate students and postdoctoral associates. He was an excellent mentor. He taught me to be critical of my experimental data and how to write scientific papers properly. I remember him saying that one should consider every word in each sentence and ask whether that word is necessary. If the sentence reads fine without that word, delete it, otherwise the reader will get tired reading extraneous verbiage. I am unsure whether this present text would stand up to that level of scrutiny. In any case, after three years in Berkeley when it came time for me to leave for my new position as an assistant professor at the University of Connecticut, I gave one final seminar to the lab. Afterwards Ken took me aside and told me he was proud of how well I had matured as a scientist during my time in his lab. That meant a lot to me, and I would not have been as successful as I have been in my career without the strong guidance he provided me during my time in his group.

## Petra Fromme

### *Arizona* State University

Ken was one of the most kind and inspirational scientists I have known. He had so much passion for Photosynthesis and Science and all the people around him. My most memorable conversations with him were from the Western Photosynthesis Conferences in Asilomar, which he regularly attended. We not only discussed the most exciting advances in photosynthesis, but he gave me also deep insights into his science career sharing how much he enjoyed the early time at Berkeley when he and his colleagues could focus completely on the science and new discoveries. His advice was to always put science first and avoid getting into administrative leadership roles, which I took close to my heart. He also was such a kind and caring person. I still remember the conference where I took my daughter, who had just graduated from High School, to the conference and she had a really great conversation with Ken about life, science, career and family. A year later when we met again in Asilomar, he still remembered this conversation and asked me about her, what she is now doing what she decided to study. Ken was such an outstanding scientist and such a kind and caring person that he is greatly missed by all of us!

## David B. Goodin

### University of California, Davis

I was admitted to the UC Berkeley program in organic chemistry, because that is what I had the most experience with at the time. However, soon after entering, it was clear to me that I was more interested in all the wonderful things that were being done in biophysical chemistry. The department allowed me to switch, and both Ken Sauer and Mel Klein agreed to take a chance with my interest in combining X-ray absorption with biochemistry to study the role of manganese in oxygen evolution, all without my having any background in it. I had never taken a course with the word bio in it, and I was certainly not a physicist, so I was excited but also very afraid that people would soon find out that I did not know what I was doing.

There were many qualities that made the combination of Mel and Ken ideal advisors. They were both very patient, fair and steady. They both encouraged me to become independent very early, while also being very engaged in what I was doing. The twinkle in Mel’s eye when he saw that I was doing something that I loved was inspiring, while Ken’s approach reminded me of the importance of having a good and detailed plan to be able to make progress.

Both Ken and Mel encouraged me to develop collaborations that were for the most part, strong, rewarding and lasting. While an early attempt at collaborating with a student at another institution who had claimed to purify the “manganese protein” in PSII became a significant roadblock in my project, Ken and Mel remained patient and supportive in allowing me to get back on track. That hiccup, and seeing their approach to overcoming it became one of the most important lessons I’ve ever learned. Over the years, I have benefited from many valuable collaborations that were formed under the guidance of Ken and Mel, including with R. David Britt, Vittal Yachandra, Jim Fee, Ann McDermott, Vickie DeRose, and many others. Ken’s approach as an advisor, by holding to a high standard of integrity and honesty is one of the most important factors in my development as a scientist.

## Govindjee Govindjee

### University of Illinois

I have had the highest regard for Kenneth (Ken) Sauer, both as a person and as a scientist. He was always forthright and direct and at the same time extremely polite. Most of my meetings and interactions had been at the Gordon Conferences on Photosynthesis, but also at the University of California Berkeley (UCB). His extraordinary research and discoveries on the molecular mechanism of oxygen evolution, involving the Mn complex, is what interested me most. His thoughts were always better than mine- and at each meeting I learnt new things from him. In addition, I was always impressed with him for recognizing others. I remember when he wrote about Thomas J. Wydrzynski (1947–2018), who had obtained his PhD in 1977, in my laboratory, at the University of Illinois at Urbana-Champaign (UIUC), but later did a postdoc stint with Ken. Sauer wrote: ‘Tom proposed, in 1980, that during a specific key transition of the so-called ‘oxygen clock’, bound water becomes partially oxidized, rather than a further increase in Mn oxidation. This ingenious and highly original work was little noted at the time. To my mind, this is an outstanding example of a creative imagination supported by sound chemical thinking at work.’

**Figure:**
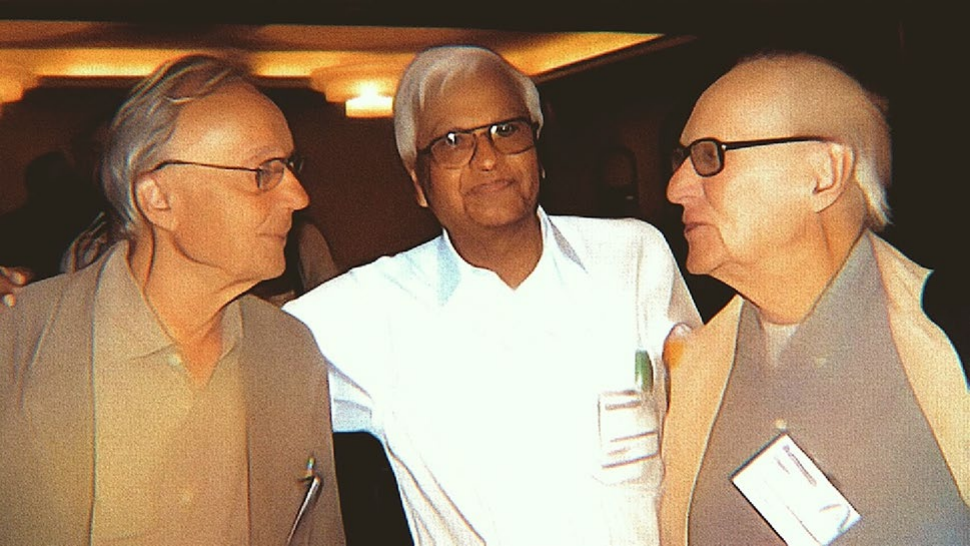
Three winners of the International Society of Photosynthesis Research Lifetime Achievement Award. From left, Pierre Joliot in 2013, Govindjee Govindjee in 2022 and Ken Sauer in 2010. Photo taken at PS16 International Congress of Photosynthesis in St. Louis in 2013. Photo courtesy of Govindjee Govindjee

## Beverley Green

### University of British Columbia

A memorable sabbatical in Ken Sauer’s Lab:’82–83.

I’d met Ken at a couple of Gordon Conferences and read some of his papers. I knew he had recently constructed a single-photon counting apparatus for measuring fluorescence decay that was able to resolve three individual decay components but had not been able to assign them to specific photosystems. I’d been working with two of Don Miles hcf (high Chl fluorescence) mutants of corn which were completely lacking either PSI or PSII. I suggested to Ken that they could be helpful in assigning the three decay components, and asked if I could come to his lab on sabbatical in Sept. 1982. I think he was a bit dubious at first about having a hard-core biochemist gel-runner in his lab, but he agreed.

Ken’s group had students and postdocs from a variety of backgrounds, mostly physical chemistry but from very theoretical to practical experimenters. I worked with Kerry Karukstis, a very enthusiastic and energetic postdoc. Getting material for the proposed experiments was not a simple matter of going to the nearest grocery store. A homozygous mutant of either PSI or PSII is seedling lethal. Here’s where corn is a great subject because of its large seed reserves which allow a mutant seedling to survive to the 3 or 4 leaf stage. The only way to get mutant seedlings is by planting a lot of seeds from a heterozygous parent plant (cob), which will segregate about a quarter seedlings that are very pale and highly fluorescent when examined under UV light in the dark. As a bonus, the other three-quarters of the seedlings are a built-in positive control, so all the fluorescence runs were done with mutant and normal plastids prepared from the same batch of plants on the same day. So what did we find? Well, to our surprise, both mutants showed all three decay components, and it became clear that each component had multiple origins!

Ken was an excellent model as a supervisor. He kept track of what each person in the lab was doing, in addition to the weekly group meetings. When he found an interesting paper, he would print it out and put the names of people he thought should read it at the top, and you had to check yours off! He was (almost) always calm and non-judgmental. Which reminds me of the day I was sitting next to Ken at a Gordon Conference and we had just suffered through an absolutely awful talk where the guy (it was mostly guys in those days) didn’t have a shred of decent evidence for what he was proposing. I was shocked to see Ken carefully writing a sentence in his notebook about what the guy thought he’d proven and said “Do you really believe that?” and with his gentle smile he said “No!”.

## Martin Gruebele

### University of Illinois Urbana-Champaign

Ken Sauer's lab in the Melvin Calvin building was my first exposure to biophysics research. Although I strayed from it into gas phase spectroscopy for years, I eventually returned to biophysics, not least because of the great experience in Ken's group. I arrived on the Berkeley campus fresh out of high school in San Francisco, and jumped into Chem 4C in spring of 1982. Ken was teaching the class, and a fine teacher he was. He gave interesting lectures with anecdotes that could not be found in a book, and kept the level fairly high and the speed breezy but not overwhelming. I thought he and George Pimentel were the best teachers in Chemistry. I enjoyed the material for the quarter (no semesters yet back then), and immediately asked him if I could do summer undergrad research with him, for I had heard 'that is the thing one should do.' He interviewed me personally and assigned me to a synthesis project: using the Grignard reaction to make octane-1,2,3-triol that grad students and postdocs would use as an additive detergent for crystallizing a prokaryotic photosystem (Vickie's *capsulatus,* I think). I was at the hood twice a week, and got fairly proficient at doing air-sensitive reactions, making a reasonably pure recrystallized product. Ultimately we were thwarted by the German team in getting a structure, but the excitement was palpable. Ken and his entourage had of course many more experiments going on, including new-fangled time-resolved spectroscopy and I got to see a lot of high-tech toys. It really changed my view of biology and chemistry, which had been very 'ecological' and 'synthetic'. I liked the physics approach. Ken talked to me pretty regularly, which I did not realize at the time was actually very unusual for a professor and a summer undergrad. I saw someone who just had fun doing science and cared not about getting the answer he thought was right, but whatever the answer actually was. I applied this later in my own research group, explaining to students that there are only 'good results' because they are the real answer if experiments are carefully done. So I really felt that he cared and that I was part of the group. In the fall, it was back to classes, but I kept up regular lines of communication with Ken, indeed even when I visited campus decades later as a faculty member. I was very sad when I came by again in 2022 for a seminar, but Ken was ill and we could not meet.

## Robert Knox

### University of Rochester

I spoke with Ken Sauer at many meetings and was always impressed with his comprehensive view of the physico-chemical subject at hand, as well as with his smooth and kindly demeanor. I considered him a “scientist’s scientist.”

## K.V. Lakshmi

### Rensselaer Polytechnic University

I was really sorry to hear that we lost Ken Sauer. He made so many seminal contributions to the field and trained a long line of stellar scientists!

## Wen Liang

### Stanford University

I was a graduate student in Ken's lab in the early '90 s. At one of Ken's famous Christmas gatherings at his house, several of us were playing "Blockhead!"—a simple tabletop game where the goal is to add blocks to a tower without causing it to collapse on your turn. Although Ken wasn't participating, he sat with us on the floor and watched us for a long time. Later, he told us that he enjoyed observing the different personalities of his students through this game. I eventually realized that he was interested in learning more about his students and lab members, which might have helped him guide us according to our various personalities. I've since appreciated his efforts, especially as I've worked with more PIs and learned that not all are willing or able to do so.

No matter how busy he was, Ken always made time to listen to us with kindness and patience. Whenever I had a question and needed his advice, I knew I would receive his full attention when I entered his office. I consider myself lucky to have had Ken as my thesis advisor.

## John McCracken

### Michigan State University

As an undergraduate at the University of Illinois, I had developed interests in molecular spectroscopy and biophysical chemistry, and was hoping to find a graduate research program that would build my knowledge in both areas. I chose UC Berkeley for graduate studies because there were several faculty members whose research programs would allow me to realize that goal. After the Chemistry Department’s six-week “research director rush,” I was incredibly lucky to land in Ken Sauer’s Lab. Ken was a skilled research mentor. In our first meeting after I joined his group, he told me about the various projects ongoing in the lab and asked me what sounded most interesting. I selected the photo-excited triplet state EPR studies that were being performed by Harry Frank and Mary Blackwell. Ken took me out into the lab, introduced me to Harry and told me to work with him until I got to know my way around the lab. I didn’t realize it then, but Ken wasn’t going to frame a research project for me, he was going to let me develop it, or drift into it, on my own!

That same week, I attended my first Sauer research group meeting as a full-fledged member. At the start of the meeting, Ken passed around a sign-up sheet for the next quarter’s meetings. Because I was new to the lab, I didn’t sign-up to give a presentation. Well, as the meeting concluded, Ken looked my way and said, “John, you didn’t sign up for a meeting! Don’t worry, I’ll give you a few articles and you can base a presentation on them.” Of course, the only time slot left was the first week of the quarter! One of the articles that Ken gave me was a short paper from the Argonne Photosynthesis group on pulsed EPR studies of a bacterial photosynthetic reaction center. I not only chose to do my group meeting on that paper, but I spent the rest of my scientific career working on pulsed EPR.

Ken Sauer was an excellent teacher in both the lab and the classroom. While Ken’s background was in physical organic chemistry, he maintained a strong working relationship with Mel Klein, a Professor in the Physics Department and an LBL Principal Investigator. Often, the Sauer and Klein research groups held joint meetings so their students benefited from the broad range of scientific expertise available when presenting their work. There’s a second story that comes from my first Sauer group meeting. During my presentation, I mentioned that the time response of the EPR measurement was determined by the Q of the EPR probe. One of the older students from the Sauer group asked me if I understood what that meant. I had to admit that I didn’t know. The student went on to say that they didn’t know either. Mel Klein smiled and asked me a simpler question, “Do you have a general physics book at home?” I answered positively and he told me to go home and read about the RLC circuit. I followed his instruction and discovered the answer to the question. Both Ken and Mel preferred to answer questions by guiding you towards the answer. It was a great way to learn and it would be repeated many times over the next five years. Within a month of joining Ken Sauer’s lab I knew I’d found the best place to earn my doctoral degree.

## Ann McDermott

### Columbia University

I had the great privilege of working under Kens Sauer’s leadership from 1982 to 1988. While hopefully others will provide an accounting of his impressive vitae, training and publications, I will offer a glimpse of his mentorship style. Or his style as I experienced it, being relatively green about large excellent research environments, and being a female student at a time when we were still a slim minority. While some of the reasons female students did not want to stay in the program were on open display, and eccentric or counterproductive mentoring was common, Ken gave sensible avuncular advice. When interviewing, the question came up about his willingness or interest in taking a female student (as some of his colleagues would not) and he told me something memorable: he took gifted and committed students, and as far as the climate of his group (which I had not even asked about) I should ask his senior female student, who he probably knew would have lots of colorful things to say, but none of them negative about Ken! When later on I seemed to be stubbornly doing all-nighters focused on a technical impasse, he then told me I had taken the “committed “ bit too literally, and prescribed a dose of R&R and another of deeper reflection on my research goals. And towards the end, I hit a more productive rhythm and could be of more help to others in the lab, he offered the only threat of those great 6 years– he would lower my pay if I did not come up with some better ideas about my next stage of training. This direct instruction from him was very rare, I think these might have been the only instances, and yet very well chosen for me at the time –- I think of those few pieces of advice essentially daily in my work.

In the main he led by example, and my read of his values and silent instruction begins with “choose a first class problem”. The questions he pursued about the design principles of solar energy conversion systems have and will stand the test of time: a scientific puzzle so daunting, seductive, and so relevant that it asks the very best of our collective effort. In this pursuit, I remember him as being and asking us to “be relentlessly curious about the truth”. When your time came to lead a group meeting, God help you if you had anything less! Evidently, in his mind, “Truth is grounded in the laws of chemistry and physics”. You could guess that from his high standards in group meetings and manuscripts, but just in case there was any doubt, we can remember the time a foreign visitor was lecturing and Ken punctuated a somewhat fanciful two hour presentation by explicitly reminding him so! But that memory also underscores how his laboratory attracted fine minds, with fresh perspectives, different training, various eccentricities, and especially with all sorts of latest spectroscopic or biophysical methods from all around the world. Intrepid, he had incisive conversations with all comers. To be in his group was to be at the table with a very large, very smart family, starting with his own gracious and accomplished family, to all of his long-time devoted collaborators, down to the most recently arrived undergraduate. And at that table we saw daily that it is possible to compete and cooperate for this great game of Truth finding, and at the same time retain our decency and humility. That family is a great gift that brought me into biophysics, mentored my initial bumbling efforts, and continues to inspire me today.

## Tasios Melis and Kris Niyogi

### University *of California*, Berkeley

We enjoyed having Ken, as a Professor Emeritus, present and active in many of our Plant and Microbial Photosynthesis seminar class meetings, which took place on Wednesdays in the Fall semester, from 2–3 pm at Berkeley. Students and professors alike benefited from his insightful comments and reference to literature. We missed him, when health reasons prevented him from attending during the most recent years.

**Kris Niyogi** (adds his own personal recollections in addition to the one above with Tasios Melis).

### University of California, Berkeley, and Lawrence Berkeley National Laboratory

In addition to many wonderful scientific and classroom interactions that I had with Ken, the two of us served together for over a decade as co-chairs of the Evaluation Committee and members of the Executive Committee of the France-Berkeley Fund. This seed grant program provides funding for nascent collaborations between researchers in France and Berkeley across a wide variety of disciplines, from science and engineering to humanities and social sciences. It was through this service that I learned about Ken's passion for international scholarly exchange, and I got to hear many fascinating stories about the time that he and family spent early in his career at the American University of Beirut in Lebanon. The world is a better place thanks to Ken's inspirational commitment to fostering international scientific discussions and collaborations!

## Paul Mathis

### CEN Saclay, France

I first met Ken Sauer at the 1st International Congress on Photosynthesis, at Freudenstadt, Germany, in June 1968. That seems like a long time ago. Although I was a real beginner, hardly able to speak a few words of English, I felt free to ask him several naive questions after his important lecture on the CD spectra of bacterial reaction centers, where he showed that the primary electron donor is a dimer of bacteriochlorophyll. Ken was kind enough to spend a fair amount of time explaining his results. The second occasion was when I arrived at San Francisco with my family (my wife and three young boys, aged 5, 3 and 2), with hardly any experience of traveling; he had his big Peugeot car and he drove us to Berkeley where Margie had reserved an apartment for us. These comments are just to recall how Ken was helpful and easily accessible, although he was a recognized scientific authority.

Ken, with Margie and their four boys, spent a year with us in Saclay in 1976–77. We visited together many places in our beautiful country (I remember the Fontainebleau castle and the Dordogne region, where my family moved with a car while the Sauer family rode their bicycles). That year we also had a fruitful time in the lab, discovering that the acceptor side of Photosystem I is much more complex than it was supposed at that time.

## Sabeeha Merchant

### University of California, Berkeley

I saw Ken at the western photosynthesis conferences each year and the GRCs. He was kind and welcoming with the most angelic smile. I know my work was far afield from his own expertise and interests, but he always showed interest and enthusiasm. He is remembered and the memory is cherished.

## Ishita Mukerji

### Wesleyan University

I came to Berkeley (1985–1990) from a small liberal arts institution and after a short stint of working at Bell labs. I was young, naïve and interested in Physical Chemistry, I encountered many professors who intimidated me, were condescending and made me feel like I didn’t belong. In my first visit to Berkeley as a prospective graduate student, Ken was gracious, warm and friendly, the antithesis of many of the other faculty members. Over lunch at the faculty club, he told me about the flash experiments, the Kok cycle and the different S-states. I remember being enthralled and eager to join the group. When I saw that he had other women students, I knew it was the right place for me. Ken was instrumental in my decision to attend Berkeley and a wonderful mentor and role model to me as I went forward in my career. I always appreciated that he focused on the science and judged us by our scientific abilities.

Ken’s mentoring often included exacting questions in group meeting (you really needed to know your science!), dinner parties at his house, and camping trips. Of course, there were many challenges along the way–it wouldn’t be graduate school without them, but Ken’s support never wavered. He gave me a lot of freedom to pursue my scientific interests, which in retrospect was unusual but beneficial in terms of turning me into an independent scientist. It was a wonderful experience to be in the lab, the convivial and collaborative atmosphere led to some lifelong friendships that I cherish to this day. Over the years as I have mentored my own students, I’ve tried to remember the mentoring lessons from Ken. The emphasis on excellence while also recognizing the importance of educating and training the whole person continue to be the qualities that I try to emulate. I will always be grateful to Ken for the research opportunities, the training and mentoring that he gave me.

## Annette Rompel

### Universität Wien, Austria

I worked in the lab of Mel Klein, Ken Sauer and Vittal Yachandra from 1993 to 1996 supported by a grant from the German Science Foundation (DFG Forschungsstipendium). This was my first postdoc and first visit in the US. Ken picked me up from SF airport on labor day 1993 and I had the luxury to stay in his house until I rented my apartment (Berkshire Arms) on Delaware street. My research stay in Berkeley had been very valuable for my future career: I got introduced to the important subject of photosynthesis and it deepened my knowledge about x-ray absorption spectroscopy. Ken taught many students (me included) how to present research data in seminars. He contributed with his deep physical chemical knowledge to paper writing and correct citation of references. I try to follow up on this until today. On a personal level I thought/think/will think every year about Ken and the Berkeley group as his birthday is one day after mine.

## Yulia Pushkar

### Purdue University

I had the pleasure of interacting with Prof. Ken Sauer in 2004–2008 while a postdoc at Berkeley. At that time Ken worked on figuring out the Mn–Mn fragments from different Mn minerals which might resemble the geometric structure of the Mn_4_Ca cluster in Photosystem II. He was such a nice person from the old school. He had a beautiful house and was very hospitable. My husband learned Ken's favorite beer (Pale Ale) when we were at his house. Since then, we always have it in the fridge. Also, Ken always had a very healthy lunch box with carrots and dark bread.

## Bill Rutherford

### Imperial College, UK

For me Ken was a kind of Godfather of the Biophysical side of photosynthesis research.

Of course, Mel Klein was an important part of that (and was a special source of encouragement and inspiration to me), but the number of students and postdocs that came through his lab, and then went on to make their individual contributions to the field, always seemed unmatched. And that, in itself, was an outstanding contribution, even before we start talking about the many discoveries that came from the Sauer lab.

## Kirk Schanze

### University of Texas at San Antonio

I worked with Ken and his group in the Calvin Laboratory from 1984—1986 as a Miller Fellow. It was a great experience, providing me with the opportunity to work independently on projects of my own design. Ken was always supportive, even though my work in his lab was focused on topics distinct from his main interests in photosynthesis. I also truly enjoyed the interdisciplinary nature of science that was going on in the Calvin laboratory, with groups in the same space working on science in areas ranging from synthetic and physical chemistry to biochemistry, biophysics and biotechnology. In retrospect, I have always felt that the time in Ken's lab provided me with tools to hit the ground running when I began my independent faculty career in 1986.

## Hugo Scheer

### Ludwig-Maximilians-University of Munich

The first meeting with Ken Sauer was somewhat stressed. He had submitted to JACS a paper on the structure of bacteriochlorophyll b that was rejected for insufficient proof. We had proved, by nmr, that the structure he had proposed was correct and published this, again in JACS. Ken had then invited me to Berkeley, at the end of my postdoctoral time at Argonne, for a seminar, unspokenly meaning me to talk about the structure. Considering this insufficient for a presentation, I talked instead on chlorophyll aggregation, not realizing how touchy a subject this was between Berkeley and Joe Katz’s lab at Argonne, where I had spent my postdoc. The talk was met with reservation. Ken tried to mediate, but we parted indifferently.

In the following years, we met occasionally at conferences. Friendship developed when he spent almost a year in our lab in Munich as a Humboldt fellow. He also interested his son Peter in our project on calculating energy transfer, who used our just installed computer network as a parallel system with him as integral part. The work carried out then was the starting point for a conference series on photosynthesis research that runs until today. Our families went hiking and biking together with the Sauers, Ken also joined the lab crew in hiking the foothills of the Alps. He only had a problem learning German: whenever he approached somebody with a fairly well pronounced and grammatically correct question, he got the answer in English. Margie proved more lucky because outside the lab there were at least some people who did not respond automatically in English.

Afterwards, we got together at many meetings; scientists are lucky in that respect. He also got me as a Miller fellow to Berkeley where in our free time we took up hiking and biking again. I just mention a trip to the Russian River valley where we just used our sleeping bags on bare ground, overwhelmed by a sky where the common constellations were difficult to spot because the outnumbering multitude of minor stars visible to the naked eye, and waking up with the bags covered by the morning dew.

Finally, let me mention a gorgeous visit to Madsen castle in Austria that Ken provided generously to us and a second couple of German friends, the Haehnels. We spent several days together, exploring the environs, but also the library and the building which was only partly renovated at the time. At one point, we discovered early in the morning an opening behind the tiled stove which led us to a range of barely touched rooms that, still in our nightgowns, we explored one after the other.

The memory works in pictures that are often erratic. Feelings are more integrating. The feeling remaining of Ken is that of an open mind and a sharp intellect combined with personal warmth.

## Sarah Tabbutt

### University of California, San Francisco

I have known Ken since my childhood. He was a family friend as he attended college with my mother and graduate school with my father. During one trip to Berkeley to visit the Sauers in 1972, the kids (8 of us, oldest 13) became so annoying that the parents put all the kids on the newly built BART (Bay Area Rapid Transit) which we rode all day.

I worked at Princeton with Chuck Dismukes the summer after college–Chuck had trained with Ken. Thus, it seemed natural for a shy kid from Washington State that Ken Sauer was the safest bet for a research director when I reached Cal for graduate school. He guided me through photosynthesis, complex mathematics, quantum physics. Always encouraging, but I recall disapproval when he would hear that I went for too long a run while my gel was running and the chloroplasts had run off the bottom (a common occurrence). My research focused on electron spin resonance. Much to the dismay of Ken and my family, I finished my PhD (1987) and headed for medical school to learn about magnetic resonance imaging (which was transitioning to the clinical arena at that time). My first rotation in a dark radiology suite and that was the end of MRI.

After years of training and working out East, I eventually came back to the Bay Area and reconnected with Ken and Margie. Ken appreciated how much I loved my career, though he did seem a bit disturbed that I no longer understood my PhD thesis. Ken instilled an appreciation of balance between work and other interests. He was brilliant, positive, generous and warm.

A recent fun Sauer fact. The Alaska Air flight out of Portland that lost its door mid-air in January 2024; well it fell into Ken Sauer son’s backyard.

## Steve Worland

### Agouron Pharmaceuticals

I worked in Ken's lab in the first half of the 1980s. Like all first years, I rotated through a few labs and set my sights on joining Ken's lab based on the super interesting problem of photon-induced charge separation and Ken's apparent willingness to take on a wide array of problems with multiple scientific approaches. One of the first days I was ready to run "my own" experiment, which required preparing chromatophores in the French press, Ken came by my bench and encouraged me to pause my work and walk over to join the 10:30 tea held on the second floor of the Round House. I explained that I was just ready to activate the press, which John McCracken liked to remind me was really an explosive device, so I demurred and Ken was OK. A couple of days later he came by again and encouraged me to join the tea and I remember thinking: this guy wants me to stop an experiment to go get a cookie (?) but hey, I joined his lab for a reason so I'll give it a try. I immediately got into a conversation with Mel Klein as to whether there was such a thing as centrifugal force or was it just a construct made up to explain a body's behavior in the absence of other forces (my position). Mel deftly directed the conversation towards spin and magnetic resonance spectroscopy. The next day I met Jim Bartholomew, proud owner of the first cell sorter on the Berkeley campus. George Pimentel, John Hearst, Sung-Ho Kim, brilliant students, postdocs and PIs from these and other labs—it was a remarkable variety of keen minds who gathered every day to discuss any and all aspects of science. And Ken was often as interested in reviewing the discussion that had occurred at morning tea as he was in discussing my data.

Years later after I'd been in many different settings far removed from photosynthesis, I gained enough perspective to realize that Ken's push to join the tea times was one of his many ways of teaching and of setting expectations. He fully expected me to desire what I would characterize as a liberal arts education in science. I was expected to find most science questions inherently interesting and to seek out chances to engage the scientists who pursued these questions as a means of learning and stimulating my own thinking. The breadth of different disciplines was in itself the point, and the challenge as well as the opportunity to learn and expand. Life was more interesting at an interface than square within one discipline. As multi-disciplinary science has gained in popularity I realize how far ahead of the times Ken and the other leaders of the Round House were in setting up such a diverse building, and even today I continue to appreciate how much the push to join tea time whetted my appetite to always broaden my horizons.

## Kizashi Yamaguchi

### Osaka University

Prof. Ken Sauer has been one of pioneers of the physicochemical research based on the quantum mechanics (QM) in photosynthesis. I can remember the discussions with him on the water insertion step at Berkeley.

Our basic science approach to Mn oxides was started near 1980 when Prof. Ken Sauer wrote a review article on Mn and oxygen evolution (*Acc. Chem. Res.* 1980). At that time, MnO_2_Mn and porphyrin (Por)Mn = O were experimentally investigated for catalysts for oxygenation reactions and water oxidation. Therefore, our theoretical investigations on the M = O and M–O-M bonds were also initiated at that time. Indeed, our theoretical studies on Mn oxides are closely related to those of the experimental methods of Berkeley groups as shown in this review.

Photosynthesis is one of main research themes in our Osaka University. Prof. Tsubomura opened a research center (the next building of my office throughout 50 years) of the artificial photosynthesis (1980) in our Osaka University. Prof. M Calvin visited to the center in 1981. Therefore, in the center, we can see the monumental card written as “A Step toward the Peace of the World “ presented by Prof. Calvin at his visit to the center; Dec. 2, 1981. On the other hand, Inoue opened at RIKEN at Wako a research center for the oxygen evolving system of photosynthesis involving Mn complexes.

## Athina Zouni

### Humboldt University Berlin, Germany

I remember Ken Sauer very well because we often discussed the PSII structures with respect to the water splitting mechanism in the group of Vittal Yachandra and Junko Yano (Berkeley Laboratory). These were really fruitful discussions, as he shared his long-standing expertise in the field of photosystem II with us. Especially in the structural analysis of PSII, he always provided us with a lot of important information. We were part of his family, so he always invited us to his house and his huge beautiful garden, where we all felt very happy and the fresh strawberries always tasted wonderful! I remember him not only as an excellent scientist, but also a brilliant personality, and a great person!

## Robert Blankenship

### Washington University in St. Louis

I came to Berkeley in the fall of 1970, a small-town kid from Nebraska. Needless to say, it was quite an abrupt transition in many respects. Our main task as Chemistry graduate students in the first quarter was to find a research group to join. After much uncertainty, I finally settled on joining Ken’s group just before the deadline. He was reluctant to take me, as he had already taken several other students that year. Fortunately, he relented and accepted me to be a student.

My project was to use photoelectron spectroscopy, sometimes called ESCA (Electron Spectroscopy for Chemical Analysis) to investigate the Mn in Photosystem II. I spent much of my first year analyzing model Mn compounds at the Field Free Lab, halfway up the hill to LBL. Finally, I was ready to analyze chloroplasts. This was in the days before BBY particles, so the sample was not enriched in any way. The results were disappointing, as no signal could be seen at all. The problem was that ESCA monitors the photoelectrons emitted after X-ray irradiation, and essentially only surfaces can be probed, as the emitted photoelectrons are strongly absorbed and scattered. Of course, later the Sauer/Klein group had great success in monitoring the Mn using X-ray absorption techniques. This is basically the same experiment but monitors the absorbed X-ray instead of the photoelectron produced by the absorption. This is a bulk technique and doesn’t suffer from the surface problems.

At this point, I needed a new project. I had become very interested in the Mn problem and wanted to continue studying it but wasn’t sure how to proceed. Fortunately, at just the right moment a paper appeared from Warren Butler’s group (Lozier et al. 1971), showing that various agents released the Mn from its site, and it was then able to be monitored using EPR spectroscopy at room temperature. This started me on a new track and served as the basis for my dissertation work. Jerry Babcock was a student in the Sauer group and had served as my informal mentor when I joined the group. He was in the process of doing research on EPR Signal II and showed me how to use the old (even at that time!) Varian E-3 spectrometer in the lab. Ken was very supportive during this difficult time for me and allowed me the independence to find my path forward on my own.

One of the most memorable aspects of being part of the Sauer group were the many excursions and socialization events that Ken and Margie provided for the group. My wife Liz and I consciously emulated that approach later after I had a research group of my own, although I don’t think we ever quite came up to the standard that they set!

## Johannes Messinger

### Uppsala University, Sweden

I worked in the lab of Vittal Yachandra, Mel Klein and Ken Sauer from 1997 to 1999 supported by a grant from the German Science Foundation (DFG). This was my third postdoc, after having worked with Tom Wydrzynski’s in Canberra, Australia as well as the Mike Evans and Jonathan Nugent’s in London, UK. Since Tom, years back, has also been a postdoc in the Sauer/Klein lab, this makes me both a scientific son and grandson of Mel and Ken. The time in Berkeley has been transforming for my career: I not only learned about x-ray spectroscopy, but it opened the doors to a wide scientific network and resulted in a friendship and continuous trustful collaboration with the Berkeley team, now led by Vittal Yachandra, Junko Yano and Jan Kern. While I was scientifically most closely associated with Mel and Vittal, Ken and Margie have been tremendously important for the great experience my family and I had in Berkeley. While Margie organized many events for the accompanying family, Ken contributed to my work with sharp questions like: ‘have you thought about the polarization direction in the design of the laser flash setup?’ (which I did not), and his cheerful and considered presence set a great tone for all. The visits in Ken’s and Margie’s wonderful house and garden with the entire group and family remain wonderful memories.

## Junko Yano, Vittal Yachandra, Jan Kern

### Lawrence Berkeley National Laboratory

We three, Vittal, Junko and Jan, started at very different times, Vittal in 1982, Junko in 2001, Jan in 2008, working with Ken, and it has been a distinct privilege to work with him. During this time, Ken taught us many things, and probably one of the most important things that we all learnt from him was not to pay attention to artificial divisions of rank or to pay attention to accolades. The only thing that mattered to him was to keep one’s head down and do good science and present it in a simple manner without flourishes, always sticking to what the data showed. Importantly, for Ken the standing of a person did not matter at all: we have seen him treat senior people or any undergraduate student in the group, with the same intense focus on the science without regard for their position. Similarly, he was convinced that good science will prevail no matter the name of the journal one publishes in or how famous the group that was doing the work, and he was not shy to clearly state problems or shortcomings in any scientific work, may it come from inside the group or from anyone else. The Sauer, Klein, and Calvin ethos of interdisciplinary research was instilled in all of us, enabling us to continue this tradition within the Molecular Biophysics and Integrated Bioimaging Division (MBIB) Division at LBNL (the present incarnation of the Chemical Biodynamics Division from the Calvin Lab and interestingly Junko Yano is the Director of this Division).

It was so impressive to see Ken’s total lack of ego, when he dealt with any of us or with the group. We could criticize his comments or his interpretations and he never held it against us. He was a humble person and always unfailingly decent, who never showed off or tried to impress people with how smart he was. It was an intrinsic part of his character that is something that is very hard to emulate, although we all try.

We particularly enjoyed how he made it relaxing and fun to talk about science, and we did it at every opportunity we could get. We cherished having lunch with him almost every day when our schedules permitted, or when we had alumni or collaborators visits our group as seen in the pictures here. He really enjoyed this time with us, and we got to see a Ken who was usually relaxed and we would talk about everything under the sun (and not only photosynthesis!).

**Figure:**
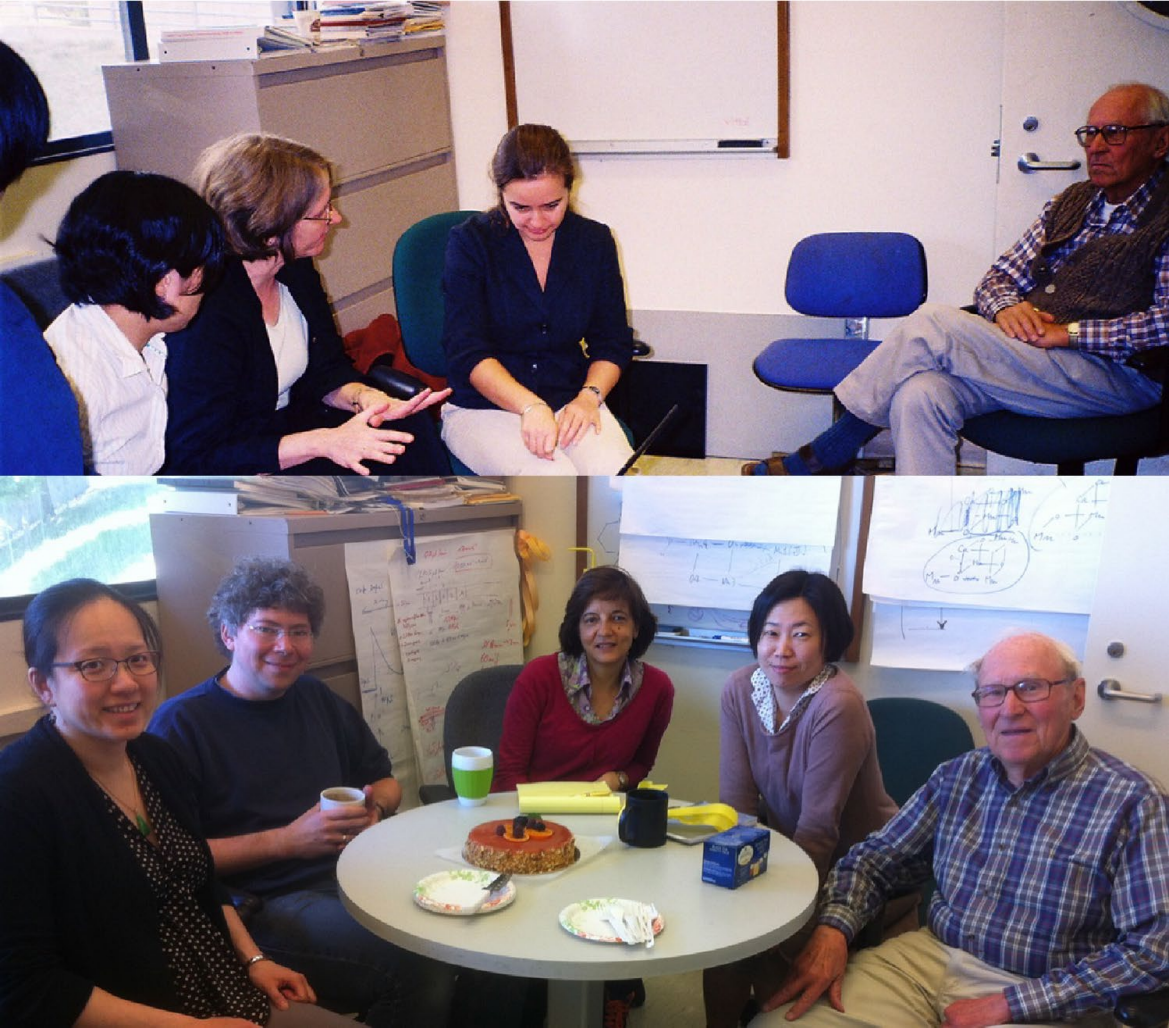
*Top*: From Left: Junko Yano, Ann McDermott, Yulia Pushkar, Ken Sauer in 2007. *Bottom*: Ken Sauer (right) with our collaborator Athina Zouni (center) in 2014 on one of her many visits to the lab. The others are (from left): Rosalie Tran, Jan Kern, and Junko Yano. Photo courtesy of Vittal Yachandra

Ken had many interests and activities outside the lab and avidly shared them with us. Whenever any of us were on travel, he would immediately produce his old maps and brochures describing how lovely the place was and how to visit all the highlights. He and his wife, Margie, sang in local choirs until just a few years ago and he particularly enjoyed Baroque music and often invited the group to come to these concerts. He loved food, which was a great excuse to get together with friends and connect. He was always keen on joining not only our daily lunches but also any group dinners for seminar speakers or when there were visitors to the lab. Gardening was one of Ken’s pleasures and passion. Especially in the last years, when he was more at home and less mobile, he still appreciated working in the garden as much as possible.

**Figure:**
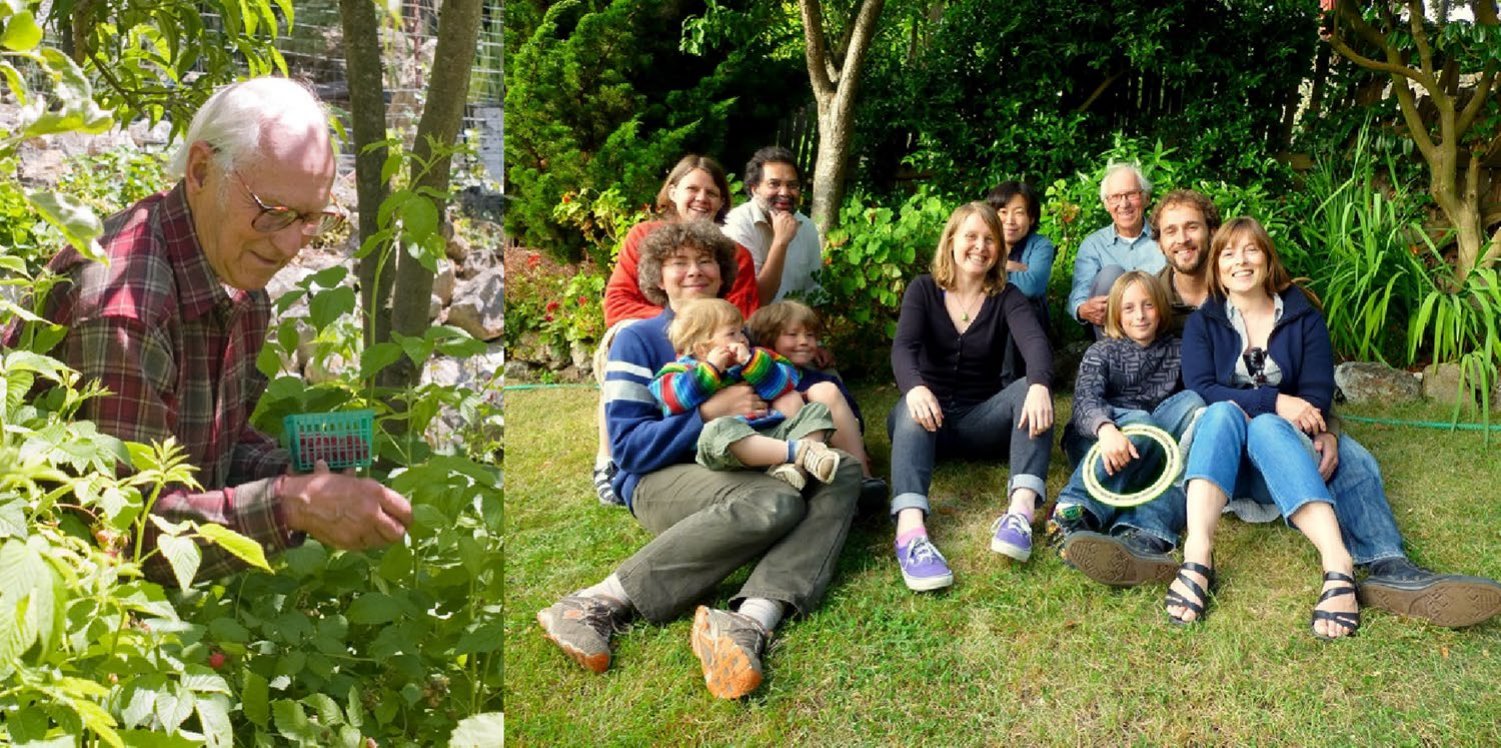
*Left*: Ken picking raspberries in his garden along with the group, 2010. *Right*: In Ken’s garden after picking berries 2010. From left, Jan Kern and family (Silke, and kids Nils and David), Vittal Yachandra, Megan Shelby, Junko Yano, Ken, Ben Lassalle and family (Etienne and Cecille Lassalle). Photos courtesy of Vittal Yachandra

Ken’s backyard was a place to gather for the lab, which was like a huge family with all the children of the group members taking over the garden. His time spent traveling meant that he was comfortable with many cultures and could easily bring different people together. At these occasions, often fresh blueberries and raspberries were picked and enjoyed with ice cream. Many major events in the life of the lab and its members—weddings, baby showers, births, and professional accomplishments were celebrated there, with Ken and Margie as the gracious hosts. Ken and Margie also welcomed each of us into the 'family' when we arrived at Berkeley and helped us and any new member of the group in many ways getting accustomed and settled-in in a new environment. Being part of this lab-and-beyond family made the transition a lot easier, especially for those of us coming from further away, and contributed importantly to us feeling that Berkeley and the Berkeley Lab could become our new home.

**Figure:**
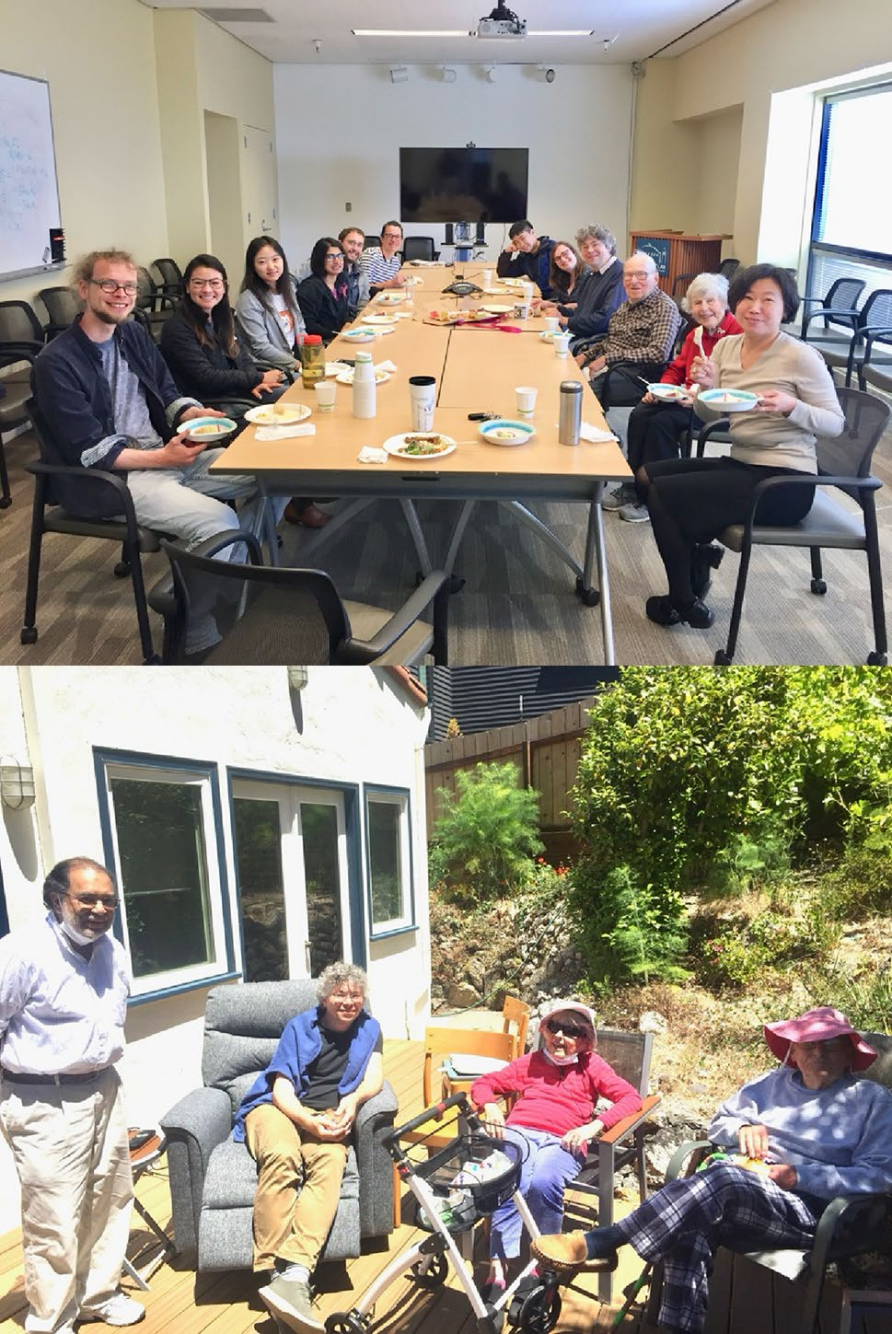
*Top*: Ken and Margie on one of his last visits to the lab and to his office in building 66 on the hill site. This was in March 2019, a few months before the start of the pandemic. The picture was taken during a group meeting in 2019 where Ken and Margie enjoyed eating cake together with students, postdocs and colleagues. From Left: Philipp Simon, Cindy Pham, Wen Hui, Ruchira Chatterjee, Steve Keable, Louise Lassalle, In-Sik Kim, Isabel Bogacz, Jan Kern, Ken, Margie Sauer, Junko Yano. Bottom: *Bottom*: Ken and Margie in May 2022 in their beloved garden, the first time we were able to get together after the pandemic – and the last time many of us were able to get together with Ken. Jan Kern and his family were able to be of help with grocery shopping etc. for Ken and Margie during the pandemic, when life was quite difficult for them. From left: Vittal Yachandra, Jan Kern, Margie and Ken Sauer. During this get together, Ken was interested in hearing about the new XFEL work and was very happy to hear that Junko Yano was away at a symposium on Bioinorganic chemistry, and we were happy to be able to tell him how important his wonderful review on the role of manganese in oxygen evolution from 1980 was, which got so many of us interested in the topic, and thanking him for being such a fantastic role model and an inspiration. Few months after this picture was taken, Ken passed away peacefully at the age of 91. Photos courtesy of Vittal Yachandra

**Figure:**
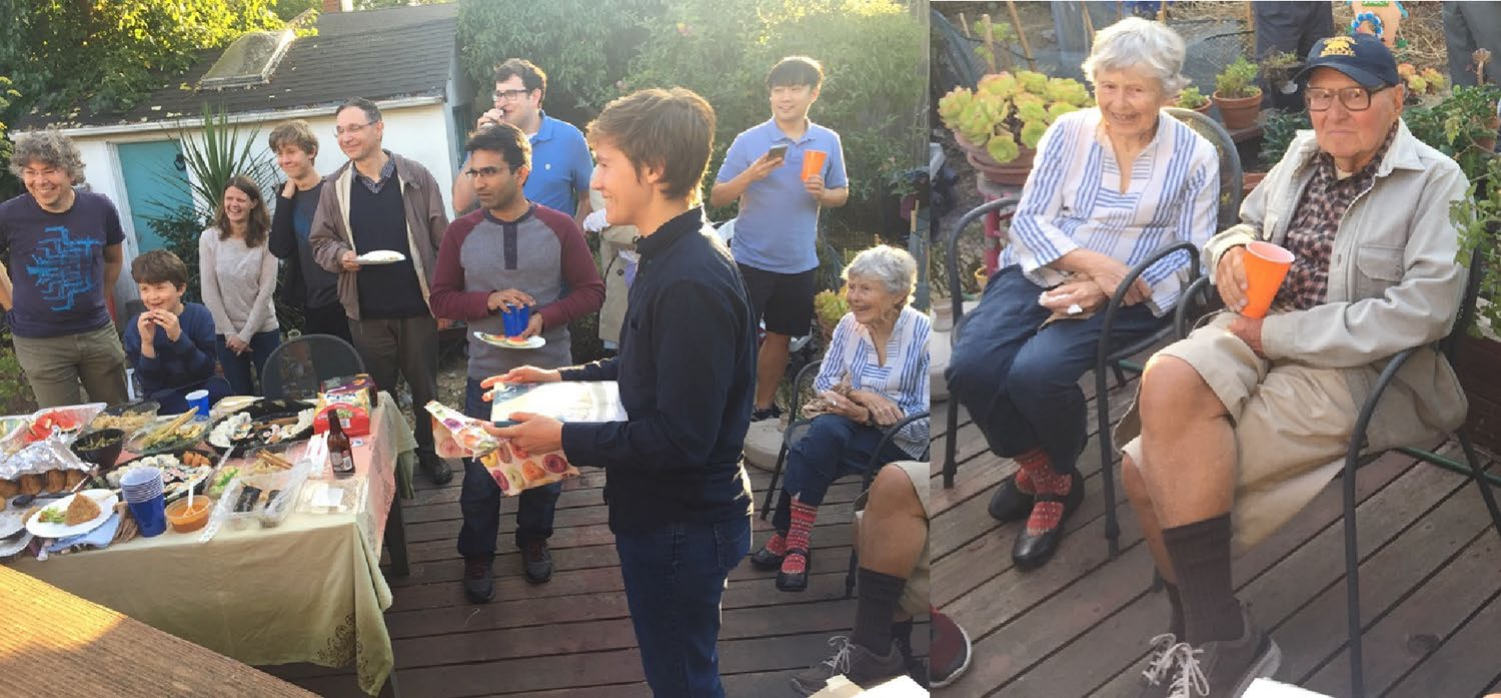
Margie and Ken enjoying the graduation party in 2018 for a group member, Iris Young foreground, now moved to Junko Yano’s garden. Jan Kern on the left with his family (Nils, Silke and David) next to him, Robert Bolotovsky, Sheraz Gul, Kyle Sutherlin, Iris Young, In-Sik Kim. Photos courtesy of Vittal Yachandra

As a physical chemist, Ken brought many aspects of spectroscopy and very diverse interests to the study of photosynthesis. In his group, he created a huge family of people and the space to give people freedom to be themselves and to do their research. Ken has passed on these experiences to us and the next generation, and although the group gatherings will now move to other backyard gardens as seen in the picture here, we three trust that Ken Sauer’s tradition of excellence, mentorship, and community will continue here in our group at Berkeley.

## Supplementary Information

Below is the link to the electronic supplementary material.

Supplementary file1 (DOCX 69 KB)

## Data Availability

No datasets were generated or analysed during the current study.
