# Supplementary material for: Tribute to Kenneth Sauer (1931–2022): a mentor, a role-model, and an inspiration to all in the field of photosynthesis
Source: Photosynth Res. 2024 Nov 13;162(2-3):103–38. doi: 10.1007/s11120-024-01119-0 (PMC11615026; doi:10.1007/s11120-024-01119-0)
Supplement: Supplementary file 1 — Supplementary file1 (DOCX 69 KB) [file 11120_2024_1119_MOESM1_ESM.docx]

**Tribute to Kenneth Sauer (1931-2022) – A mentor, a role-model, and an inspiration to all in the field of photosynthesis**

Junko Yano^1^, Jan Kern^1^, Robert E. Blankenship^2^, Johannes Messinger^3,4^, Vittal K. Yachandra^1^

^1^ Molecular Biophysics and Integrated Bioimaging Division, Lawrence Berkeley National Laboratory, Berkeley, CA 94720, USA

^2^ Washington University in St. Louis, Departments of Biology and Chemistry, St. Louis, MO 63130 USA

^3^ Molecular Biomimetics, Department of Chemistry-Ångström laboratory, Uppsala University, SE 75120 Uppsala, Sweden.

^4^ Umeå Plant Science Centre, Department of Plant Physiology, Umeå University, SE 90187 Umeå, Sweden

**Supplemental Information**

**CURRICULUM VITAE**

**Kenneth Sauer**

**Present Position:**

Professor Emeritus, Department of Chemistry

and Faculty Senior Scientist, Physical Biosciences Division, Lawrence Berkeley

National Laboratory,

University of California, Berkeley, CA 94720.

**Born:** June 19, 1931, Cleveland, Ohio; U.S. Citizen.

**Education:**

Oberlin College, A.B. in Chemistry, 1953.

Harvard University, M.A., 1954; Ph.D. in Physical Chemistry, 1958.

Thesis advisor, Professor George B. Kistiakowsky.

**Professional Career:**

American University of Beirut, Lebanon,

Instructor 1957-58 and Assistant Professor 1958-60 of Chemistry.

University of California, Berkeley,

USPHS Post-doctoral Research fellow, 1960-63.

Assistant Professor of Chemistry, 1963-66.

Associate Professor of Chemistry, 1966-72.

Professor of Chemistry, 1972 - 2001.

Emeritus Professor of Chemistry, 2001-2022.

Vice-Chair for Teaching, Dept. of Chemistry, 1990-2

Associate Dean for Undergraduate Affairs, College of Chemistry, 1995

Faculty Senior Scientist, Calvin Laboratory, Physical Biosciences Division, Lawrence Berkeley National Laboratory, 1963-2022.

Middle-East Technical University, Ankara, Turkey, Visiting Professor, USAID, 1969-70.

Centre d'Études Nucléaires de Saclay, France, 1976-77.

Guggenheim Research Foundation Fellow, 1976-77.

Alexander von Humboldt Foundation, Senior US Scientist Award, 1985-86, 1989 and 1995.

McKnight Foundation Award for Interdisciplinary Science - executive committee (1982-85)

**Membership in Professional Societies:**

American Chemical Society, American Association for the Advancement of Science (Fellow 1976), American Society for Photobiology, American Association of University Professors, Sigma Xi.

**Scientific Interests:**

Photosynthetic energy conversion, electron transport, oxygen evolution, fluorescence lifetimes, electronic excitation transfer studied by optical absorption spectra, circular dichroism, fluorescence depolarization, picosecond kinetics of primary processes, effects of transmembrane electric fields, electron paramagnetic resonance, membrane organization, reaction center structure and function, bacteriochlorophyll protein complexes, phycobiliproteins.

**Professional Honors:**

USAID Visiting Professor Program, 1969-70, Ankara, Turkey.

Japan Society for the Promotion of Science, University Lecturer, March 1976.

Guggenheim Research Foundation Fellow, 1976-77, Saclay, France.

American Association for the Advancement of Science Fellow, 1976.

UNESCO Lecturer, Indian Institute of Science, Bangalore, India, March 1982.

Alexander von Humboldt Foundation, Senior US Scientist Award, 1985-86, 1989 and 1995.

Elected to International Organizing Committee for Photosynthesis, 1989-1995

Research Award, American Society for Photobiology, 1993

Festschrift Issue, Journal of Physical Chemistry B, vol. 102, Issue 42, 1998 Berkeley Citation, University of California, Berkeley, May 2001

**Scientific and Educational Activities:**

Chemistry Department - Lecturing in general chemistry and quantitative analysis (honors level), biophysical chemistry, biomolecular spectroscopy and chemistry for non-science students.

Chair, Curriculum Committee (2002-4)

Member of Planning Committee, Recruiting, Affirmative Action, Curriculum

Revision, Student Advising and Awards, Undergraduate Honors, Research

Conference, Freshman Orientation (CALSO), Graduate Student Admissions

Academic Advising - Undergraduate Majors in College of Chemistry, Chemistry Majors in College of Letters and Science

College of Chemistry - Member of Library Committee, Equipment and Facilities

Committee, TEA(CH)2EM (Chemistry Major plus Teaching Credential Program),

Noyce Prize for Teaching in Science (Chair, 2000), Ombudsperson, advisor to Graduate Student Instructors

Representative to School of Optometry, School of Public Health, College of Natural Resources, University Extension.

University of California - Academic Senate, Representative Assembly

Member-at-Large, Committees on Educational Policy, Courses of Instruction,

Research, Summer Session Task-Force, Group in Biophysics (Executive Committee),

Group in Comparative Biochemistry, Group in Molecular and Physiological Plant

Biology, Sigma Xi Executive Committee, Chancellor’s Committee to Establish a

Program in Biology, Graduate Science Program for Minority Students, France-

Berkeley Research Grants (Chair, 2000-2), Education Abroad Program

Lawrence Hall of Science Advisory Committee, 1993-99.

Textbook writing: I. Tinoco, Jr., K. Sauer, J.C. Wang and J.D. Puglisi, **Physical**

**Chemistry, Principles and Applications in Biological Sciences.** Prentice-Hall , Inc., Englewood Cliffs, NJ 1978. (Second edition, 1985; third edition, 1995; fourth edition 2002).

Editing: **Methods in Enzymology, vol. 246. Biochemical Spectroscopy**. Academic Press, San Diego, CA 1995.

National Science Foundation - Committee on Revising the Undergraduate Curriculum in Science in the U.S. 1989-93

Modular Chemistry Consortium, NSF Program to develop instructional materials for teaching chemistry - founding member and co-author of module on solar energy, 1995-98

Visiting Committee - Carnegie Institution of Washington, Department of Plant Biology (1980-84).

Research Grants Panels - U.S. Department of Agriculture, Competitive Research Grants Office, Photosynthesis Section (1984). U.S. Department of Energy, Biological Energy Research (1986).

Organizing Committee - 7th International Congress of Photosynthesis, Brown University, Providence, RI (August, 1986).

Elected member, International Photosynthesis Committee (1989-95)

Organizing Committee - 2nd and 3rd Berkeley Conferences on Structural Biology, University of California, Berkeley, CA (January 1988 and 1990).

Editorial Boards - Photochemistry and Photobiology, Biophysics of Structure and Mechanism

**Community Activities**

Berkeley Chorus Pro Musica - member 1984-89

San Francisco Bach Choir - member 1990-present

**Invited Lectures and Symposium Presentations (Since Retirement)**

9th Western Photosynthesis Conference, Pacific Grove, CA, January 2000

American Chemical Society National Meeting, San Francisco, CA, March 2000

Gordon Research Conference on Photosynthesis, Henniker, NH, June 2000

13th International Congress on Photobiology, San Francisco, CA, July 2000

12th International Congress on Photosynthesis, Brisbane, Australia, August, 2001

11th Western Photosynthesis Conference, Pacific Grove, CA, January, 2002

International Symposium: Light-Induced Dynamics of Pigment-Proteins and Related Compounds, University of Munich, Germany, June 2002

12th Western Photosynthesis Conference, Pacific Grove, CA, January 2003

Gordon Research Conference on Photosynthesis, Bristol, RI, June 2003

13th Western Photosynthesis Conference, Pacific Grove, CA, January 2004

Sauer, K. Centre d'Etudes Nucleaires-Saclay, Gif-sur-Yvette, France. May 2004.

Sauer, K. Using Solar Energy to Split Water. American Chemical Society, California Section, Berkeley, CA. Sep 2004.

Sauer, K. Mel Klein's Contributions and Impact on the Field of XAS and Photosynthesis. Stanford Synchrotron Radiation Laboratory Users Meeting, Stanford, CA. Oct, 2005.

Sauer, K. Emerging Topics in Photosynthesis - Thinking Outside the Box. 14th Western Photosynthesis Conference, Asilomar, CA. Jan 2006.

Sauer, K. Biomimicry Solutions to Climate Change. Biomimicry Institute and Bioneers, San Rafael, CA. Oct. 2008.

Sauer. K. Plenary Talk. DOE, BES Photosynthetic Systems Research Meeting. Oxygenic Photosynthesis: Past, Present and Future. Annapolis, MD. Oct. 2009.

Sauer, K. Western Photosynthesis Conference, Asilomar, CA. Jan. 2010.

**Students, Postdocs and Associates of Kenneth Sauer**

**Graduate Students**

Joy C. Andrews (Staff Scientist, SSRL, Stanford)

Peter Ang

Sanford A. Asher (Prof., Univ. of Pittsburgh)

Lynn A. Austin

Daniel A. Axelrod

Gerald T. Babcock (Prof., Michigan State, MI)

Dea L. Baumgarten

Mary F. Blackwell (Prof., Lawrence University, MI)

Robert E. Blankenship (Prof., Wash. Univ. in St. Louis, MO)

John D. Bolt

Michael D. Boska

Grace T. Brewington

Ora D. Canaani

John L. Casey (NIH)

Roehl Cinco (DEA, San Francisco)

James L. Cole (Prof., Univ. of Connecticutt, CT)

Joseph N. Dabes

Martin Debreczeny (Merck)

Victoria J. DeRose (Prof., Univ. of Oregon)

David. D. Downie

Edward A. Dratz (Prof., Montana Univ. MT)

James L. Ellenson (Cornell Univ.)

Gerald R. Entine

Richard A. Friesner (Prof., Columbia Univ. NY)

Yvonne M. Gindt (Prof., Lafayette Univ., PA)

David B. Goodin (Prof. Scripps Res. Inst., CA)

Ronald D. Guiles (Prof. Univ. of Maryland, 2/27/10)

Paul R. Hartig

Bruce M. Henkin

Sara M. Hoover

William J. Horsley

Mary H. Kasparian

Jeffrey J. Kelley (Prof. Evergreen State, OR)

David Kreller

Matthew Latimer (Scientist, SSRL, Stanford)

Wenchuan Liang (Stanford Univ.)

Robert M. Macnab (Prof., Yale, Univ. CT)

Patricia Maxson (Merck)

John L. McCracken (Prof., Mich. State Univ., MI)

Ann E. McDermott (Prof., Columbia Univ., NY)

Daniel M. Michaelson

Ishita Mukerji (Prof., Wesleyan Univ., CT)

John A. Nairn

Gary T. Olsen

Kenneth D. Phillipson (Prof., UCLA School of Medicine, CA)

Shelly Pizarro (Genentech, San Francisco)

Johm Robblee (Momenta Corp, Boston)

Alfred J. Schultz, Jr.

David D. Sherertz

Alexander Shih-Kaung Sun

Jana Steiger

Steven C. Switalski

Sarah Tabbutt (Pediatrician)

Mary Talbot

Nelson A. Teng

George D. Vaughan

Henk Visser

Stephen T. Worland (Aguron Corp.)

James E. Wurzbach

Esther K. Yang

Muh-ching Yee

**Postdoctoral Associates**

Ruth G. Alscher

Joy Andrews

Warren Beck (Prof., Michigan State, MI)

Emmanuele Bellacchio

Neil V. Blough (Woods Hole)

David Britt, Ph.D. (Prof. Univ. of California, Davis, CA)

Gary W. Brudvig (Prof., Yale Univ., CT)

Silvia M. de Brito Costa

Lelia Coyne

Holger Dau (Prof., Free Univ. Berlin, Germany)

Victoria J. DeRose (Prof., Univ. of Oregon, Eugene, OR)

G. Charles Dismukes (Prof., Princeton, and now at Rutgers Univ. NJ).

David Ellett

Carmen Fernandez (Prof., Univ. of Sao Paolo, Brazil)

Harry A. Frank (Prof., Univ. of Connecticutt, CT)

David Gill

Jeffrey Gingrich (Joint Genome Institute, Walnut Creek)

Wolfgang Haehnel (Prof. Univ. of Freiburg, Germany)

Philip Haworth

Alan R. Horwitz

Claude Houssier

Kerry K. Karukstis (Prof., Harvey Mudd College)

Hermann Keuper

Jan Kern (Senior Scientist, LBNL)

Melvin A. Kronick

Matthew Latimer (SSRL. Stanford

Karen McFarlane-Holman (Prof., Willammette Univ., Salem, OR)

Johannes Messinger (Prof., Umea University, Sweden)

Paul Mathis (CNRS, Saclay)

Tsunenori Nozawa (Univ. of Osaka Prefecture, Japan)

Yulia Pushkar (Prof., Purdue Univ., IN)

Paul Reisberg

Theo Roelofs (Charite Hospital, Berlin)

Annette F. Rompel (Prof., Univ. of Vienna, Austria)

Vicki L. Sato

Kirk S. Schanze (Prof., Univ. of Florida)

Shirley C. Tsai

Larry E. Vickery (Prof., Univ. of California, Irvine, CA)

Joseph T. Warden (Prof., Rensselaer Polytechnic Institute, NY)

Thomas W. Wydrzynski (Prof., Australian National Univ., Canberra, Australia)

Vittal K. Yachandra (Senior Scientist, LBNL, Berkeley)

Akihiko Yamagishi (Tokyo Univ., Japan)

Xin-wei Yan

Junko Yano (Director, MBIB Division, LBNL. Berkeley)

Jean-Luc Zimmermann (CNRS, France)

**Sabbatical Visitors**

John Allen

Elodie Anxolabehere-Mallart

Salil Bose

Beverley R. Green

Alfred Holzwarth

Masami Kusunoki

Joon Woo Park

Robert T. Ross

Kenneth Rousslang

Hugo Scheer

Dietmar Stehlik

**Publications**

**Kenneth Sauer**

1. G.B. Kistiakowsky and K. Sauer. The Rate and Mechanism of Some Reactions of Methylene. *J. Am. Chem. Soc*. **78**, 5699 (1956).

1. G.B. Kistiakowsky and K. Sauer. Reactions of Methylene. 2. Ketene and Carbon Dioxide. *J. Am. Chem. Soc*. **80**, 1066-1071 (1958).

1. K. Sauer and M. H. Kasparian. Kinetics of the Reaction of Phenyl Isocyanate with Aniline. *J. Org. Chem*. **26**, 3498-3504 (1958).

1. A. Kilejian, K. Sauer and C. W. Schwabe. Host-Parasite Relationships in Echinococcosis. VIII. Infrared Spectra and Chemical Composition of the Hydatid Cyst. *Exper. Parasitol*. **12**, 377-392 (1962).

1. K. Sauer and M. Calvin. Molecular Orientation in Quantasomes. I. Electric Dichroism and Electric Birefringence of Quantasomes from Spinach Chloroplasts. *J. Mol. Bio*l. **4**, 451-466 (1962).

1. K. Sauer and M. Calvin. Absorption Spectra of Spinach Quantasomes and Bleaching of the Pigments. *Biochim . Biophys. Acta* 64,

1. K. Sauer and R.B. Park. Molecular Orientation in Quantasomes. II. Absorption Spectra, Hill Activity and Fluorescence Yields. *Biochim. Biophys. Acta*. **79**, 476-489 (1964).

1. J. Biggins and K. Sauer. Action Spectrum of the Hill Reaction with Ferricyanide and Ferricyanide/Indophenol by Isolated Chloroplasts. *Biochim. Biophys. Acta* **88**, 655-657 (1964).

1. K. Sauer. Molecular Orientation in Quantasomes. III. A Flow Dichroism Apparatus and Its Application to the Study of the Structure of Spinach Quantasomes. *Biophysical J*. **5**, 337-348 (1965).

1. K. Sauer and J. Biggins. The Action Spectrum and Quantum Yields for Nicotinamide Adenine Dinucleotide Phosphate Reduction by Chloroplasts. *Biochim. Biophys. Acta* **102**, 55-72 (1965).

1. K. Sauer. Optical Rotatory Dispersion of Chlorophyll in Solution and in Chloroplast Subunits. Proc. Natl. Acad. Sci. USA **53**, 716-722 (1965).

1. K. Sauer and R.B. Park. The Hill Reaction of Chloroplasts. Action Spectra and Quantum Requirements. *Biochemistry* **4**, 2791-2797 (1965).

1. J. Kelly and K. Sauer. Action Spectrum and Quantum Requirements for the Photoreduction of Cytochrome c with Spinach Chloroplasts. *Biochemistry* **4**, 2798-2802 (1965).

1. R.B. Park, J. Kelly, S. Drury and K. Sauer. The Hill Reaction of Chloroplasts Isolated from Glutaraldehyde Fixed Spinach Leaves. Proc. Natl. Acad. Sci. USA **55**, 1056-1062 (1966).

1. K. Sauer, J.R. Lindsay Smith and A.J. Schultz. The Dimerization of Chlorophyll a, Chlorophyll b and Bacteriochlorophyll in Solution.  *J. Am. Chem. Soc.* **88**, 2681-2688 (1966).

1. E.A. Dratz, A.J. Schultz and K. Sauer. Chlorophyll-Chlorophyll Interactions. *Brookhaven Symposia in Biology* **19**, 303-318 (1966).

1. J. Kelly and K. Sauer. Functional Photosynthetic Unit Sizes for Each of the Two Light Reactions in Spinach Chloroplasts. *Biochemistry* **7**, 882-890 (1968).

1. T. Trosper, R.B. Park and K. Sauer. Excitation Transfer by Chlorophyll a in Monolayers and the Interaction with Chloroplast Glycolipids. *Photochem.* *Photobiol*. **7**, 451-469 (1968).

1. T. Trosper and K. Sauer. Chlorophyll a Interactions with Chloroplast Lipids in Vitro. *Biochim. Biophys. Acta*. **162**, 97-105 (1968).

1. K. Sauer, E. A. Dratz and L. Coyne. Circular Dichroism Spectra and the Molecular Arrangement of Bacteriochorophyll in the Reaction Centers of Photosynthetic Bacteria. *Proc. Natl. Acad. Sci. USA* **61**, 17-24 (1968).

1. C. Houssier and K. Sauer. Optical Properties of the Protochlorophyll Pigments. I. Isolation, Characterization, and Infrared Spectra. *Biochim. Biophys. Acta* **172**, 476-491 (1969).

1. C. Houssier and K. Sauer. Optical Properties of the Protochlorophyll Pigments.

II. Electronic Absorption, Fluorescence and Circular Dichroism Spectra. *Biochim*. *Biophys. Acta* **172**, 492-502 (1969).

1. K. Sauer and E.A. Dratz. Pigment Antennas and Reaction Centers in Photosynthesis. *Progress in Photosynthesis Research* (H. Metzner, Ed.), Vol. II, H. Laupp, Tübingen, Germany, 1969, pp. 837-844.

1. C. Houssier and K. Sauer. Circular Dichroism and Magnetic Circular Dichroism of the Chlorophyll and Protochlorophyll Pigments. *J. Am. Chem. Soc.* **92**, 779791 (1970).

1. C. Weiss, Jr. and K. Sauer. Activation Kinetics of Photosynthetic Oxygen Evolution under 20-40 Nanosecond Laser Flashes. *Photochem. Photobiol.* **11**, 495-501 (1970).

1. R.M. Macnab and K. Sauer. Electronic Absorption and Fluorescence Spectra of Anthracene, [D_10_]-Anthracene and Acridine in n-Alkane Matrices at 4°K. *J.* *Chem. Phys*. **53**, 2805-2817 (1970).

1. S. Süzer and K. Sauer. The Sites of Photoconversion of Protochlorophyllide to Chlorophyllide in Barley Seedlings. *Plant Physiol*. **48**, 60-63 (1971).

1. K.D. Philipson, S.C. Tsai and K. Sauer. Circular Dichroism of Chlorophyll and Related Molecules Calculated Using a Point Monopole Model for the Electronic Transitions. *J. Phys. Chem.* **75**, 1440-1445 (1971).

1. A.S.K. Sun and K. Sauer. Pigment Systems and Electron Transport in Chloroplasts. I. Quantum Requirements for the Two Light Reactions in Spinach Chloroplasts.  *Biochim. Biophys. Acta* **234**, 399-414 (1971).

1. A.S.K. Sun and K. Sauer. Pigment Systems and Electron Transport in Chlorplasts. II. Emerson Enhancement in Broken Spinach Chloroplasts. *Biochim*. *Biophys. Acta* **256**, 409-427 (1972).

1. K. Sauer. Circular Dichroism and Optical Rotatory Dispersion of Photosynthetic Organelles and Their Component Pigments. Photosynthesis and Nitrogen Fixation (S. San Pietro, Ed.) *Methods in Enzymology*, Vol. **24**, 206-217 (1972).

1. K.D. Philipson and K, Sauer. Exciton Interaction in a Bacteriochlorophyll-protein from Chloropseudomonas ethylica: Absorption and Circular Dichroism at 77K. *Biochemistry* **11**, 1880-1885 (1972).

1. A. Schultz and K. Sauer. Circular Dichroism and Fluorescence Changes Accompanying the Protochlorophyllide to Cholorphyllide Transformation in Greening Leaves and Holochrome Preparations. *Biochim. Biophys. Acta* **267**, 320340 (1972).

1. P. Mathis and K. Sauer. Circular Dichroism Studies on the Structure and the Photochemistry of Protochlorophyllide and Chlorophyllide Holochrome. *Biochim*. *Biophys. Acta* **267**, 498-511 (1972).

1. K.D. Philipson and K. Sauer. Exciton Interaction in the Photosystem I Reaction Center from Spinach Chloroplasts. Absorption and Circular Dichroism Difference Spectra. *Biochemistry* **11**, 4591-4595 (1972).

1. J.M. Olson, K.D. Philipson and K. Sauer. Circular Dichroism and Absorption Spectra of Bacteriochlorophyll-Protein and Reaction Center complexes from Chlorobium thiosulfatophilum. *Biochim. Biophys. Acta* **292**, 206-217 (1973).

1. P. Mathis and K. Sauer. Chlorophyll Formation in Greening Bean Leaves during the Early Stages. *Plant Physiol*. **51**, 115-119 (1973).

1. K.D. Philipson and K. Sauer. Comparative Study of the Circular Dichroism Spectra of Reaction Centers from Several Photosynthetic Bacteria. *Biochemistry* **12**, 535-539 (1973).

1. K.D. Philipson and K. Sauer. Light-Scattering Effects on the Circular Dichroism of Chloroplasts. *Biochemistry* **12**, 3454-3458 (1973).

1. G.T. Babcock and K. Sauer. Electron Paramagnetic Resonance Signal II in Spinach Chloroplasts. I. Kinetic Analysis for Untreated Chloroplasts. *Biochim*. *Biophys. Acta* **325**, 483-503 (1973).

1. G.T. Babcock and K. Sauer. Electron Paramagnetic Resonance Signal II in Spinach Chloroplasts. II. Alternative Forms and Inhibitor Effects on Kinetics of Signal II in Flashing Light. *Biochim. Biophys. Acta* **325**, 504-519 (1973).

1. G.D. Vaughan and K. Sauer. Energy Transfer friom Protochlorophyllide to Chlorophyllide during Photoconversion of Etiolated Bean Holochrome. *Biochim*. *Biophys. Acta* **347**, 383-394.

1. R.E. Blankenship and K. Sauer. Manganese in Photosynthetic Oxygen Evolution. I. Electron Paramagnetic Resonance Study of the Environment of Manganese in Tris-Washed Chloroplasts. *Biochim. Biophys. Acta* **376**, 315-328 (1975).

1. G.T. Babcock and K. Sauer. A Rapid, Light-Induced Transient in Electron Paramagnetic Resonance Signal II Activated upon Inhibition of Photosynthetic Oxygen Evolution. *Biochim. Biophys. Acta* **376**, 315-328, (1975).

1. G.T. Babcock and K. Sauer. The Rapid Compnent of Electron Paramagnetic Resonance Signal II: A Candidate for the Physiological Donor to Photosystem II in Spinach Chloroplasts. *Biochim. Biophys. Acta* **376**, 329-344 (1975).

1. K. Sauer. Primary Events and the Trapping of Energy. **In** Bioenergetics of Photosynthesis. (Govindjee, Ed.) Chap. 3, Academic Press, New York, 1975, pp. 115-181.

1. R.E. Blankenship, G.T. Babcock,, J..T. Warden and K. Sauer. Observation of a New EPR Transient in Chloroplasts that May Reflect the Electron Donor to Photosystem II at Room Temperature. *FEBS Letts*. **51**, 287-293 (1975).

1. L. Vickery, A. Salmon and K. Sauer. Magnetic Circular Dichroism Studies on Microsomal Aryl Hydrocarbon Hydroxylase: Comparison with Cytochrome b5 and Cytochrome P-450cam. *Biochim. Biophys. Acta* **386**, 87-89 (1975)

1. R.E. Blankenship, G.T. Babcock and K. Sauer. Kinetic Study of Oxygen Evolution Parameters in Tris-Washed, Reactivated Chloroplasts. *Biochim*. *Biophys. Acta* **387**, 165-175 (1975).

1. A.C. Ley. G.T. Babcock and K. Sauer. Flash Kinetics and Light Intensity Dependence of Oxygen Evolution in the Blue-Green Alga Anacystis nidulans. *Biochim. Biophys. Acta* **387**, 379-387 (1975).

1. G.T. Babcock and K. Sauer. Two Electron Donation Sites for Exogenous Reductants in Chloroplast Photosystem II. *Biochim. Biophys. Acta* **396**, 48-62 (1975).

1. R. Blankenship, A. McGuire and K. Sauer. Chemically Induced Dynamic Electron Polarization (CIDEP) in Chloroplasts at Room Temperature; Evidence for Triplet State Participation in Photosynthesis. *Proc. Natl. Acad. Sci. USA* **72**, 4943-4947 (1975).

1. L. Vickery, R. Nozawa and K. Sauer. Magnetic Circular Dichroism Studies of Myoglobin Complexes. Correlation with Heme Spin State and Axial Ligation. *J. Amer. Chem. Soc*. **98**, 343-350 (1976).

1. L. Vickery, T. Nozawa and K. Sauer. Magnetic Circular Dichroism of Low Spin Cytochromes. Temperature Dependence and Effects of Axial Coordination on the Spectra of Cytochrome c and Cytochrome b5. *J. Am. Chem Soc*. **98**, 351-357 (1976).

1. G.T. Babcock, R.E. Blankenship and K. Sauer. Reaction Kinetics for Positive Charge Accumulation on the Water Side of Chloroplast Photosystem II. *FEBS* *Letts*. **61**, 286-289 (1976).

1. J.T. Warden, R.E. Blankenship and K. Sauer. A Flash Photolysis ESR Study of Photosystem II Signal IIvf, the Physiological Donor to P-680^+^. *Biochim. Biophys. Acta* **423**, 462-478 (1976).

1. J. L. Ellenson and K. Sauer. The Electrophotoluminescence of Chloroplasts. *Photochem. Photobiol*. **23**, 113-123 (1976).

1. S. Asher and K. Sauer. Resonance Raman Spectroscopy of Mn (III) Etioporphyrin I at the π−π* and Charge Transfer Bands: The Use of Charge Transfer Bands to Monitor the Complexation State of Metallo-porphyrins. *J. Chem. Phys*. **64**, 4115-4125 (1976).

1. B. Leskovar, C.C. Lo, P.R. Hartig and K. Sauer. Photon Counting System for Subnanosecond Fluorescence Lifetime Measurements. *Rev. Sci. Instr*. **47**, 11131121 (1976).

1. P.R. Hartig, K. Sauer, C.C. Lo and B. Leskovar. Measurement of Very Short Fluorescence Lifetimes by Single-Photon Counting. *Rev. Sci. Instr*. **47**, 1122-1129 (1976).

1. R.G. Alscher, S.P. Hawkes and K. Sauer. The Association of Protein Synthesis with Protochlorophyllide Holochrome Regeneration in Dark-Grown Barley Leaves. *Biochem. Biophys. Res. Communs*. **73**, 240-247 (1976).

1. R.E. Blankenship, A. McGuire and K. Sauer. Rise Time of EPR Signal IIyf in Chloroplast Photosystem II. *Biochim. Biophys. Acta* **459**, 617-619 (1977).

1. B. M. Henkin and K. Sauer. Magnesium Ion Effects on Chloroplast Photosystem II Fluorescence and Photochemistry. *Photochem. Photobio*l. **26**, 277-286 (1977).

1. O.D. Canaani and K. Sauer. Analysis of the Subunit Structure of Protochlorophyllide Holochrome by Sodium Dodecyl Sulfate-Polyacrylamide Gel Electrophoresis. *Plant Physiol*. **60**, 422-429 (1977).

1. P.R. Hartig, N.J. Bertrand and K. Sauer. 5-Iodoacetamidofluorescein-Labeled Chloroplast Coupling Factor I: Conformational Dynamics and Labeling Site Characterization. *Biochemistry* **16**, 4275-4282 (1977).

1. S.A. Asher, L.E. Vickery, T.M. Schuster and K. Sauer. Resonance Raman Spectra of Methemoglobin Derivatives. Selective Enhancement of Axial Ligand Vibrations and Lack of an Effect of Inositol Hexaphosphate. *Biochemistry* **16**, 5849-5856 (1977).

1. K. Sauer, S. Acker, P. Mathis and J.A. Van Best. Optical Studies of Photosystem

I Particles: Evidence for the Presence of Multiple Electron Acceptors. In Bioenergetics of Membranes (L. Packer, et al., Eds.) Elsevier/North Holland, 1977, pp. 351-359.

1. K. Sauer and G.T. Brewington. Fluorescence Lifetimes of Chloroplasts, Subchloroplast Particles and Chorella Using Single Photon Counting. Proc. 4th Intl. Congr. Photosynth., Biochem. Soc., Publ. London, 1978, pp. 409-421.

1. G.C. Dismukes, A. McGuire, R. Blankenship and K. Sauer. Electron Spin Polarization in Photosynthesis and the Mechanism of Electron Transfer in Photosystem I: Experimental Observations.  *Biophys. J*. **21**, 239-256; **22**, 521 (1978).

1. O.D. Canaani and K. Sauer. Absorption and Circular Dichroism Spectra of Chloroplast Membrane Fragments from Spinach, Barley and a Barley Mutant at Room Temperature and Liquid Nitrogen Temperature. *Biochim. Biophys. Acta* **501**, 545-551 (1978).

1. P. Mathis, K. Sauer and R. Remy. Rapidly Reversible Flash-Induced Electron Transfer in a P-700 Chlorophyll-Protein Complex Isolated with SDS. *FEBS Letts*. **88**, 275-278 (1978).

1. K. Sauer and L.A. Austin. Bacteriochlorophyll-Protein Complexes from the Light-Harvesting Antenna of Photosynthetic Bacteria. *Biochemistry* **17**, 20112019 (1978).

1. K. Sauer, P. Mathis, S. Acker and J.A. Van Best. Electron Acceptors Associated with P-700 in Triton Solubilized Photsystem I Particles from Spinach Chloroplast. *Biochim. Biophys. Acta* **503**, 120-134 (1978).

1. K. Sauer. Photosynthetic Membranes. *Accts. Chem. Res*. **11**, 257-264 (1978).

1. G.C. Dismukes and K. Sauer. The Orientation of Membrane Bound Radicals. An EPR Investigation of Magnetically Ordered Spinach Chloroplasts. *Biochim*. *Biophys. Acta* **504**, 431-445 (1978).

1. K. Sauer, P, Mathis, S. Acker, and J.A. Van Best. Absorption Changes of P-700 Reversible in Milliseconds at Low Temperature in Triton-Solubilized Photosystem I Particles. *Biochim. Biophys. Acta* **545**, 466-472 (1979).

1. J. Bolt and K. Sauer. Linear Dichroism of Light Harvesting Bacteriochlorophyll Proteins from Rhodopseudomonas sphaeroides in Stretched Polyvinyl Alcohol Films. *Biochim. Biophys. Acta* **546**, 54-63 (1979).

1. R. Friesner, G.C. Dismukes, and K. Sauer. Development of Electron Spin Polarization in Photosynthetic Electron Transfer by the Radical Pair Mechanism. *Biophys. J*. **25**, 277-294 (1979).

1. G.C. Dismukes, A. McGuire, R. Friesner, and K. Sauer. Electron Spin Polarization from the Photosynthetic Light Reaction: Photosystem I. *Reviews of Chemical Intermediates* **3,** 59-88 (1979).

1. H.A. Frank, R. Friesner, J.A. Nairn, G.C. Dismukes, and K. Sauer. The Orientation of the Primary Donor in Bacterial Photosynthesis.  *Biochim. Biophys.* *Acta* **547**, 484-501 (1979).

1. H.A. Frank, J. Bolt, R. Friesner, and K. Sauer. Magnetophotoselection of the Triplet States of Reaction Centers from Rhodopseudomonas sphaeroides R-26. *Biochim. Biophys. Acta* **547**, 502-511 (1979).

1. K. Sauer. Photosynthesis - The Light Reactions. *Annu. Rev. Phys. Chem*. **30**, 155-178 (1979).

1. R. Friesner, J.A. Nairn, and K. Sauer. Direct Calculation of the Orientational Distribution Function of Partially Ordered Ensembles from the EPR Lineshape.  *J*. *Chem. Phys***. 71**, 358-365 (1979).

1. C.N. Rafferty, J. Bolt, K. Sauer, and R.K. Clayton. Photooxidation of Antenna Bacteriochlorophyll in Chromatophores from Carotenoidless Mutant Rhodopseudomonas sphaeroides and the Attendant Loss of Dimeric Exciton Interaction. *Proc. Natl. Acad. Sci. USA* **76**, 4429-4432 (1979).

1. H.A. Frank, M.B. McLean, and K. Sauer. Triplet States in Photosystem I of Chloroplasts and Subchloroplast Particles. *Proc. Natl. Acad. Sci. USA* **76**, 51245128 (1979).

1. T. Wydrzynski and K. Sauer. Periodic Changes in the Oxidation State of Manganese in Photosynthetic Oxygen Evolution upon Illumination with Flashes. *Biochim. Biophys. Acta*  **589**, 56-70 (1980).

1. R. Friesner, J.A. Nairn, and K. Sauer. A General Theory of the Spectroscopic Properties of Partially Ordered Ensembles. I. One Vector Problems. *J. Chem.* *Phys*. **72**, 221-230 (1980).

1. K. Sauer. A Role for Manganese in Oxygen Evolution in Photosynthesis. Accts. Chem. Res. **13**, 249-256 (1980).

1. H.A. Frank, J.D. Bolt, S.M. de B. Costa, and K. Sauer. Electron Paramagnetic Resonance Detection of Carotenoid Triplet States. *J. Am. Chem. Soc.* **102**, 48934898 (1980).

1. J.A. Nairn, R. Friesner, H.A. Frank, and K. Sauer. A New Approach to the Theory of Linear Dichroism in Partially Ordered Systems: Application to Reaction Centers and Whole Cells of Photosynthetic Bacteria. *Biophys*. *J*.**32**, 733-753 (1980).

1. K. Sauer. Charge Separation in the Light Reactions of Photosynthesis. **In:** Photosynthesis III. Structure and Molecular Organization of the Photosynthetic Apparatus (G. Akoyunoglou, Ed.). Balaban International Science Service, Philadelphia, Pa, (1981) pp. 685-699.

1. J.A. Nairn, R. Friesner, and K. Sauer. A General Theory of the Spectroscopic Properties of Partially Ordered Ensembles II. Two-Vector Problems. *J. Chem.* *Phys*. **74**, 5398-5406 (1981).

1. R. Friesner, J.L. McCracken, and K. Sauer. Transient Solutions of the Bloch Equations for Inhomogeneously Broadened Lines. *J. Magnet. Res*. **43**, 343-356

(1981).

1. J.D. Bolt, K. Sauer, J.A. Shiozawa, and G. Drews. Linear and Circular Dichroism of Membranes from Rhodopseudomonas capsulata.. *Biochim*. *Biophys. Acta* **635**, 535-541 (1981).

1. J.D. Bolt, C.N. Hunter, R.A. Niederman, and K. Sauer. Linear and Circular Dichroism and Fluorescence Polarization of the B875 Light-Harvesting Bacteriochlorophyll-Protein Complex from Rhodopseudomonas sphaeroides. *Photochem. Photobiol*. **34**, 653-656 (1981).

a. J.D. Bolt, C.N. Hunter, R.A. Niederman, and K. Sauer. Light-harvesting

Pigments in Photosynthetic Bacteria. *BioScience* **31**, 525-526 (1981).

1. J.D. Bolt and K. Sauer. Fluorescence Properties of the Light-Harvesting Bacteriochlorophyll Protein from Rhodopseudomonas sphaeroides R-26. *Biochim. Biophys. Acta* **637**, 342-347 (1981).

1. W. Haehnel, J.A. Nairn, and K. Sauer. Fluorescence Lifetimes of *i*n Vivo Chlorophyll Studied in Chloroplasts and Algae. *J. Luminescence* **24/25**, 795-798 (1981).

1. E.K. Yang and K. Sauer. Correlation between the Optical and Magnetic Properties of Ferric N-Acetylated Heme Octapeptide Complexes. **In:** Electron Transport and Oxygen Utilization (C. Ho, Ed.), Elsevier North Holland, Inc., Amsterdam, (1982) pp. 89-94.

1. J.L. McCracken, H.A. Frank, and K. Sauer. Radical Pair Interactions in Spinach Chloroplasts. *Biochim. Biophys. Acta* **679**, 156-168 (1982).

1. M.B. McLean and K. Sauer. The Dependence of Reaction Center and Antenna Triplets on the Redox State of Photosystem I. *Biochim. Biophys. Acta* **679**, 384392 (1982).

1. W. Haehnel, J.A. Nairn, P. Reisberg, and K. Sauer. Picosecond Fluorescence Kinetics and Energy Transfer in Chloroplasts and Algae. *Biochim. Biophys. Acta*, **680**, 161-173 (1982).

1. P. Reisberg, J.A. Nairn, and K. Sauer. Picosecond Fluorescence Kinetics in Spinach Chloroplasts at Low Temperature. *Photochem. Photobiol.* **36**, 657-661 (1982).

1. J.A. Nairn, W. Haehnel, P. Reisburg, and K. Sauer. Picosecond Fluorescence Kinetics in Spinach Chloroplasts at Room Temperature: Effects of Magnesium Ion. *Biochim. Biophys. Acta* **682**, 420-429 (1982).

1. K. Sauer. Charge Separation in Photosynthetic Light Reactions.  *Chemica Scripta* **21**, 65 (1982).

1. G.W. Brudvig, S.T. Worland, and K. Sauer. Procedure for Rapid Isolation of Photosynthetic Reaction Centers Using Cytochrome c Affinity Chromatography. Proc. Natl. Acad. Sci. USA **80**, 683-686 (1983).

1. B.T. Turko, J.A. Nairn, and K. Sauer. Single Photon Timing System for Picosecond Fluorescence Lifetime Measurements. Rev. Sci. Instrum. **54**, 118-120 (1983).

1. M. Boska, K. Sauer, W. Buttner, and G.T. Babcock. Similarity of EPR Signal II_f_

Rise and P680+ Decay Kinetics in Tris-Washed Chloroplast Photosystem II

Preparations as a Function of pH. *Biochim. Biophys. Acta* **722,** 327-330 (1983).

1. K.K. Karukstis and K. Sauer. Potentiometric Titration of Photosystem II Fluorescence Decay Kinetics in Spinach Chloroplasts. *Biochim. Biophys. Acta* **722,** 364-371 (1983).

1. G.W. Brudvig, J.L. Casey, and K. Sauer. The Effect of Temperature on the Formation and Decay of the Multiline EPR Signal Species Associated with

Photosynthetic Oxygen Evolution.  *Biochim. Biophys. Acta* **723**, 366-371 (1983)

a. Sauer and J.L. Casey. Photosynthetic Oxidation of Water to Dioxygen in

Chloroplast Membranes (Abstract). *J. Electrochem. Soc*. **130**, 117C-118C (1983).

1. J.L. McCracken and K. Sauer. Orientation Dependence of Radical Pair Interactions in Spinach Chloroplasts. *Biochim. Biophys. Acta* **724**, 83-93 (1983).

1. K.K. Karukstis and K. Sauer. Picosecond Fluorescence Kinetic Studies of Electron Acceptor Q Redox Heterogeneity. *Biochim. Biophys. Acta* **725,** 246-253 (1983).

1. P. Haworth, K.K. Karukstis, and K. Sauer. Picosecond Fluorescence Kinetics in Spinach Chloroplasts at Room Temperature: Effects of Phosphorylation. *Biochim*. *Biophys. Acta* **725**, 261-271 (1983).

1. K.K. Karukstis and K. Sauer. Photosynthetic Membrane Development Studied Using Picosecond Fluorescence Kinetics. *Biochim. Biophys. Acta* **725**, 384-393 (1983).

1. G.W. Brudvig, J.L. Casey, and K. Sauer. Properties of the S_2_ State Associated with O_2_ Evolution. **In** The Oxygen-Evolving System of Photosynthesis, (Y. Inoue, Ed.). Academic Press Japan, Tokyo, (1983) pp. 159-164.

1. K.K. Karukstis and K. Sauer. Fluorescence Decay Kinetics of Chlorophyll in Photosynthetic Membranes. *J. Cellular Biochem*. **23**, 131-158 (1983).

a. K.K. Karukstis and K. Sauer. Fluorescence Decay Kinetics of Chlorophyll in

Photosynthetic Membranes. **In**: Biosynthesis of the Photosynthetic Apparatus:

Molecular Biology, Development and Regulation. (J.P. Thornber,L.A. Staehelin, R.B. Hallick, Eds.) UCLA Symposia on Molecular and Cellular Biology, vol. **14**,

A.R. Liss Inc., N.Y., (1984) pp 59-86.

1. J.L. McCracken and K. Sauer. Electron Paramagnetic Resonance Studies of the Primary Electron Acceptors of Photosystem I. **In** Advances in Photosynthesis Research (C. Sybesma, Ed.). M. Nijhoff/Dr. W. Junk, Publ., The Hague, (1984) vol I.4, pp. 585-588.

1. K. Sauer, M. Boska, J.L. Casey, and K.K. Karukstis. Photosystem 2 and Water Oxidation in Higher Plants. **In** Advances in Photosynthesis Research (C. Sybesma, Ed.). M. Nijhoff/Dr. W. Junk, Publ., The Hague, (1984) vol I.2, pp. 121-126.

1. J.E. Hearst and K. Sauer. Protein Sequence Homologies between Portions of the L and M Subunits of Reaction Centers of Rhodopseudomonas capsulata and the 32 kD Herbicide-Binding Polypeptide of Chloroplast Thylakoid Membranes and a

Proposed Relation to Quinone-Binding Sites **In** Advances in Photosynthesis Research (C. Sybesma, Ed.). M. Nijhoff/Dr. W. Junk, Publ., The Hague, (1984) vol III.5, pp. 355-359.

1. G.C. Dismukes, H.A. Frank, R. Friesner, and K. Sauer. Electronic Interactions between Iron and Bound Semiquinones in Bacterial Photosynthesis: EPR Spectroscopy of Oriented Cells of Rhodopseudomonas viridis. *Biochim. Biophys*. *Acta* **64**, 253-271 (1984).

1. J.E. Hearst and K. Sauer. Protein Sequence Homologies between Portions of the L and M Subunits of Reaction Centers of Rhodopseudomonas capsulata and the Q_B_ Protein of Chloroplast Thylakoid Membranes: A Proposed Relation to Quinone-Binding Sites. Zeitschrift für Naturforschung **39c**, 421-424 (1984).

1. M. Boska and K. Sauer. Kinetics of EPR Signal II_f_ in Chloroplast Photosystem II. *Biochim. Biophys. Acta* **765**, 84-87 (1984).

1. S.C. Switalski and K. Sauer. Energy Transfer among the Chromophores of C-Phycocyanin from Anabaena variabilis using Steady State and Time-Resolved Fluorescence Spectroscopy.  *Photochem. Photobiol*. **40**, 423-427 (1984).

1. Karukstis and K. Sauer. Energy Transfer and Distribution in the Red Alga Porphyra perforata Studied Using Fluorescence Spectroscopy. *Biochim. Biophys. Acta* **766**, 141-147 (1984).

1. K.K. Karukstis and K. Sauer. Organization of the Photosynthetic Apparatus of the chlorina-f2 Mutant of Barley Using Chlorophyll Fluorescence Decay Kinetics. *Biochim. Biophys. Acta* **766**, 148-155 (1984).

1. J.L. Casey and K. Sauer. EPR Detection of a Cryogenically Photogenerated Intermediate in Photosynthetic Oxygen Evolution. *Biochim. Biophys. Acta* **767**, 21-28 (1984).

1. N.V. Blough and K. Sauer. The Effects of Mono- and Divalent Salts on the O2 Evolution Activity and Low Temperature Multiline EPR Spectrum of

Photosystem II Preparations from Spinach. *Biochim. Biophys. Acta* **767**, 377-381 (1984)

1. D.B. Goodin, V.K. Yachandra, R.D. Britt, K. Sauer, and M.P. Klein. The State of Manganese in the Photosynthetic Apparatus 3. Light-Induced Changes in X-ray Absorption (K-Edge) Energies of Manganese in Photosynthetic Membranes. *Biochim. Biophys. Acta* **767**, 209-216 (1984)

1. B.R. Green, K.K. Karukstis, and K. Sauer. Fluorescence Decay Kinetics of Mutants of Corn Deficient in Photosystem I and Photosystem II. *Biochim*. *Biophys. Acta* **767**, 574-581 (1984).

1. S.T. Worland, K.J. Wilson, J.E. Hearst, and K. Sauer. Isolation and AminoTerminal Sequences of Subunits from the Photosynthetic Reaction Center of Rhodopseudomonas capsulata. *Biochim. Biophys. Acta*, **767**, 651-654 (1984).

1. D.B.Goodin, V.K. Yachandra, R. Guiles, R.D. Britt, A. McDermott, K. Sauer, and M.P. Klein. Light-Induced Changes in X-Ray Absorption (K-Edge) Energies of Manganese in Photosynthetic Membranes.  **In**: EXAFS and Near Edge Structure III (K.O. Hodgson, B. Hedman, and J.E. Penner-Hahn, Eds.) Springer, (Berlin, Heidelberg, New York, Tokyo) 1984, pp. 130-135. (Proceedings of the 3rd International EXAFS Conference, Stanford, CA, July 16-20, 1984).

1. K.K. Karukstis and K. Sauer. The Effects of Cation-Induced and pH-Induced Membrane Stacking on Chlorophyll Fluorescence Decay Kinetics. *Biochim*. *Biophys. Acta* **806**, 374-388 (1985).

1. M. Boska, N.V. Blough, and K. Sauer. The Effect of Mono- and Divalent Salts on the Rise and Decay Kinetics of EPR Signal II in Photosystem II Preparations from Spinach. *Biochim. Biophys. Acta* **808**, 132-139 (1985).

1. K. Sauer. Photosynthetic Light Reactions - Physical Aspects.  **In:** Encyclopedia of Plant Physiology [new series], vol. 19, Photosynthetic Membranes (L.A. Staehelin and C.J. Arntzen, Eds.). Springer-Verlag, Berlin, (1986) pp. 85-97.

1. J. Cole, M. Boska, N.V. Blough, and K. Sauer. Reversible and Irreversible Effects of Alkaline pH on Photosystem II Electron Transfer Reactions. *Biochim*. *Biophys. Acta* **848**, 41-47 (1986).

1. M. Boska, A. Yamagishi and K. Sauer. EPR Signal II in Cyanobacterial Photosystem II Reaction Center Complexes with and without the 40 kDa Chlorophyll-Binding Subunit. *Biochim. Biophys. Acta* **850**, 226-233 (1986).

1. V.K. Yachandra, R.D. Guiles, A. McDermott, R.D. Britt, S.L. Dexheimer, K.

Sauer, and M.P. Klein. The State of Manganese in the Photosynthetic Apparatus.

4. Structure of the Manganese Complex in Photosystem II Studied Using EXAFS Spectroscopy. The S_1_ State of the O_2_-Evolving Photosystem II Complex from Spinach. *Biochim. Biophys. Acta* **850**, 324-332 (1986).

1. V.K. Yachandra, R.D. Guiles, K. Sauer, and M.P. Klein. The State of Manganese in the Photosynthetic Apparatus. 5. The Chloride Effect in Photosynthetic Oxygen Evolution. Is Halide Coordinated to the EPR-active Manganese in the O_2_-Evolving Complex? Studies of the Structure of the Lowtemperature Multi-line EPR Signal. *Biochim. Biophys. Acta* **850**, 333-342 (1986).

1. V. Yachandra, R.D. Guiles, A. McDermott, R.D. Britt, J. Cole. S.L. Dexheimer, K. Sauer, and M.P. Klein. The State of Manganese in the Photosynthetic Apparatus Determined by X-Ray Absorption Spectroscopy. *J. de Physique* **47**, Colloque C8/1121-1128 (1986).

1. J. Cole, V.K. Yachandra, R.D. Guiles, A.E. McDermott, R.D. Britt, S.L. Dexheimer, K. Sauer and M.P. Klein. Assignment of the g=4.1 EPR Signal to Manganese in the S_2_ State of the Photosynthetic Oxygen-Evolving Complex: An X-Ray Absorption Edge Spectroscopy Study. *Biochim. Biophys. Acta* **890**, 395398 (1987).

1. K. Sauer and H. Scheer. Fluorescence Decay and Depolarization Kinetics Calculated Using Förster Inductive Resonance and the Molecular Coordinates for C-Phycocyanin. **In**: Progress in Photosynthesis Research, vol. I (J. Biggins, Ed.). Martinus Nijhoff Publ., Dordrecht, Netherlands, (1987) pp. 139-142.

1. J. Cole and K. Sauer. The Flash Number Dependence of EPR Signal II Decay as a Probe for Charge Accumulation in Photosystem II. **In**: Progress in Photosynthesis Research, vol. I (J. Biggins, Ed.). Martinus Nijhoff Publ., Dordrecht, Netherlands, (1987) pp. 569-572.

1. R.D. Guiles, V.K. Yachandra, A. McDermott, R.D. Britt, S.L. Dexheimer, K. Sauer, and M.P. Klein. Structural Features of the Manganese Cluster in Different States of the Oxygen Evolving Complex of Photosystem II: An X-ray Absorption Spectroscopy Study. **In**: Progress in Photosynthesis Research, vol. I (J. Biggins, Ed.). Martinus Nijhoff Publ., Dordrecht, Netherlands (1987) pp.561-564.

1. A.E. McDermott, V.R. Yachandra, R.D. Guiles, R.D. Britt, S.L. Dexheimer, K. Sauer, and M.P. Klein. Iron-X-Ray Absorption Spectra of Acceptors in PS I. **In**: Progress in Photosynthesis Research, vol. I (J. Biggins, Ed.). Martinus Nijhoff Publ., Dordrecht, Netherlands (1987) pp. 249-252.

1. A. McDermott, V.K. Yachandra, R.D. Guiles, R.D. Britt, S.L. Dexheimer, K. Sauer, and M.P. Klein. Characterization of the Mn-Containing O_2_-Evolving Complex from the Cyanobacterium Synechococcus using EPR and X-Ray Absorption Spectroscopy. **In:** Progress in Photosynthesis Research, vol. I (J. Biggins, Ed.). Martinus Nijhoff Publ., Dordrecht, Netherlands (1987) pp. 565568.

1. V.K. Yachandra, R.D. Guiles, A. McDermott, J. Cole, R.D. Britt, S.L. Dexheimer, K. Sauer, and M.P. Klein. The State of Manganese in the Photosynthetic Apparatus: An X-Ray Absorption Spectroscopy Study. **In**: Progress in Photosynthesis Research, vol. I (J. Biggins, Ed.). Martinus Nijhoff Publ., Dordrecht, Netherlands (1987), pp. 557-560.

1. R.D. Britt, K. Sauer, and M.P. Klein. Electron Spin Echo Studies of PS II Membranes. **In**: Progress in Photosynthesis Research, vol. I (J. Biggins, Ed.) Martinus Nijhoff Publ., Dordrecht, Netherlands (1987), pp. 573-576.

1. J. Cole and K. Sauer. The Flash Number Dependence of EPR Signal II Decay as a Probe for Charge Accumulation in Photosystem II. *Biochim. Biophys. Acta* **891,** 40-48 (1987).

1. S.T. Worland, A. Yamagishi, S. Isaac, K. Sauer, and J.E. Hearst. Labelling Quinone Binding Sites in Photosynthetic Reaction Centers: A 38 Kilodalton Protein Associated with the Acceptor Side of Photosystem II. Proc. Nat. Acad. Sci. USA **84**, 1774-1778 (1987).

1. K. Sauer, H. Scheer, and P. Sauer. Excitation Transfer Rates in C-Phycocyanin. Förster Transfer Calculations Based on Crystal Structure Data from Agmenellum quadruplicatum C-Phycocyanin. *Photochem. Photobiol.* **46**,

427-440 (1987).

1. J.L. Cole, V.K. Yachandra, A.E. McDermott, R.D. Guiles, R.D. Britt, S.L. Dexheimer, K. Sauer and M.P. Klein. Structure of the Manganese Complex of Photosystem II upon Removal of the 33 kDa Extrinsic Protein: An X-ray Absorption Spectroscopy Study. *Biochemistry* **26**, 5967-5973 (1987).

1. V. K. Yachandra, R.D. Guiles, A. E. McDermott, J.L. Cole, R. D. Britt, S.L. Dexheimer, K. Sauer and M.P. Klein. Comparison of the Structure of the Manganese Complex in the S_1_ and S_2_ States of the Photosynthetic O_2_-Evolving Complex: An X-Ray Absorption Spectroscopy Study. *Biochemistry* **26**, 5974-5981 (1987).

1. A.E. McDermott, R.D. Guiles, V.K. Yachandra, J. Cole, R.D. Britt, S.L. Dexheimer, K. Sauer, and M.P. Klein. X-Ray Absorption Spectroscopy of

Manganese and Iron in the Photosynthetic Apparatus. **In:** Biophysics and Synchrotron Radiation, (A. Bianconi and A. Congiu Castellano, Eds.) SpringerVerlag, Berlin, (1987) pp. 223-230.

1. K.S. Schanze and K. Sauer. Photoinduced Intramolecular Electron Transfer in Peptide Bridged Molecules. *J. Am. Chem. Soc*. **110,** 1180-1186 (1988).

1. P. Maxson, A.N. Glazer, and K.Sauer. Fluorescence Spectroscopy of Allophycocyanin Complexes from Synechococcus 6301 Strain AN 112. **In:** Photosynthetic Light Harvesting Systems, (S. Schneider and H. Scheer, Eds.), de Gruyter, Berlin (1988) pp.439-449.

1. Sauer and H. Scheer. Energy Transfer Calculations for Two C-Phycocyanins Based on Refined X-Ray Crystal Structure Coordinates of Chromophores. **In:**  Photosynthetic Light Harvesting Systems, (S. Schneider and H. Scheer, Eds.), de Gruyter, Berlin, (1988) pp. 507-511.

1. A.E. McDermott, V.K. Yachandra, R.D. Guiles, R.D. Britt, S.L. Dexheimer, K. Sauer, and M.P. Klein. Low Potential Iron-Sulfur Centers in Photosystem I**:** An X-ray Absorption Spectroscopy Study. *Biochemistry* **27**, 4013-4020 (1988)

1. A.E. McDermott, V.K. Yachandra, R.D. Guiles, J.L. Cole, S.L. Dexheimer, R.D.

Britt, K. Sauer and M.P. Klein. Characterization of the Mn O_2_-Evolving Complex and the Iron-Quinone Acceptor Complex in Photosystem II from a Thermophilic Cyanobacterium by EPR and X-Ray Absorption Spectroscopy. *Biochemistry* **27**, 4021-4031 (1988).

1. K. Sauer, R.D. Guiles, A.E. McDermott, J.L. Cole, V.K. Yachandra, J.-L. Zimmermann, M.P. Klein, S.L. Dexheimer and R.D. Britt. Spectroscopic Studies of Manganese Involvement in Photosynthetic Oxygen Evolution. *Chemica Scripta* **28A**, 87-91 (1988).

1. K. Sauer and H. Scheer. Excitation Transfer in C-Phycocyanin. Förster Transfer Rate and Exciton Calculations Based on New Crystal Structure Data for CPhycocyanin from *Agmenellum quadruplicatum* and *Mastigocladus laminosus. Biochim. Biophys. Acta* **936**, 157-170 (1988).

1. H.J.K. Keuper and K. Sauer. Effect of Photosystem II Reaction Center Closure on Nanosecond Fluorescence Relaxation Kinetics. *Photosynthesis Research* **20**, 85-103 (1989).

1. R.D. Britt, J.-L. Zimmermann, K. Sauer and M.P. Klein. Ammonia Binds to the Catalytic Mn of the Oxygen Evolution Complex of Photosystem II: Evidence by Electron Spin Echo Modulation Spectroscopy. *J. Am. Chem. Soc.* **111**, 3522-3532 (1989).

1. I. Mukerji and K. Sauer. Temperature-Dependent Steady-State and Picosecond Kinetic Fluorescence Measurements of a Photosystem I Preparation from Spinach. **In:** Photosynthesis, (W.R. Briggs, Ed.) Alan R. Liss, Inc., New York, 1989, pp. 105-122

1. A.E. McDermott, V.K. Yachandra, R.D. Guiles, K. Sauer, M.P. Klein, K.G. Parrett and J.H. Golbeck. EXAFS Structural Study of F_X_, the Low-Potential Fe-S Center in Photosystem I. *Biochemistry*, **28**, 8056-8059 (1989).

1. P. Maxson, K. Sauer, J. Zhou, D.A. Bryant and A.N. Glazer. Spectroscopic Studies of Cyanobacterial Phycobilisomes Lacking Core Polypeptides. *Biochim. Biophys. Acta* **977**, 40-51 (1989).

1. R.D. Britt, J.-L. Zimmermann, K. Sauer and M.P. Klein. ESEEM Studies of Ammonia Binding to the Catalytic Mn of the Water Oxidation Complex of Photosystem II. **In:** Pulsed EPR: A New Field of Applications, (C.P. Keijzers, E.J. Reijerse and J. Schmidt, Eds.) North Holland, Amsterdam, 1989, pp. 150155.

1. V.K. Yachandra, R.D. Guiles, A.E. McDermott, J.L. Cole, V.J. DeRose, J.-L. Zimmermann, K. Sauer and M.P. Klein. X-ray Absorption Spectroscopy of Mn in the Photosynthetic Apparatus. *Physica* *B* **158**, 78-80 (1989).

1. R.D. Guiles, J.-L. Zimmermann, A.E. McDermott, V.K. Yachandra, J.L. Cole,

S.L. Dexheimer, R.D. Britt, K. Wieghardt, U. Bossek, K. Sauer and M.P. Klein.

The S_3_ State of Photosystem II: Differences between the Structure of the Manganese Complex in the S_2_ and S_3_ States Detemined by X-ray Absorption Spectroscopy. *Biochemistry* **29**, 471-485 (1990).

1. R.D. Guiles, V.K. Yachandra, A.E. McDermott, J.L. Cole, S.L. Dexheimer, R.D. Britt, K. Sauer and M.P. Klein. The S_0_ State of Photosystem II Induced by Hydroxylamine: Differences between the Structure of the Manganese Complex in the S_0_ and S_1_ States Determined by X-ray Absorption Spectroscopy. *Biochemistry* **29**, 486-496 (1990).

1. K. Sauer. Photosystem II and Water Oxidation. **In**: Current Research in Photosynthesis, (M. Baltsheffsky, Ed.) Vol.I, pp 675-684, Kluwer Academic Publ., Dordrecht, The Netherlands (1990).

1. S.L. Dexheimer, K. Sauer and M.P. Klein. Parallel Polarization EPR Studies of the Oxygen-Evolving Complex of Photosystem II. **In**: Current Research in Photosynthesis, (M. Baltsheffsky, Ed.) Vol.I, pp 761-764, Kluwer Academic Publ., Dordrecht, The Netherlands (1990).

1. R.D. Britt, V.J. DeRose, V.K. Yachandra, D.H. Kim, K. Sauer and M.P. Klein. Pulsed EPR Studies of the Manganese Center of the Oxygen-Evolving Complex of Photosystem II. **In**: Current Research in Photosynthesis, (M. Baltsheffsky, Ed.) Vol.I, pp 769-772, Kluwer Academic Publ., Dordrecht, The Netherlands (1990).

1. R.D. Guiles, V.K. Yachandra, A.E. McDermott, V.J. DeRose, J.-L. Zimmermann, K. Sauer and M.P. Klein. Structures and Oxidation States of Mn in Several S-States of Photosystem II Determined by X-Ray Absorption Spectroscopy. **In**: Current Research in Photosynthesis, (M. Baltsheffsky, Ed.) Vol.I, pp 789-792, Kluwer Academic Publ., Dordrecht, The Netherlands (1990).

1. I. Mukerji and K. Sauer. A Spectroscopic Study of a Photosystem I Antenna Complex. **In**: Current Research in Photosynthesis, (M. Baltsheffsky, Ed.) Vol.II, pp 321-324, Kluwer Academic Publ., Dordrecht, The Netherlands (1990).

1. J.C. Gingrich, G.E. Gasparich, K. Sauer and D.A. Bryant. Nucleotide Sequence and Expression of the Two Genes Encoding the D2 Protein and the Single Gene Encoding the CP43 Protein of Photosystem II in the Cyanobacterium *Synechococcus* sp. PCC 7002. *Photosynthesis Research* **24**,137-150 (1990).

1. W.F. Beck, M. Debreczeny, X. Yan and K. Sauer. Picosecond Transient Absorption and Dichroism Studies on Excitation Transfer in Allophycocyanin. **In:** Ultrafast Phenomena VII, (C.B. Harris, E.P. Ippen, G.A. Mourou and A.H. Zewail, Eds.) Springer Series in Chemical Physics 53, pp 535-537, SpringerVerlag, Berlin (1990).

1. D.H. Kim, R.D. Britt, M.P. Klein and K. Sauer. The g=4.1 Signal of the S_2_ State of the Photosynthetic Oxygen-Evolving Complex Arises from a Multinuclear Mn Cluster. *J. Amer. Chem. Soc.* **112**, 9389-9391 (1990).

1. V.J. DeRose, V.K. Yachandra, A.E. McDermott, R.D. Britt, K. Sauer and M.P. Klein. Nitrogen Ligation to Manganese in the Photosynthetic Oxygen-Evolving Complex: Continuous-Wave and Pulsed EPR Studies of Photosystem II Particles Containing ^14^N or ^15^N. *Biochemistry* **30**, 1335-1341 (1991).

1. J.W. Park and K. Sauer. Subunit Selective Bleaching of C-Phycocyanin from *Synechococcus* PCC 6301 Strain AN 112 by Cu^2+^ and Sodium Perchlorate. *Biochim. Biophys. Acta* **1056**, 181-189 (1991).

1. R.D. Britt, K. Sauer, M.P. Klein, D.B. Knaff, A. Kriauciunas, C.-A. Yu, L. Yu and R. Malkin. Electron Spin Echo Envelope Modulation Spectroscopy Supports the Suggested Coordination of Two Histidine Ligands to the Rieske Fe-S Centers of the Cytochrome *b_6_ f* Complex of Spinach and the Cytochrome *bc_1_* Complexes of *Rhodospirillum rubrum*, *Rhodobacter sphaeroides* R-26 and Bovine Heart Mitochondria. *Biochemistry* **30**, 1892-1901 (1991).

1. L.J. Juszczak, B.A. Zilinskas, N.E. Geacintov, J. Breton and K. Sauer. Orientation and Linear Dichroism of *Mastigocladus laminosus* Phycocyanin Trimer and *Nostoc* sp. Phycocyanin Dodecamer in Stretched Polyvinylalcohol Films. *Biochim. Biophys. Acta* **1058**,363-373 (1991).

1. H. Dau and K. Sauer. Electric Field Effect on Chlorophyll Fluorescence and Its Relation to Photosystem II Charge Separation Reactions Studied by a Salt-Jump Technique. *Biochim. Biophys. Acta* **1098**, 49-60 (1991).

1. K. Sauer, V.K. Yachandra, R.D. Britt and M.P. Klein. The Photosynthetic Water Oxidation Complex Studied by EPR and X-Ray Absorption Spectroscopy. **In**: Manganese Redox Enzymes, (V.L. Pecoraro, Ed.), VCH Publishers, New York, pp. 141-175 (1992).

1. D.H. Kim, R.D. Britt, M.P. Klein and K. Sauer. The Manganese Site of the

Photosynthetic Oxygen-Evolving Complex Probed by EPR Spectroscopy of Oriented Photosystem II Membranes: The *g* = 4 and *g* = 2 Multiline Signals. *Biochemistry* **31**, 541-547 (1992).

1. W.F. Beck and K. Sauer. Energy Transfer and Exciton-State Relaxation Processes in Allophycocyanin. *J. Phys. Chem.* **96**, 4658-4666; 7146 (1992).

1. H. Dau and K. Sauer. Electric Field Effect on the Picosecond Fluorescence of Photosystem II and Its Relation to the Energetics and Kinetics of Primary Charge Separation. *Biochim. Biophys. Acta* **1102**, 91-106 (1992).

1. Y.M. Gindt, J. Zhou, D.A. Bryant and K. Sauer. Core Mutations of *Synechococcus* sp PCC7002 Phycobilisomes: A Spectroscopic Study. J. *Photochem. Photobiol. B* **15**, 75-89 (1992) .

1. R.D. Britt, G.A. Lorigan, K. Sauer, M.P. Klein and J.-L. Zimmermann. The *g*=2 Multiline EPR Signal of the S2 State of the Photosynthetic Oxygen-Evolving Complex Originates from a Ground Spin State. *Biochim. Biophys. Acta* **1140**, 95101 (1992).

1. H. Dau and K. Sauer. Electric Field Effect on the Primary Charge Separation of PS II - Comparison with Electron Transfer Theories. **In:** Research in Photosynthesis, (N. Murata. ed) Vol.II, pp 239-242, Kluwer Academic Publ., Dordrecht, The Netherlands (1992).

1. V.K. Yachandra, V.J. DeRose, M.J. Latimer, I. Mukerji, K. Sauer and M.P. Klein. A Structural Model for the Photosynthetic Oxygen Evolving Manganese Complex. **In:** Research in Photosynthesis, (N. Murata. ed) Vol.II, pp 281-287, Kluwer Academic Publ., Dordrecht, The Netherlands (1992).

1. I. Mukerji and K. Sauer. Energy Transfer Dynamics of an Isolated Light Harvesting Complex of Photosystem I from Spinach: Time-Resolved Fluorescence Measurements at 295K and 77K. *Biochim. Biophys. Acta* **1142**, 311-320 (1993).

1. V.K. Yachandra, V.J. DeRose, M.J. Latimer, I. Mukerji, K. Sauer and M.P. Klein. Where Plants Make Oxygen: A Structural Model for the Photosynthetic Oxygen Evolving Manganese Cluster. *Science* **260**, 675-679 (1993).

1. V.K. Yachandra, V.J. DeRose, M.J. Latimer, I. Mukerji, K. Sauer and M.P. Klein. A Structural Model for the Photosynthetic Oxygen Evolving Manganese Complex. *Jpn. J. Appl. Phys.* **32** (Suppl. 32-2) 523-526 (1993).

1. M.P. Debreczeny, K. Sauer, J. Zhou and D.A. Bryant. Monomeric CPhycocyanin at Room Temperature and 77K: Resolution of the Absorption and Fluorescence Spectra of Individual Chromophores and the Energy Transfer Rate Constants. *J. Phys. Chem.* **97**, 9852-9862 (1993).

1. M.P. Klein, K. Sauer and V.K. Yachandra. Perspectives on the Structure of the Photosynthetic Oxygen Evolving Manganese Complex and Its Relation to the Kok Cycle. *Photosyn. Res.* **38**, 265-277 (1994).

1. W. Liang, M.J. Latimer, H. Dau, T.A. Roelofs, V.K. Yachandra, K. Sauer and M.P. Klein. Correlation between Structure and Magnetic Spin State of the Manganese Cluster in the Oxygen-Evolving Complex of Photosystem II in the S2 State: Determination by X-ray Absorption Spectroscopy. *Biochemistry* **33**, 49234932 (1994).

1. V.J. DeRose, I. Mukerji, M.J. Latimer, V.K. Yachandra, K. Sauer and M.P. Klein. Comparison of the Manganese Oxygen-Evolving Complex in Photosystem II of Spinach and *Synechococcus* sp. with Multinuclear Manganese Model Compounds by X-ray Absorption Spectroscopy. *J. Am. Chem. Soc.* **116**, 5239-5249 (1994).

1. A.N. Glazer, Y.M. Gindt, C.F. Chan and K. Sauer. Selective Disruption of Energy Flow from Phycobilisomes to Photosystem I. *Photosyn Res.* **40**, 167-173 (1994).

1. Y.M. Gindt, J. Zhou, D.A. Bryant and K. Sauer. Spectroscopic Studies of Phycobilisome Subcore Preparations Lacking Key Core Chromophores: Assignment of Excited State Energies to the Lcm, β18, and α^AP-B^ Chromophores. *Biochim. Biophys Acta* **1186**, 153-162 (1994).

1. I. Mukerji, J.C. Andrews, V.J. DeRose, M.J. Latimer, V.K. Yachandra, K. Sauer and M.P. Klein. Orientation of the Oxygen-Evolving Manganese Complex in a Photosystem II Membrane Preparation: An X-ray Absorption Spectroscopy Study. Biochemistry **33**, 9712-9721 (1994).

1. K. Sauer. Why Spectroscopy? Which Spectroscopy? *In:* Methods in Enzymology, vol. **246**, Biochemical Spectroscopy (K. Sauer, Ed.), 1995, Academic Press, Orlando, FL, pp. 1-10.

1. H. Dau, J.C. Andrews, T.A. Roelofs, M.J. Latimer, W. Liang, V.K. Yachandra, K. Sauer and M.P. Klein. Structural Consequences of Ammonia Binding to the Manganese Center of the Photosynthetic Oxygen-Evolving Complex: An X-Ray Absorption Spectroscopy Study of Isotropic and Oriented Photosystem II Particles. *Biochemistry* **34**, 5274-5287 (1995).

1. M. Debreczeny, K. Sauer, J. Zhou and D.A. Bryant. Comparison of Calculated and Experimentally Resolved Rate Constants for Excitation Energy Transfer in C-Phycocyanin. Part I: Monomers. *J. Phys. Chem.* **99**, 8412-8419 (1995).

1. M. Debreczeny, K. Sauer, J. Zhou and D.A. Bryant. Comparison of Calculated and Experimentally Resolved Rate Constants for Excitation Energy Transfer in CPhycocyanin. Part II: Trimers. *J. Phys. Chem.* **99**, 8420-8431 (1995).

1. V.J. DeRose, M.J. Latimer, J.-L. Zimmermann, I. Mukerji, V.K. Yachandra, K. Sauer and M.P. Klein. Fluoride Substitution in the Mn Cluster from Photosystem II: EPR and X-ray Absorption Spectroscopy Studies. *Chem. Phys.* **194**, 443-459 (1995).

1. J.C. Andrews, R. Cinco, H. Dau, M.J. Latimer, W. Liang, T.A. Roelofs, A. Rompel, K. Sauer, V.K. Yachandra and M.P. Klein. Photosynthetic Water Oxidation: Structural Insights to the Catalytic Manganese Complex. *Physica B* **208&209**, 657-659 (1995).

1. M.J. Latimer, V.J. DeRose, I. Mukerji, V.K. Yachandra, K. Sauer and M.P. Klein. Evidence for the Proximity of Calcium to the Manganese Cluster of Photosystem II: Determination by X-Ray Absorption Spectroscopy. *Biochemistry*, 34, 1089810909 (1995).

1. W. Liang, T.A. Roelofs, G.T. Olsen, M.J. Latimer, R.M. Cinco, A. Rompel, K.

Sauer, V.K. Yachandra and M.P. Klein. S3 State of the O2 Evolving Complex:

Structure of the Mn Complex of Photosystem II Determined by X-ray Absorption Spectroscopy. *In:* Photosynthesis: from Light to Biosphere. (P. Mathis, Ed.), 1995, vol II, Kluwer, Dordrecht, pp 413-416.

1. M.J. Latimer, H. Dau, W. Liang, J.C. Andrews, T.A. Roelofs, R.M. Cinco, A. Rompel, K. Sauer, V.K. Yachandra and M.P. Klein. Recent Advances toward a Structural Model for the Photosynthetic Oxygen-Evolving Manganese Cluster. *In:* Photosynthesis: from Light to Biosphere. (P. Mathis, Ed.), 1995, vol II, Kluwer, Dordrecht, pp 417-420.

1. H. Dau, J.C. Andrews, T.A. Roelofs M.J. Latimer, W. Liang, K. Sauer, V.K. Yachandra and M.P. Klein. X-ray Absorption Linear Dichroism Spectroscopy (XALDS) on the PSII Manganese Complex - Methodological Aspects. *In:* Photosynthesis: from Light to Biosphere. (P. Mathis, Ed.), 1995, vol II, Kluwer, Dordrecht, pp 271-274.

1. T.A. Roelofs, W. Liang, M.J. Latimer, R.M. Cinco, A. Rompel, J.C. Andrews, V.K. Yachandra, K. Sauer and M.P. Klein. Manganese Oxidation States of the Flash-Induced S-States of Photosystem II. *In:* Photosynthesis: from Light to Biosphere. (P. Mathis, Ed.), 1995, vol II, Kluwer, Dordrecht, pp 459-462.

1. J. Steiger and K. Sauer. Electric Field Effects on Fluorescence Emission of *Rb.*

*sphaeroides* Chromatophores. *In:* Photosynthesis: from Light to Biosphere. (P. Mathis, Ed.), 1995, vol I, Kluwer, Dordrecht, pp 735-738.

1. K. Sauer and M. Debreczeny. Fluorescence. *In:* Biophysical Techniques in Photosynthesis, (A.J. Hoff and J. Amesz, Eds.), 1996, Kluwer, Dordrecht, pp 4161.

1. H. Dau and K. Sauer. Exciton Equilibration and Photosystem II Exciton Dynamics - a Fluorescence Study on Photosystem II Membrane Particles of Spinach. *Biochim. Biophys. Acta* **1273**, 175-190 (1996).

1. T.A. Roelofs, W. Liang, M.J. Latimer, R.M. Cinco, A. Rompel, J.C. Andrews, K.

Sauer, V.K. Yachandra and M.P. Klein. Oxidation States of the Manganese Cluster during the Flash-Induced S-State Cycle of the Photosynthetic Oxygen Evolving Complex. *Proc. Nat. Acad. Sci., USA*, **93,** 3335-3340 (1996).

1. A. Freer, S. Prince, K. Sauer, M. Papiz, A. Hawthornthwaite-Lawless, G. McDermott, R. Cogdell and N. Isaacs. Pigment-Pigment Interactions and Energy Transfer in the Antenna Complex of the Photosynthetic Bacterium *Rhodopseudomonas acidophila. Structure* **4**, 449-462 (1996).

1. K. Sauer, R.J. Cogdell, S.M. Prince, A.A. Freer, N.W. Isaacs and H. Scheer.

Structure-Based Calculations of the Optical Spectra of the LH2

Bacteriochlorophyll-Protein Complex from *Rhodopseudomonas acidophila*. *Photochem. Photobiol*.. **64**, 564-576 (1996).

1. V.K. Yachandra, K. Sauer and M.P. Klein. Manganese Cluster in Photosynthesis: Where Plants Oxidize Water to Dioxygen. *Chem. Rev.* **96**, 2927-2950 (1996).

1. M.J. Latimer, H. Dau, W. Liang, J.C. Andrews, T.A. Roelofs, R.M. Cinco, A. Rompel, K. Sauer, V.K. Yachandra and M.P. Klein. Recent Advances toward a Structural Model for the Photosynthetic Oxygen-Evolving Manganese Cluster. *In*: Applications of Synchrotron Radiation in Industrial, Chemical and Materials Science, (L.J. Terminello, K.L. D'Aminco and D.K. Shuh), 1996, Plenum, NY, pp 141-147.

1. A. Rompel, J.C. Andrews, R.M. Cinco, M.W. Wemple, G. Christou, N.A. Law, V.L. Pecoraro, K. Sauer, V.K. Yachandra and M.P. Klein. Chlorine K-Edge Xray Absorption Spectroscopy as a Probe of Chlorine-Manganese Bonding: Model Systems with Relevance to the Oxygen Evolving Complex in Photosystem II. *J. Am. Chem. Soc.* **119**, 4465-4470 (1997).

1. M.F.J. Talbot and K. Sauer. Spectrofluorimetric Method for the Determination of Large Chlorophyll a/b Ratios. *Photosynthesis Research*, **53**, 73-79 (1997).

1. J. Messinger, J.H. Robblee, W.O. Yu, K. Sauer, V.K. Yachandra and M.P. Klein. The S0 State of the Oxygen-Evolving Complex in Photosystem II Is Paramagnetic: Detection of an EPR Multiline Signal. *J. Am. Chem. Soc.*, 119,

11349-11350 (1997).

1. W.U. Wang, R. Mathies and K. Sauer. Photosynthetic Pigment Analysis and Metallic Modified Chlorophylls. *Berkeley Sci*. **1**, 51-54 (1997).

1. A. Rompel, R.M. Cinco, M.J. Latimer, A.E. McDermott, R.D. Guiles, A. Quintanilha, R.M. Krauss, K. Sauer, V.K. Yachandra and M.P. Klein. Sulfur Kedge X-ray Absorption Spectroscopy: A Spectroscopic Tool to Examine the Redox State of S-Containing Metabolites *in Vivo*. *Proc. Natl. Acad. Sci., U.S*. **95**, 6122-6127 (1998).

1. R.M. Cinco, J.H. Robblee, A. Rompel, C. Fernandez, V.K. Yachandra, K. Sauer and M.P. Klein. Strontium EXAFS Reveals the Proximity of Calcium to the Manganese Cluster of Oxygen-Evolving Photosystem II. *J. Phys. Chem. B* **102**, 8248-8256 (1998).

1. M.J. Latimer, V.J. DeRose, V.K. Yachandra, K. Sauer and M.P. Klein. Structural Effects of Calcium Depletion on the Manganese Cluster of Photosystem II: Determination by X-ray Absorption Spectroscopy. *J. Phys. Chem. B* **102**, 82578265 (1998).

1. McFarlane, U. Bergmann, P. Glatzel, S.P. Cramer, K. Sauer, M.P. Klein, and V.K. Yachandra. Refined Model of the Oxidation States and Structures of the Mn/Ca/Cl Cluster of the Oxygen Evolving Complex of Photosystem II. *In:* Photosynthesis: Mechanisms and Effects. (G. Garab, Ed.), 1998, vol. II, Kluwer Academic Publishers, Dordrecht, pp 1273-1278.

1. J. Messenger, J.H. Robblee, C. Fernandez, R.M. Cinco, H. Visser, U. Bergmann, P. Glatzel, S.P. Cramer, K.A. Campbell, J.M. Peloquin, R.D. Britt, K. Sauer, V.K. Yachandra and M.P. Klein. Oxidation States and Structure of the Manganese Cluster in the S_0_ State of the Oxygen Evolving Complex. *In:* Photosynthesis: Mechanisms and Effects. (G. Garab, Ed.), 1998, vol. II, Kluwer Academic Publishers, Dordrecht, pp 1279-1282.

1. C. Fernandez, R.M. Cinco, J.H. Robblee, J. Messinger, S.A. Pizarro, K. Sauer, M.P. Klein and V.K. Yachandra. Calcium and Chloride Cofactors of the Oxygen Evolving Complex – X-ray Absorption Spectroscopy Evidence for a Mn/Ca/Cl Heteronuclear Cluster. *In:* Photosynthesis: Mechanisms and Effects. (G. Garab, Ed.), 1998, vol. II, Kluwer Academic Publishers, Dordrecht, pp 1399-1402.

1. E. Bellacchio and K. Sauer. Temperature Dependence of Optical Spectra of Bacteriochlorophyll *a* in Solution and in Low-Temperature Glasses*. J. Phys. Chem. B* **103**, 2279-2290 (1999).

1. E. Anxolabéhère-Mallart, H. Visser, U. Bergmann, S.P. Cramer, J.-J. Girerd, K. Sauer, V.K. Yachandra, and M.P. Klein. Mn K-edge XANES studies and high resolution Mn K_β_ fluorescence spectroscopy of manganese dinuclear complexes. Investigation of three different oxidation states and relevance to the Oxygen Evolving Center of PSII. *J. Inorg. Biochem*. **74**, 66 (1999). [Abstract]

1. J.H. Robblee, J. Messinger, C. Fernandez, R.M. Cinco, H. Visser, K.L.

McFarlane, U. Bergmann, P. Glatzel, S.P. Cramer, K. Sauer, M.P. Klein and V.K. Yachandra. Oxidation states of the Mn cluster in the S-states of the oxygenevolving complex of PS II. *J. Inorg. Biochem*. **74**, 278 (1999). [Abstract]

1. R.M. Cinco, J.H. Robblee, A. Rompel, C. Fernandez, K. Sauer, V.K. Yachandra and M.P. Klein. Proximity of Calcium to the Manganese Cluster of the Photosynthetic Oxygen-Evolving Complex Determined from Strontium EXAFS*. J. Synchrotron Rad*. 6, 419-420 (1999).

1. R.M. Cinco, A. Rompel, H. Visser, G. Aromi, G. Christou, K. Sauer, M.P. Klein and V.K. Yachandra. Comparison of the Manganese Cluster in Oxygen-Evolving Photosystem II with Distorted Cubane Manganese Compounds through X-ray Absorption Spectroscopy. *Inorg. Chem.* **38**, 5988-5998 (1999).

1. W. Liang, T.A. Roelofs, R.M. Cinco, A. Rompel, M.J. Latimer, W.O. Yu, K. Sauer, M.P. Klein and V.K. Yachandra. Structural Change of the Mn Cluster during the S2 to S3 State Transition of the Oxygen-Evolving Complex of Photosystem II. Does it Reflect the Onset of Water/Substrate Oxidation? Determination by X-ray Absorption Spectroscopy. *J. Amer. Chem. Soc.* **122**, 3399-3412 (2000).

1. S.A. Pizarro and K. Sauer. Spectroscopic Study of the Light-Harvesting Protein C-Phycocyanin Associated with Colorless Linker Peptides*. Photochem. Photobiol.* **73**, 556-563 (2001).

1. H. Visser, E. Anxolabéhère-Mallart, U. Bergmann, P. Glatzel, J.H. Robblee, S.P. Cramer, J.-J. Girerd, K. Sauer, M.P. Klein and V.K. Yachandra. Mn K-Edge XANES and Kβ XES Studies of Two Mn-Oxo Binuclear Complexes: Investigation of Three Different Oxidation States Relevant to the Oxygen Evolving Complex of Photosystem II. *J. Amer. Chem. Soc.* **123**, 7031-7039 (2001).

1. J. Messenger, J.H. Robblee, U. Bergmann, C. Fernandez, P. Glatzel, H. Visser, R.M. Cinco, K.L. McFarlane, E. Bellacchio, S.A. Pizarro, S.P. Cramer, K. Sauer,

M.P. Klein and V.K. Yachandra. Absence of Mn-Centered Oxidation in the S2-S3 Transition: Implications for the Mechanism of Photosynthetic Water Oxidation. *J. Amer. Chem. Soc.* **123**, 7804-7820 (2001).

1. H. Visser, A.E. Curtright, J.K. McCusker and K. Sauer. An Attenuated Total Reflection Design for *in Situ* FTIR Spectroelectrochemical Studies. *Anal. Chem*. **73**, 4374-4378 (2001).

1. R.M. Cinco, J.H. Robblee, J. Messinger, C. Fernandez, K.L. McFarlane, S.A. Pizarro, K. Sauer and V.K. Yachandra. Ca cofactor of the water-oxidation complex: Evidence for a Mn/Ca heteronuclear cluster*. Proceedings 12th International Congress on Photosynthesis*, Brisbane, Australia. S10-004 (2001).

1. J. Messinger, J.H. Robblee, U. Bergmann, C.Fernandez, P. Glatzel, S. Isgandarova, B. Hanssum, G. Renger, S.P. Cramer, K. Sauer and V.K. Yachandra. Manganese oxidation states in photosystem II*. Proceedings 12th International Congress on Photosynthesis*, Brisbane, Australia. S10-019 (2001).

1. J.H. Robblee, J. Messinger, R.M. Cinco, C. Fernandez, H. Visser, S.A. Pizarro, K. Sauer and V.K. Yachandra. Structure of the Mn complex involved in photosynthetic water oxidation: New insights derived from X-ray absorption spectroscopy. *Proceedings 12th International Congress on Photosynthesis*, Brisbane, Australia. S13-014 (2001).

1. J.H. Robblee, J. Messinger, R.M. Cinco, K.L. McFarlane, C. Fernandez, S.A. Pizarro, K. Sauer and Y.K. Yachandra. The Mn Cluster in the S_0_ State of the Oxygen-Evolving Complex of Photosystem II Studied by EXAFS Spectroscopy: Are There Three Di-µ-oxo-bridged Mn_2_ Moieties in the Tetranuclear Mn Complex? *J. Amer. Chem. Soc.* **124,** 7459-7471 (2002).

1. K. Sauer and V.K. Yachandra. A Possible Evolutionary Origin for the Mn_4_ Cluster of the Photosynthetic Water Oxidation Complex from Natural MnO_2_ Precipitates in the Early Ocean. *Proc. Natl. Acad. Sci. USA* **99**, 8631-8636 (2002).

1. H. Visser, C.E. Dubé, W.H. Armstrong, K. Sauer and V.K. Yachandra. FTIR Spectra and Normal-Mode Analysis of an Adamantane-like Complex in Two Electrochemically Prepared Oxidation States: Relevance to the Oxygen-Evolving Complex of Photosystem II. *J. Amer. Chem. Soc.* **124**, 11008-11017 (2002).

1. R.M. Cinco, K.L. McFarlane Holman, J.H. Robblee, J. Yano, S.A. Pizarro, E. Bellacchio, K. Sauer and V.K. Yachandra. Calcium EXAFS Establishes the MnCa Cluster in the Oxygen-Evolving Complex of Photosystem II. *Biochemistry*, 41, 12928-12933 (2002).

1. Pizarro, S. A., Visser, H., Cinco, R. M., Robblee, J. H., Pal, S., Mukhopadhyay, S., Mok, H. J., Sauer, K., Wieghardt, K., Armstrong, W. H., Yachandra, V. K. (2004) Chloride ligation in inorganic Mn model compounds relevant to Photosystem II studied using X-ray absorption spectroscopy. *J. Biol. Inorg. Chem.* **9**, 247-255.

1. Sauer, K., Yachandra, V. K. (2004) The Water-Oxidation Complex in Photosynthesis. *Biochim. Biophys. Acta*, **1665**, 140-148.

1. Yano, J., Sauer, K., Girerd, J.-J., Yachandra, V. K. (2004) Single Crystal X- and Q-Band EPR Spectroscopy of a Binuclear Mn_2_(III,IV) Complex Relevant to the Oxygen-Evolving Complex of Photosystem II. *J. Am. Chem. Soc.* **126**, 74867495.

1. Cinco, R. M., Robblee, J. H., Messinger, J., Fernandez, C., McFarlane, K. L., Sauer, K., Yachandra, V. K. (2004) Orientation of Calcium in the Mn_4_Ca Cluster of the Oxygen-Evolving Complex Determined Using Polarized Strontium EXAFS of Photosystem II Membranes. *Biochemistry*, **43**, 13271-13282.

1. Sauer, K., Yano, J., Yachandra, V. K. (2005) X-ray Spectroscopy of the Mn_4_Ca Cluster in the Water-Oxidation Complex of Photosystem II. *Photosyn. Res.* **85**, 73-86.

1. Yano, J., Kern, J., Irrgang, K.-D., Latimer, M. J., Bergmann, U., Glatzel, P., Pushkar, Y., Biesiadka, J., Loll, B., Sauer, K., Messinger, J., Zouni, A., Yachandra, V. K. (2005) X-ray Damage to the Mn_4_Ca Complex in Photosystem II Crystals: A Case Study for Metallo-Protein X-ray Crystallography. *Proc. Natl. Acad. Sci. USA*. **102**, 12047-12052.

1. Yano, J., Pushkar, Y., Glatzel, P., Lewis, A., Sauer, K., Messinger, J., Bergmann,

U., Yachandra, V. K. (2005) High-resolution Mn EXAFS of the Oxygen-evolving Complex in Photosystem II: Structural Implications for the Mn_4_Ca Cluster. *J. Am. Chem. Soc*. **127**, 14974-14975.

1. Yano, J., Kern, J., Sauer, K., Latimer, M. J., Pushkar, Y., Biesiadka, J., Loll, B., Saenger, W., Messinger, J., Zouni, A., Yachandra, V. K. (2006) Where Water is Oxidized to Dioxygen: Structure of the Photosynthetic Mn_4_Ca Cluster. *Science*, **314**, 821-825.

1. Pushkar, Y., Yano, J., Glatzel, P., Messinger, J., Lewis, A., Sauer, K., Bergmann, U., Yachandra, V. K. (2007) Structure and Orientation of the Mn_4_Ca Cluster in Photosystem II Membranes Determined by Polarized Range-Extended X-ray Absorption Spectroscopy. *J. Biol. Chem.* **282***,* 7198-7208.

1. Pushkar, Y., Yano, J., Glatzel, P., Messinger, J., Lewis, A., Sauer, K., Bergmann, U., Yachandra, V. K. (2007) Polarized Range-Extended X-ray Absorption Spectroscopy of Oriented Photosystem II Membranes in the S_1_ State. *American Institute of Physics, XAFS13 Conference Proceedings*. Vol 882, 346-348.

1. Sauer, K., Yano, J., Yachandra, V. K. (2008) X-ray Spectroscopy of the Photosynthetic Oxygen-Evolving Complex*. Coord. Chem. Rev.* **252**, 318-335.

1. Pushkar, Y., Yano, J., Sauer, K. Boussac, A., Yachandra, V. K. (2008) Structural Changes in the Mn_4_Ca Cluster and the Mechanism of Photosynthetic Water Splitting. *Proc. Natl. Acad. Sci. U. S. A.* **105**, 1879-1884.
